# C_3_-Alkylation of Imidazo[1,2-a]pyridines via Three-Component Aza-Friedel–Crafts Reaction Catalyzed by Y(OTf)_3_

**DOI:** 10.3390/molecules29153463

**Published:** 2024-07-24

**Authors:** Kai Yang, Cai-Bo Chen, Zhao-Wen Liu, Zhen-Lin Li, Yu Zeng, Zhao-Yang Wang

**Affiliations:** 1 College of Pharmacy, Gannan Medical University, Ganzhou 341000, China; ccb18046698424@outlook.com (C.-B.C.); liuzhaowenyifan@126.com (Z.-W.L.); lizhenlin1@gmu.cn (Z.-L.L.); 2 School of Chemistry, South China Normal University, Guangzhou Key Laboratory of Analytical Chemistry for Biomedicine, GDMPA Key Laboratory for Process Control and Quality Evaluation of Chiral Pharmaceuticals, Key Laboratory of Theoretical Chemistry of Environment, Ministry of Education, Guangzhou 510006, China; 2023022534@m.scnu.edu.cn

**Keywords:** imidazo[1,2-a]pyridines, aza-Friedel–Crafts reaction, Lewis acid catalyst, alkylation, three-component reaction

## Abstract

As an important class of nitrogen-containing fused heterocyclic compounds, imidazo[1,2-a]pyridines often exhibit significant biological activities, such as analgesic, anticancer, antiosteoporosis, anxiolytic, etc. Using Y(OTf)_3_ as a Lewis acid catalyst, a simple and efficient method has been developed for the synthesis of C_3_-alkylated imidazo[1,2-a]pyridines through the three-component aza-Friedel–Crafts reaction of imidazo[1,2-a]pyridines, aldehydes, and amines in the normal air atmosphere without the protection of inert gas and special requirements for anhydrous and anaerobic conditions. A series of imidazo[1,2-a]pyridine derivatives were obtained with moderate to good yields, and their structures were confirmed by ^1^H NMR, ^13^C NMR, and HRMS. Furthermore, this conversion has the advantages of simple operation, excellent functional group tolerance, high atomic economy, broad substrate scope, and can achieve gram-level reactions. Notably, this methodology may be conveniently applied to the further design and rapid synthesis of potential biologically active imidazo[1,2-a]pyridines with multifunctional groups.

## 1. Introduction

**Figure 1:**
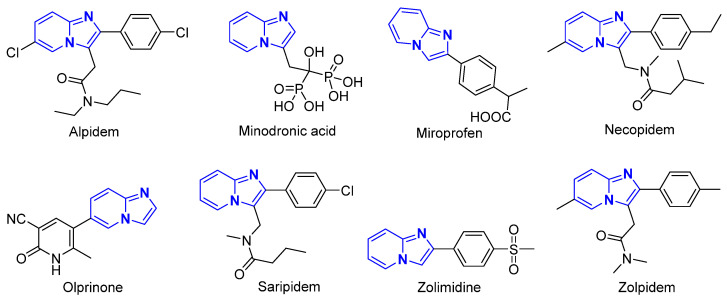
Representative drugs containing imidazo[1,2-a]pyridine scaffold.

As an important class of nitrogen-containing fused heterocyclic compounds, imidazo[1,2-a]pyridines often exhibit significant biological activities (e.g., analgesic, anticancer, antiosteoporosis, and anxiolytic) and have been explored as potential candidates for different biological activities [1,2,3]. For example, several marketed drugs, including Alpidem, Minodronic acid, Miroprofen, Necopidem, Olprinone, Saripidem, Zolimidine, and Zolpidem, contain the imidazo[1,2-a]pyridine scaffold in their molecular structures (Figure 1) [4,5,6].

Importantly, it is believed that imidazo[1,2-a]pyridine scaffold [7,8] is among the priority pharmacophores in drug research. Therefore, due to the significant pharmacological activities and the frequent occurrence in important drugs, the synthesis of imidazo[1,2-a]pyridine derivatives containing a variety of substituents has gained considerable attention recently [9,10,11]. Of course, it should be pointed out that, although imidazo[1,2-a]pyridine derivatives with diverse structures have been constantly designed and synthesized, and successfully applied in various biological activity studies [12], the green and efficient synthesis methods of some special structurally functionalized imidazo[1,2-a]pyridine derivatives still need further research and enrichment for the practical drug development.

**Figure 2:**
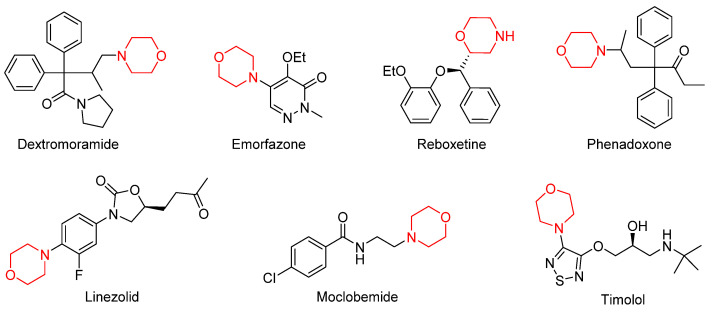
Some drugs containing morpholine unit.

At the same time, as a six-membered heterocycle simultaneously containing both nitrogen and oxygen atoms, morpholine (1,4-tetrahydro-oxazine) is frequently exploited in the field of medicinal chemistry for its advantageous physicochemical, biological, and metabolic properties [13,14]. Especially, some appropriately substituted morpholine derivatives possess a wide range of biological actions, including anti-inflammatory, antimicrobial, and anticancer activity, etc. [15]. Importantly, many approved drugs, clinical candidates, and bioactive molecules, such as Dextromoramide, Emorfazone, Reboxetine, Phenadoxone, Linezolid, Moclobemide, and Timolol (Figure 2) [16], also contain the structural unit of morpholine. Therefore, the introduction of morpholine unit into the fused heterocyclic molecules, such as imidazo[1,2-a]pyridines, to confer compounds with desirable drug-like properties is of importance in the search for new biologically active candidates [17].

**Scheme 1:**
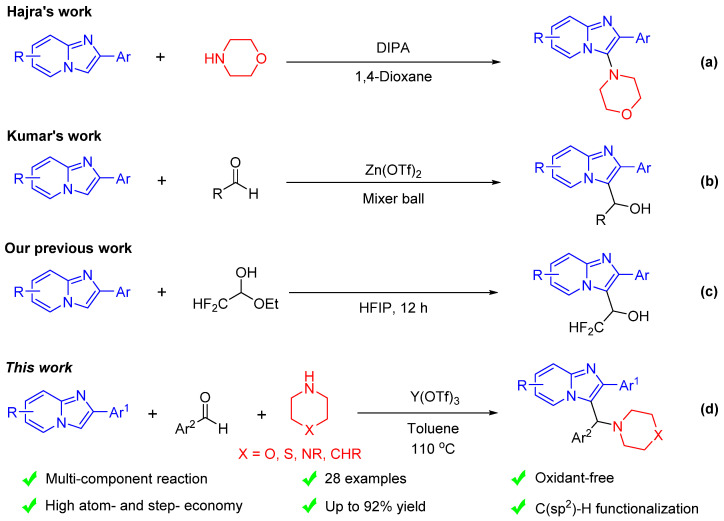
Examples of C_3_ functionalization of imidazo[1,2-a]pyridines [25,30,31].

Recently, due to the more straightforward and atom economical synthetic step, the strategy of C-H bond functionalization is believed to be an ideal approach for preparing various imidazo[1,2-a]pyridines [18]. Among them, organic peroxides [19], inorganic oxidants [20], photo/electro-induction [21,22,23], and transition metal catalysts [24] triggering C-H bond functionalization are the commonly employed strategies. For example, Hajra’s group reported a (diacetoxy)iodobenzene (DIPA)-mediated oxidative C-H amination of imidazo[1,2-a]pyridines with morpholine (Scheme 1a) [25,26].

On the other hand, the Friedel–Crafts reaction catalyzed by Lewis or Brønsted acid is another powerful strategy for the derivatization of imidazo[1,2-a]pyridines [27,28], and aldehydes and hemiacetals have been extensively used as electrophiles in these Friedel–Crafts reactions of imidazo[1,2-a]pyridines [29]. For example, Kumar’s group reported a Zn(OTf)_2_-catalyzed Friedel–Crafts hydroxyalkylation of imidazo[1,2-a]pyridines with aldehydes under mechanochemical conditions (Scheme 1b) [30]. Recently, our studies have shown that hydroxydifluoromethylation of imidazo[1,2-a]pyridines can be achieved by using 1,1,1,3,3,3-hexafluoro-2-propanol (HFIP) as the solvent and promoter (Scheme 1c) [31]. Although the research on the derivatization reaction of imidazo[1,2-a]pyridines is becoming increasingly mature, further exploration of the introduction of functional structures is needed to lay the foundation for drug development and application.

On the basis of our interest of the synthesis methodology of fused heterocycles [32,33,34], especially the synthesis and derivation of imidazo[1,2-a]pyridines [29,31], as well as the aforementioned strategies of Lewis or Brønsted acid-catalyzed Friedel–Crafts reactions, herein, we hope to disclose a facile, efficient, and Y(OTf)_3_-catalyzed method for the synthesis of C_3_-alkylated imidazo[1,2-a]pyridines through the three-component aza-Friedel–Crafts reaction of imidazo[1,2-a]pyridines, aldehydes, and amines (Scheme 1d). The advantages of this synthetic protocol include simple operation, atomic economy, oxidant-free, and a wide range of substrates. More importantly, this newly developed strategy will be helpful for further design and rapid synthesis of imidazo[1,2-a]pyridines with potential biological activity.

## 2. Results and Discussion

### 2.1. Optimization of Reaction Conditions

In recent years, aza-Friedel–Crafts-type reactions are becoming a common strategy for implementing imidazo[1,2-a]pyridines linking potentially biologically active units. For example, Kumar’s group developed a Yb(OTf)_3_-catalyzed three-component reaction of imidazo[1,2-a]pyridines, aldehydes, and acetamide [35]. On the other hand, Hao and Zhu’s group reported a MesCOOH-catalyzed arylation of imidazo[1,2-a]pyridines with aliphatic *N*-fluorosulfonamides [36].

Taking inspiration from the above literature, it is a common strategy to screen different Lewis acids and Brønsted acids to catalyze the aza-Friedel–Crafts reactions of imidazo[1,2-a]pyridines. Thus, our studies began by evaluating the reaction of 2-phenylimidazo[1,2-a]pyridine **1a**, *p*-tolualdehyde **2a**, and morpholine as the model substrates. The results are summarized in Table 1.

Firstly, the reaction of **1a** (0.2 mmol), **2a** (0.3 mmol), and **3a** (0.4 mmol) was carried out in toluene (1.0 mL) under air atmosphere at 110 °C for 12 h and the target product **4a** was not obtained without any catalyst (entry 1). If 20 mol% Brønsted acid trifluoroacetic acid (TFA), *p*-toluenesulfonic acid (TsOH), or HFIP was added as a catalyst, only trace amounts of product **4a** were produced (entries 2–4). Fortunately, when 20 mol% Lewis acid Sc(OTf)_3_ was added as a catalyst, **4a** was successfully obtained with a yield of 75% (entry 5).

It is worth noting that significant improvement in the yield of **4a** was observed by adding 20 mol% Lewis acid Y(OTf)_3_ (90%, entry 6). When the amount of Y(OTf)_3_ was decreased to 10 mol% at 110 °C in toluene, **4a** was formed in 72% yield (entry 7). Increasing the amount of Y(OTf)_3_ did not improve the yield of **4a** (entry 8).

Subsequently, several other solvents, such as *N*,*N*-dimethylformamide (DMF), 1,4-dioxane, and acetonitrile, were found to have poor efficiency (entry 6 vs. entries 9–11). Due to incomplete reaction of raw material **1a**, a significant reduction in the yield of **4a** was observed by decreasing the reaction temperature to 100 °C (62%, entry 12). Unfortunately, increasing the reaction temperature to 120 °C did not improve the yield of **4a** (entry 13). In addition, the feed ratio of reactants was discussed. It can be found that when the feed ratio of **1a**, **2a**, and **3a** was 1:1.5:2, the yield of **4a** was the highest, with a value of 90% (entry 6 vs. entries 14–16).

Thus, the optimized reaction conditions were identified as **1a** (0.2 mmol), **2a** (0.3 mmol), **3a** (0.4 mmol), and 20 mol% Y(OTf)_3_ as a catalyst and 1.0 mL of toluene as the solvent at 110 °C for 12 h.

### 2.2. Scope of Benzaldehyde Substrates ***2***

**Scheme 2:**
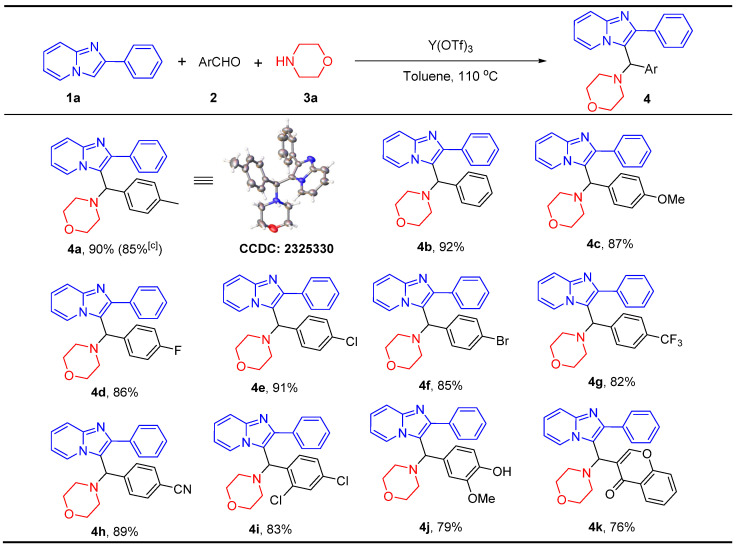
Substrate scope of various aromatic aldehydes **2** ^[a, b]^. ^[a]^ Reaction conditions: **1a** (0.2 mmol), **2** (0.3 mmol, 1.5 equiv.), **3a** (0.4 mmol, 2 equiv.), and toluene (1.0 mL), 110 °C, 12 h. ^[b]^ Isolated yields based on **1a**. ^[c]^ Reaction performed on a 6 mmol scale: **1a** (6 mmol), **2a** (9 mmol, 1.5 equiv.), **3a** (12 mmol, 2 equiv.), and toluene (20 mL), 110 °C, 24 h.

With the optimized conditions in hand, we explored the generality of the developed methodology against a variety of aromatic aldehydes (Scheme 2).

The results showed that benzaldehyde **2b**, instead of *p*-tolualdehyde **2a**, could react smoothly with 2-phenylimidazo[1,2-a]pyridine **1a** and morpholine **3a**, resulting in the corresponding product **4b** with a yield of 92%. Subsequently, under standard conditions, a series of benzaldehydes containing various substituents (such as methoxy, fluorine, chlorine, and bromine) exhibited good functional group tolerance in this method. For example, 4-methoxybenzaldehyde can react smoothly and obtain the corresponding product **4c** in 87% yield.

The 4-halogenated benzaldehydes showed good activity in this conversion, yielding different products, **4d**, **4e**, and **4f**, respectively, from *p*-fluoro, *p*-chloro, and *p*-bromo benzaldehydes in 85–91% yields. As some typical aromatic aldehyde substrates **2** containing electron-withdrawing groups, 4-trifluoromethylbenzaldehyde and 4-cyano-benzaldehyde can also be smoothly converted, affording products **4g** and **4h** in 82% and 89% yields, respectively.

As expected, multi-substituted benzaldehydes, such as 2,4-dichlorobenzaldehyde and vanillin, could provide the desired products **4i** and **4j** with yields of 83% and 79%, respectively. Captivatingly, specially functionalized aldehydes, e.g., chromone-3-carboxaldehyde **2k**, also reacted to furnish the corresponding product **4k** in 76% yield.

More importantly, to demonstrate the efficiency and practical applicability of the present approach, a gram-scale experiment was performed in the laboratory. The gram-scale reaction can be readily carried out on a 6 mmol scale (30 times), producing **4a** with a yield of 85% (1.9554 g).

### 2.3. Scope of Imidazo[1,2-a]pyridine Substrates ***1***

**Scheme 3:**
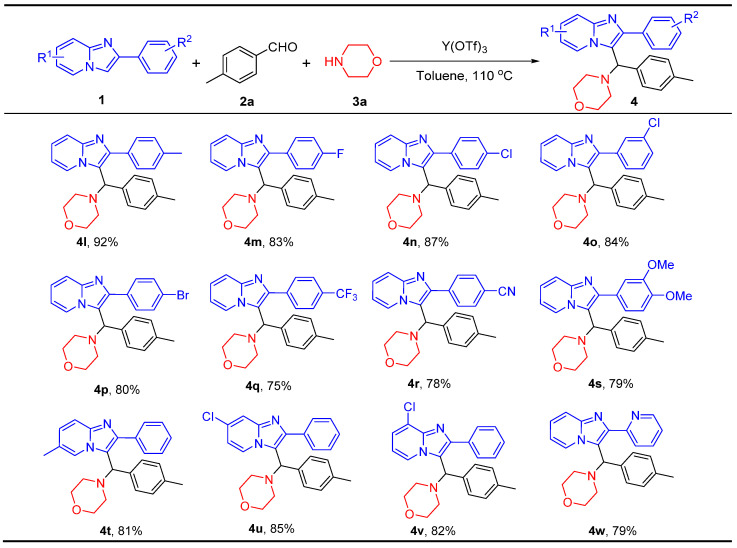
Substrate scope of various 2-aryl imidazo[1,2-a]pyridines **1** ^[a, b]^. ^[a]^ Reaction conditions: **1** (0.2 mmol), **2a** (0.3 mmol, 1.5 equiv.), **3a** (0.4 mmol, 2 equiv.), and toluene (1.0 mL), 110 °C, 12 h. ^[b]^ Isolated yields based on **1**.

Next, the substrate scope of imidazo[1,2-a]pyridines **1** was further evaluated. As shown in Scheme 3, 2-phenyl imidazo[1,2-a]pyridines **1** bearing an electron-donating group (methyl) as well as a halogen group (fluoro, chloro, and bromo) on the C2 benzene rings proceeded smoothly to yield the desired products **4l**–**4p** in moderate to good yields (80–92%).

Also, the 2-phenyl imidazo[1,2-a]pyridines **1** bearing electron-withdrawing groups, such as trifluoromethyl and cyano, on the C2 benzene rings, all reacted well to provide products **4q** and **4r** in 75% and 78% yields, respectively. As expected, this conversion was also applicable to 2-(3,4-dimethoxyphenyl)imidazo[1,2-a]pyridine **1j** as a kind of disubstituted 2-phenylimidazo[1,2-a]pyridine on the C2 benzene ring, yielding the corresponding product **4s** in 79% yield.

Moreover, a range of 2-phenyl imidazo[1,2-a]pyridines **1** bearing either electron-donating (methyl) or halogen (chloro) on the pyridine ring exhibited good functional group tolerance under the standard reaction conditions, while smoothly furnishing the corresponding alkylation products (**4t**–**4v**) in good yields (81–85%).

Similarly, 2-(pyridin-2-yl)imidazo[1,2-a]pyridine **1n** could also obtain the corresponding product **4w** in 79% yield.

### 2.4. Scope of Cycloamine Substrates ***3***

As is known, many cycloamine units, such as thiomorpholine, piperazine, and piperidine, can be potential bioactive units [37,38,39]. To further extend the scope of this reaction, we explored the possibility of using different cycloamines **3** under the optimal conditions.

**Scheme 4:**
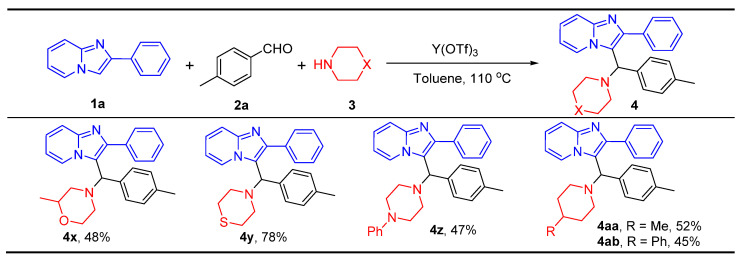
Substrate scope of various cycloamines **3** ^[a, b]^. ^[a]^ Reaction conditions: **1** (0.2 mmol), **2a** (0.3 mmol, 1.5 equiv.), **3a** (0.4 mmol, 2 equiv.), and toluene (1.0 mL), 110 °C, 12 h. ^[b]^ Isolated yields based on **1a**.

As shown in Scheme 4, the reaction of 2-methylmorpholine **3b** instead of morpholine **3a** with 2-phenylimidazo[1,2-a]pyridine **1a** and *p*-tolualdehyde **2a** under the optimized reaction conditions provided the desired compound **4x** in 48% yield.

Interestingly, thiomorpholine **3c** has excellent substrate applicability in this conversion, affording the desired product **4y** in 78% yield.

Moreover, for other cycloamines **3**, e.g., 4-phenylpiperazine **3d**, 4-methylpiperazine **3e**, and 4-phenylpiperazine **3f**, the reaction also proceeded well and yielded products **4z**–**4ab** in yields of 45–52%.

Furthermore, the structures of all expected products **4a**–**4ab** were systematically confirmed by NMR (^1^H, ^13^C, and ^19^F) and HRMS data. Especially, the structure of **4a** (CCDC 2325330) was unambiguously confirmed by single-crystal X-ray analysis, which fully proved the structure of the anticipated product (please see the Supplementary Materials for details) [40]. Thus, the structures of these serial compounds **4a**–**4ab** were well characterized, as anticipated.

### 2.5. Mechanism Investigation

**Scheme 5:**
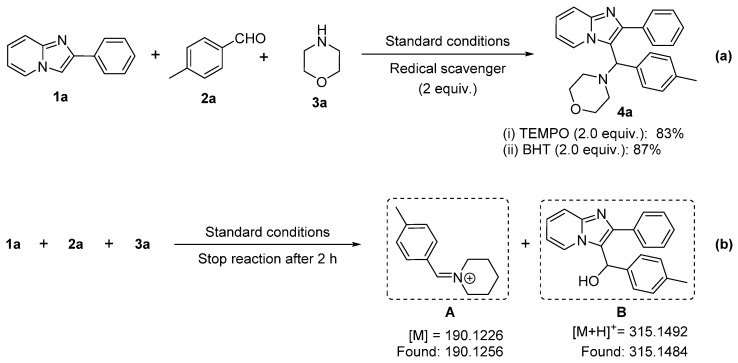
Control experiments.

In order to understand the reaction mechanism, several control experiments were carried out (Scheme 5).

Obviously, using the 2.0 equivalent free radical inhibitor TEMPO (2,2,6,6-tetra-methylpiperidine-1-oxyl) or BHT (butylated hydroxytoluene) under the standard conditions, the reaction could still obtain the desired product **3a** in 83% and 87% yields, respectively (Scheme 5a). These results demonstrated that the free radical pathway should be excluded in this transformation process.

Furthermore, as shown in Scheme 5b, to support the mechanism, we completed another control experiment and identified molecular ion peaks that correspond to intermediates iminium ion **A** and benzyl alcohol **B** by ESI-HRMS (see Supplementary Materials Figures S2 and S3 for details).

**Scheme 6:**
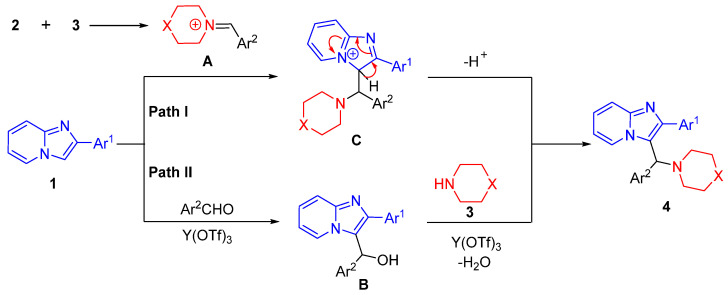
Proposed mechanisms.

On the basis of the above experimental facts and some previous related reports [41,42,43,44,45,46], as shown in Scheme 6, the plausible mechanisms were proposed for this C_3_-alkylation of imidazo[1,2-a]pyridines.

Firstly, the aldehyde **2** and cyclic amine **3** formed iminium ion **A** under the catalysis of Lewis acid. Then, the C3 position of imidazo[1,2-a]pyridine was attacked by intermediate **A** to form intermediate **C**, whose elimination of a proton yielded the desired product **4** (**Path I**) [41,42,43].

In another possible pathway, imidazo[1,2-a]pyridine **1** and aldehyde **2** underwent an electrophilic addition reaction under the action of Lewis acid to generate benzyl alcohol **B** [45,46]. The intermediate **B** reacted with cyclic amine **3**, and then there was a dehydration to produce the corresponding product **4** (**Path II**) [45].

## 3. Materials and Methods

### 3.1. General Information

Melting point (m.p.) was performed on a Büchi Melting Point B-545 instrument (Büchi, Flawil, Switzerland) without correcting. ^1^H, ^13^C, and ^19^F NMR spectra were collected on a BRUKER DRX-400 spectrometer (Bruker, Ettlingen, Germany) using tetramethylsilane (TMS) as an internal standard. High-resolution mass spectra (HRMS) were obtained with a LCMS-IT-TOF mass spectrometer. Single-crystal X-ray analysis was obtained using a Bruker APEX2 Smart CCD (Bruker, Ettlingen, Germany). TLC was performed on commercially prepared 100–400 mesh GF254 silica gel plates (Chengyang Ocean Chemical Co., Ltd., Qingdao, China) and visualization was detected at 254 or 365 nm.

All reagents and solvents were purchased from commercial sources and used without further purification, while 2-substituted imidazo[1,2-a]pyridines **1** were synthesized from 2-bromoacetophenones and various 2-aminopyridines [47,48].

### 3.2. Experimental Procedure for Compounds ***1a**–**1n***

**Scheme 7:**
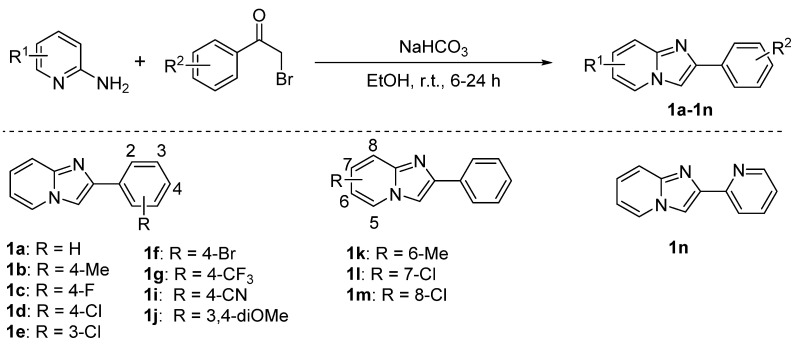
Synthesis route of **1a**–**1n**.

Compounds **1a**–**1n** were synthesized according to the reported procedure (Scheme 7) [47,48]. NaHCO_3_ (24 mmol, 1.2 equiv.) was added to the ethanol solution containing 2-bromoacetophenones (20 mmol, 1.0 equiv.) and 2-aminopyridines (22 mmol, 1.1 equiv.). Then, the reaction mixture was stirred at room temperature for 6–24 h. After the completion of the reaction, the resulting mixture was diluted with water (15 mL) and extracted with ether (3 × 20 mL). The combined organic layer was washed with brine (25 mL) and dried with anhydrous MgSO_4_, then concentrated under vacuum. The analytically pure 2-arylimidazo[1,2-a]pyridines **1a**–**1n** were obtained by silica gel column with petroleum ether/EtOAc as the eluent, with 50–90% yields.

### 3.3. Experimental Procedure for Compounds ***4a**–**4ab***

A mixture of imidazo[1,2-a]pyridine **1** (0.2 mmol, 1.0 equiv.), aromatic aldehyde **2** (0.3 mmol, 1.5 equiv.), cycloamine **3** (0.4 mmol, 2 equiv.), and Y(OTf)_3_ (0.04 mmol, 0.2 equiv.) in toluene (1.0 mL) was stirred at 110 °C for 12 h. After the completion of the reaction, the reaction mixture was quenched with H_2_O (15 mL) and extracted with ethyl acetate (3 × 15 mL). Then, the organic layer was dried over anhydrous Na_2_SO_4_. After filtration and evaporation of the solvents under reduced pressure, the crude product was purified by column chromatography on silica gel to afford the desired product **4**.

### 3.4. Characterization Data for All Products ***4a**–**4ab***

The structures of the serial compounds **4a**–**4ab** were systematically characterized via NMR, HRMS, etc., and the corresponding data are summarized in the following.

*4-((2-Phenylimidazo[1,2-a]pyridin-3-yl)(p-tolyl)methyl)morpholine* (**4a**), white solid (69 mg, 90%); m.p. 211–213 °C; ^1^H NMR (400 MHz, CDCl_3_), *δ*, ppm: 2.24 (*s*, 3H), 2.32–2.35 (*m*, 2H), 2.64–2.67 (*m*, 2H), 3.70–3.79 (*m*, 4H), 5.10 (*s*, 1H), 6.78–6.81 (*m*, 1H), 7.04 (*d*, *J* = 8.0 Hz, 2H), 7.13–7.16 (*m*, 1H), 7.20 (*d*, *J* = 8.0 Hz, 2H), 7.39–7.44 (*m*, *J* = 8.0 Hz, 1H), 7.47–7.51 (*m*, 2H), 7.58 (*d*, *J* = 9.2 Hz, 1H), 7.71 (*d*, *J* = 7.2 Hz, 2H), 9.07 (*d*, *J* = 8.0 Hz, 1H); ^13^C NMR (100 MHz, CDCl_3_), *δ*, ppm: 21.02, 52.67, 66.65, 67.25, 112.04, 117.54, 119.59, 124.47, 126.30, 127.46, 127.93, 128.50, 129.30, 129.52, 134.75, 135.76, 137.25, 145.04, 145.20; ESI-HRMS, *m*/*z*: Calcd for C_25_H_26_N_3_O [M + H]^+^, 384.2070, found: 384.2067.
*4-(Phenyl(2-phenylimidazo[1,2-a]pyridin-3-yl)methyl)morpholine* (**4b**), white solid (68 mg, 92%); m.p. 188–190 °C; ^1^H NMR (400 MHz, CDCl_3_), *δ*, ppm: 2.33–2.38 (*m*, 2H), 2.62–2.72 (*m*, 2H), 3.75–3.78 (*m*, 4H), 5.15 (*s*, 1H), 6.79–6.82 (*m*, 1H), 7.16–7.24 (*m*, 4H), 7.32 (*d*, *J* = 8.0 Hz, 2H), 7.41–7.45 (*m*, 1H), 7.49–7.52 (*m*, 2H), 7.59 (*d*, *J* = 8.0 Hz, 1H), 7.71 (*d*, *J* = 8.0 Hz, 2H), 9.02 (*d*, *J* = 8.0 Hz, 1H); ^13^C NMR (100 MHz, CDCl_3_), *δ*, ppm: 52.66, 66.78, 67.24, 112.10, 117.56, 119.34, 124.53, 126.16, 127.51, 127.96, 128.52, 128.59, 129.50, 134.65, 138.69, 145.07, 145.34; ESI-HRMS, *m*/*z*: Calcd for C_24_H_24_N_3_O [M + H]^+^, 370.1914, found: 370.1929.
*4-((4-Methoxyphenyl)(2-phenylimidazo[1,2-a]pyridin-3-yl)methyl)morpholine* (**4c**), white solid (69 mg, 87%); m.p. 206–208 °C; ^1^H NMR (400 MHz, CDCl_3_), *δ*, ppm: 2.31–2.36 (*m*, 2H), 2.63–2.66 (*m*, 2H), 3.73–3.77 (*m*, 7H), 5.08 (*s*, 1H), 7.76 (*d*, *J* = 8.8 Hz, 2H), 6.80–6.84 (*m*, 1H), 7.17–7.24 (*m*, 3H), 7.41–7.44 (*m*, 1H), 7.48–7.51 (*m*, 2H), 7.61 (*d*, *J* = 8.0 Hz, 1H), 7.70 (*d*, *J* = 8.0 Hz, 2H), 9.08 (*d*, *J* = 8.0 Hz, 1H); ^13^C NMR (100 MHz, CDCl_3_), *δ*, ppm: 52.65, 55.21, 66.30, 67.25, 112.05, 113.94, 117.56, 119.72, 124.45, 126.23, 127.91, 128.47, 128.65, 129.48, 130.85, 134.65, 144.99, 145.06, 158.85; ESI-HRMS, *m*/*z*: Calcd for C_25_H_26_N_3_O_2_ [M + H]^+^, 400.2020, found: 400.2021.
*4-((4-Fluorophenyl)(2-phenylimidazo[1,2-a]pyridin-3-yl)methyl)morpholine* (**4d**), white solid (67 mg, 86%); m.p. 200–202 °C; ^1^H NMR (400 MHz, CDCl_3_), *δ*, ppm: 2.34–2.39 (*m*, 2H), 2.66–2.70 (*m*, 2H), 3.76–3.79 (*m*, 4H), 5.14 (*s*, 1H), 6.80–6.84 (*m*, 1H), 6.89–6.94 (*m*, 2H), 7.18–7.22 (*m*, 1H), 7.25–7.29 (*m*, 2H), 7.42–7.46 (*m*, 1H), 7.49–7.53 (*m*, 2H), 7.61 (*d*, *J* = 8.0 Hz, 1H), 7.70 (*d*, *J* = 8.0 Hz, 2H), 8.96 (*d*, *J* = 6.0 Hz, 1H); ^13^C NMR (100 MHz, CDCl_3_), *δ*, ppm: 52.63, 66.08, 67.21, 112.31, 115.51 (*d*, *J* = 22 Hz), 117.68, 119.12, 124.69, 125.89, 128.09, 128.61, 128.95 (*d*, *J* = 8 Hz), 129.48, 134.42 (*d*, *J* = 4 Hz), 134.49, 145.12, 145.37, 161.95 (*d*, *J* = 245 Hz); ^19^F NMR (376 MHz, CDCl_3_), *δ*, ppm: −114.65; ESI-HRMS, *m*/*z*: Calcd for C_24_H_23_FN_3_O [M + H]^+^, 388.1820, found: 388.1830.
*4-((4-Chlorophenyl)(2-phenylimidazo[1,2-a]pyridin-3-yl)methyl)morpholine* (**4e**), white solid (73 mg, 91%); m.p. 215–217 °C; ^1^H NMR (400 MHz, CDCl_3_), *δ*, ppm: 2.34–2.39 (*m*, 2H), 2.66–2.70 (*m*, 2H), 3.73–3.82 (*m*, 4H), 5.14 (*s*, 1H), 6.79–6.83 (*m*, 1H), 7.17–7.26 (*m*, 5H), 7.42–7.45 (*m*, 1H), 7.49–7.53 (*m*, 2H), 7.60 (*d*, *J* = 8.0 Hz, 1H), 7.70 (*d*, *J* = 8.0 Hz, 2H), 8.92 (*d*, *J* = 8.0 Hz, 1H); ^13^C NMR (100 MHz, CDCl_3_), *δ*, ppm: 52.61, 66.13, 67.17, 112.33, 117.70, 118.77, 124.69, 125.81, 128.10, 128.61, 128.67, 128.76, 129.46, 133.19, 134.48, 137.15, 145.16, 145.53; ESI-HRMS, *m*/*z*: Calcd for C_24_H_23_ClN_3_O [M+H]^+^, 404.1524, found: 404.1531.
*4-((4-Bromophenyl)(2-phenylimidazo[1,2-a]pyridin-3-yl)methyl)morpholine* (**4f**), light-yellow solid (76 mg, 85%); m.p. 193–195 °C; ^1^H NMR (400 MHz, CDCl_3_), *δ*, ppm: 2.35–2.39 (*m*, 2H), 2.67–2.70 (*m*, 2H), 3.73–3.82 (*m*, 4H), 5.13 (*s*, 1H), 6.80–6.84 (*m*, 1H), 7.16–7.22 (*m*, 3H), 7.34 (*d*, *J* = 8.0 Hz, 2H), 7.43–7.46 (*m*, 1H), 7.50–7.53 (*m*, 2H), 7.61 (*d*, *J* = 8.0 Hz, 1H), 7.69 (*d*, *J* = 8.0 Hz, 2H), 8.92 (*d*, *J* = 6.0 Hz, 1H); ^13^C NMR (100 MHz, CDCl_3_), *δ*, ppm: 52.61, 66.19, 67.17, 112.34, 117.72, 118.66, 121.30, 124.70, 125.80, 128.10, 128.62, 129.01, 129.46, 131.71, 134.48, 137.68, 145.17, 145.56; ESI-HRMS, *m*/*z*: Calcd for C_24_H_23_BrN_3_O [M+H]^+^, 448.1019, found: 448.1028.
*4-((2-Phenylimidazo[1,2-a]pyridin-3-yl)(4-(trifluoromethyl)phenyl)methyl)-morpholine* (**4g**), light-yellow solid (72 mg, 82%); m.p. 196–198 °C; ^1^H NMR (400 MHz, CDCl_3_), *δ*, ppm: 2.38–2.43 (*m*, 2H), 2.68–2.73 (*m*, 2H), 3.76–3.85 (*m*, 4H), 5.24 (*s*, 1H), 6.82–6.86 (*m*, 1H), 7.19–7.24 (*m*, 1H), 7.41 (*d*, *J* = 8.0 Hz, 2H), 7.44–7.48 (*m*, 3H), 7.51–7.55 (*m*, 2H), 7.62 (*d*, *J* = 8.0 Hz, 1H), 7.71 (*d*, *J* = 8.0 Hz, 2H), 8.92 (*d*, *J* = 6.0 Hz, 1H); ^13^C NMR (100 MHz, CDCl_3_), *δ*, ppm: 52.63, 66.40, 67.14, 112.52, 117.79, 118.31, 123.89 (*q*, *J* = 271 Hz), 124.86, 125.24 (*q*, *J* = 3 Hz), 125.58 (*q*, *J* = 4 Hz), 127.59, 128.22, 128.71, 129.48, 129.72 (*q*, *J* = 33 Hz), 134.39, 142.63, 145.22, 145.78; ^19^F NMR (376 MHz, CDCl_3_), *δ*, ppm: −62.61; ESI-HRMS, *m*/*z*: Calcd for C_25_H_23_F_3_N_3_O [M + H]^+^, 438.1788, found: 438.1762.
*4-(Morpholino(2-phenylimidazo[1,2-a]pyridin-3-yl)methyl)benzonitrile* (**4h**), light-yellow solid (70 mg, 89%); m.p. 208–210 °C; ^1^H NMR (400 MHz, CDCl_3_), *δ*, ppm: 2.42–2.44 (*m*, 2H), 2.68–2.73 (*m*, 2H), 3.77–3.85 (*m*, 4H), 5.24 (*s*, 1H), 6.82–6.85 (*m*, 1H), 7.20–7.24 (*m*, 1H), 7.39 (*d*, *J* = 8.0 Hz, 2H), 7.45–7.55 (*m*, 5H), 7.63 (*d*, *J* = 8.0 Hz, 1H), 7.70 (*d*, *J* = 8.0 Hz, 2H), 8.81 (*d*, *J* = 8.0 Hz, 1H); ^13^C NMR (100 MHz, CDCl_3_), *δ*, ppm: 52.58, 66.31, 67.09, 111.39, 112.77, 117.81, 118.44, 125.14, 125.37, 127.89, 128.37, 128.79, 129.44, 132.42, 134.07, 143.84, 145.29, 145.84; ESI-HRMS, *m*/*z*: Calcd for C_25_H_23_N_4_O [M + H]^+^, 395.1866, found: 395.1878.
*4-((2,4-Dichlorophenyl)(2-phenylimidazo[1,2-a]pyridin-3-yl)methyl)morpholine* (**4i**), light-yellow solid (72 mg, 83%); m.p. 216–218 °C; ^1^H NMR (400 MHz, CDCl_3_), *δ*, ppm: 2.46–2.51 (*m*, 2H), 2.66–2.72 (*m*, 2H), 3.73–3.80 (*m*, 4H), 5.48 (*s*, 1H), 6.82–6.85 (*m*, 1H), 7.15–7.24 (*m*, 3H), 7.37–7.47 (*m*, 3H), 7.62–7.69 (*m*, 3H), 7.77 (*d*, *J* = 8.0 Hz, 1H), 8.87 (*d*, *J* = 8.0 Hz, 1H); ^13^C NMR (100 MHz, CDCl_3_), *δ*, ppm: 52.07, 62.27, 67.24, 112.39, 116.73, 117.87, 124.61, 125.50, 126.81, 127.98, 128.26, 129.32, 129.60, 130.13, 133.75, 134.23, 134.83, 135.41, 145.24, 146.66; ESI-HRMS, *m*/*z*: Calcd for C_24_H_22_Cl_2_N_3_O [M + H]^+^, 438.1134, found: 438.1120.
*2-Methoxy-4-(morpholino(2-phenylimidazo[1,2-a]pyridin-3-yl)methyl)phenol* (**4j**), white solid (65 mg, 79%); m.p. 209–212 °C; ^1^H NMR (400 MHz, CDCl_3_), *δ*, ppm: 2.33–2.38 (*m*, 2H), 2.64–2.72 (*m*, 2H), 3.66 (*s*, 3H), 3.75–3.80 (*m*, 4H), 6.60 (*s*, 1H), 6.77 (*d*, *J* = 8.0 Hz, 1H), 6.80–6.83 (*m*, 1H), 6.91–6.94 (*m*, 1H), 7.16–7.19 (*m*, 1H), 7.40–7.44 (*m*, 1H), 7.47–7.51 (*m*, 2H), 7.60 (*d*, *J* = 8.0 Hz, 1H), 7.69 (*d*, *J* = 8.0 Hz, 2H), 9.06 (*d*, *J* = 6.0 Hz, 1H); ^13^C NMR (100 MHz, CDCl_3_), *δ*, ppm: 52.69, 55.64, 66.78, 67.24, 110.17, 112.08, 114.21, 117.59, 119.68, 119.81, 124.48, 126.12, 127.96, 128.47, 129.55, 130.66, 134.74, 144.97, 145.01, 145.07, 146.60; ESI-HRMS, *m*/*z*: Calcd for C_25_H_26_N_3_O_3_ [M + H]^+^, 416.1969, found: 416.1960.
*3-(Morpholino(2-phenylimidazo[1,2-a]pyridin-3-yl)methyl)-4H-chromen-4-one* (**4k**), yellow solid (66 mg, 76%); m.p. 112–114 °C; 2.45–2.50 (*m*, 2H), 2.62–2.74 (*m*, 2H), 3.71–3.73 (*m*, 4H), 5.48 (*s*, 1H), 6.89–6.92 (*m*, 1H), 721–7.25 (*m*, 1H), 7.33–7.47 (*m*, 5H), 7.60–7.66 (*m*, 2H), 7.76 (*d*, *J* = 8.0 Hz, 2H), 8.05 (*s*, 1H), 8.11 (*d*, *J* = 8.0 Hz, 1H), 8.90 (*d*, *J* = 8.0 Hz, 1H); ^13^C NMR (100 MHz, CDCl_3_), *δ*, ppm: 52.12, 56.87, 67.12, 112.37, 116.88, 117.78, 117.98, 120.92, 123.62, 124.44, 125.29, 125.38, 126.03, 128.03, 128.17, 129.43, 133.74, 134.59; 145.08, 145.97, 154.11, 155.92, 176.59; ESI-HRMS, *m*/*z*: Calcd for C_27_H_24_N_3_O_3_ [M + H]^+^, 438.1812, found: 438.1805.
*4-(p-Tolyl(2-(p-tolyl)imidazo[1,2-a]pyridin-3-yl)methyl)morpholine* (**4l**), white solid (73 mg, 92%); m.p. 205–207 °C; ^1^H NMR (400 MHz, CDCl_3_), *δ*, ppm: 2.15 (*s*, 3H), 2.22–2.27 (*m*, 2H), 2.35 (*s*, 3H), 2.55–2.58 (*m*, 2H), 3.62–3.70 (*m*, 4H), 5.02 (*s*, 1H), 6.67–6.71 (*m*, 1H), 6.93 (*d*, *J* = 8.0 Hz, 2H), 7.03–7.07 (*m*, 1H), 7.12 (*d*, *J* = 8.4 Hz, 2H), 7.21 (*d*, *J* = 8.4 Hz, 2H), 7.47–7.53 (*m*, 3H), 8.96 (*d*, *J* = 6.8 Hz, 1H); ^13^C NMR (100 MHz, CDCl_3_), *δ*, ppm: 21.01, 21.38, 52.66, 66.62, 67.25, 111.91, 117.44, 119.37, 124.32, 126.22, 127.47, 129.21, 129.25, 129.35, 131.79, 135.80, 137.16, 137.63, 144.97, 145.26; ESI-HRMS, *m*/*z*: Calcd for C_26_H_28_N_3_O [M + H]^+^, 398.2227, found: 398.2236.
*4-((2-(4-Fluorophenyl)imidazo[1,2-a]pyridin-3-yl)(p-tolyl)methyl)morpholine* (**4m**), white solid (67 mg, 83%); m.p. 182–184 °C; ^1^H NMR (400 MHz, CDCl_3_), *δ*, ppm: 2.26 (*s*, 3H), 2.30–2.34 (*m*, 2H), 2.61–2.64 (*m*, 2H), 3.71–3.78 (*m*, 4H), 5.01 (*s*, 1H), 6.80–6.83 (*m*, 1H), 7.04 (*d*, *J* = 7.6 Hz, 2H), 7.16–7.21 (*m*, 5H), 7.57 (*d*, *J* = 8.0 Hz, 1H), 7.65–7.68 (*m*, 2H), 9.09 (*d*, *J* = 6.0 Hz, 1H); ^13^C NMR (100 MHz, CDCl_3_), *δ*, ppm: 21.00, 52.68, 66.83, 67.19, 112.11, 115.48 (*d*, *J* = 21 Hz), 117.51, 119.54, 124.57, 126.28, 127.42, 129.34, 130.82 (*d*, *J* = 3 Hz), 131.16 (*d*, *J* = 8 Hz), 135.67, 137.40, 144.21, 145.00, 162.66 (*d*, *J* = 245 Hz); ^19^F NMR (376 MHz, CDCl_3_), *δ*, ppm: −114.10; ESI-HRMS, *m*/*z*: Calcd for C_25_H_25_FN_3_O [M + H]^+^, 402.1976, found: 402.1987.
*4-((2-(4-Chlorophenyl)imidazo[1,2-a]pyridin-3-yl)(p-tolyl)methyl)morpholine* (**4n**), light-yellow solid (72 mg, 87%); m.p. 173–175 °C; ^1^H NMR (400 MHz, CDCl_3_), *δ*, ppm: 2.24 (*s*, 3H), 2.28–2.40 (*m*, 2H), 2.61–2.63 (*m*, 2H), 3.65–3.77 (*m*, 4H), 5.03 (*s*, 1H), 6.80–6.83 (*m*, 1H), 7.03 (*d*, *J* = 7.6 Hz, 2H), 7.14–7.20 (*m*, 3H), 7.46 (*d*, *J* = 6.8 Hz, 2H), 7.57 (*d*, *J* = 8.8 Hz, 1H), 7.65 (*d*, *J* = 6.8 Hz, 2H), 9.10 (*d*, *J* = 6.0 Hz, 1H); ^13^C NMR (100 MHz, CDCl_3_), *δ*, ppm: 21.02, 52.70, 66.87, 67.18, 112.20, 117.56, 119.79, 124.71, 126.38, 127.45, 128.73, 129.39, 130.74, 133.29, 133.95, 135.61, 137.45, 143.93, 145.11; ESI-HRMS, *m*/*z*: Calcd for C_25_H_25_ClN_3_O [M + H]^+^, 418.1681, found: 418.1662.
*4-((2-(3-Chlorophenyl)imidazo[1,2-a]pyridin-3-yl)(p-tolyl)methyl)morpholine* (**4o**), light-yellow solid (70 mg, 84%); m.p. 129–131 °C; ^1^H NMR (400 MHz, CDCl_3_), *δ*, ppm: 2.19 (*s*, 3H), 2.23–2.28 (*m*, 2H), 2.53–2.56 (*m*, 2H), 3.63–3.71 (*m*, 4H), 4.96 (*s*, 1H), 6.74–6.78 (*m*, 1H), 6.98 (*d*, *J* = 8.0 Hz, 2H), 7.10–7.15 (*m*, 3H), 7.31–7.37 (*m*, 2H), 7.50–7.53 (*m*, 2H), 7.65 (*s*, 1H), 9.05 (*d*, *J* = 6.0 Hz, 1H); ^13^C NMR (100 MHz, CDCl_3_), *δ*, ppm: 21.02, 52.68, 66.86, 67.19, 112.25, 117.65, 120.00, 124.76, 126.41, 127.50, 127.55, 128.00, 129.42, 129.55, 129.71, 134.39, 135.58, 136.57, 137.53, 143.65, 145.12; ESI-HRMS, *m*/*z*: Calcd for C_25_H_25_ClN_3_O [M + H]^+^, 418.1681, found: 418.1662.
*4-((2-(4-Bromophenyl)imidazo[1,2-a]pyridin-3-yl)(p-tolyl)methyl)morpholine* (**4p**), light-yellow solid (73 mg, 80%); m.p. 224–226 °C; ^1^H NMR (400 MHz, CDCl_3_), *δ*, ppm: 2.26 (*s*, 3H), 2.26–2.34 (*m*, 2H), 2.60–2.63 (*m*, 2H), 3.71–3.79 (*m*, 4H), 5.01 (*s*, 1H), 6.80–6.84 (*m*, 1H), 7.04 (*d*, *J* = 8.0 Hz, 2H), 7.17–7.21 (*m*, 3H), 7.57–7.64 (*m*, 5H), 9.10 (*d*, *J* = 8.0 Hz, 1H); ^13^C NMR (100 MHz, CDCl_3_), *δ*, ppm: 21.02, 52.70, 66.86, 67.19, 112.21, 117.57, 119.79, 122.23, 124.74, 126.36, 127.45, 129.39, 131.03, 131.68, 133.69, 135.58, 137.48, 143.92, 145.12; ESI-HRMS, *m*/*z*: Calcd for C_25_H_25_BrN_3_O [M + H]^+^, 462.1176, found: 462.1188.
*4-(p-Tolyl(2-(4-(trifluoromethyl)phenyl)imidazo[1,2-a]pyridin-3-yl)methyl)-morpholine* (**4q**), light-yellow solid (67 mg, 75%); m.p. 161–163 °C; ^1^H NMR (400 MHz, CDCl_3_), *δ*, ppm: 2.26 (*s*, 3H), 2.32–2.36 (*m*, 2H), 2.61–2.64 (*m*, 2H), 3.73–3.76 (*m*, 4H), 5.04 (*s*, 1H), 6.83–6.86 (*m*, 1H), 7.05 (*d*, *J* = 8.0 Hz, 2H), 7.18–7.23 (*m*, 3H), 7.60 (*d*, *J* = 8.0 Hz, 1H), 7.75 (*d*, *J* = 8.0 Hz, 2H), 7.84 (*d*, *J* = 8.0 Hz, 2H), 9.15 (*d*, *J* = 7.2 Hz, 1H); ^13^C NMR (100 MHz, CDCl_3_), *δ*, ppm: 21.01, 52.71, 66.91, 67.16, 112.35, 117.71, 120.31, 124.27 (*q*, *J* = 271 Hz), 124.92, 125.42 (*q*, *J* = 3 Hz), 126.49, 127.48, 129.43, 129.67 (*q*, *J* = 24 Hz), 135.48, 137.59, 138.42, 143.55, 145.24; ^19^F NMR (376 MHz, CDCl_3_), *δ*, ppm: −62.39; ESI-HRMS, *m*/*z*: Calcd for C_26_H_25_F_3_N_3_O [M + H]^+^, 452.1944, found: 452.1965.
*4-(3-(Morpholino(p-tolyl)methyl)imidazo[1,2-a]pyridin-2-yl)benzonitrile* (**4r**), light-yellow solid (63 mg, 78%); m.p. 198–200 °C; ^1^H NMR (400 MHz, CDCl_3_), *δ*, ppm: 2.27 (*s*, 3H), 2.30–2.35 (*m*, 2H), 2.59–2.62 (*m*, 2H), 3.73–3.78 (*m*, 4H), 5.01 (*s*, 1H), 6.85–6.88 (*m*, 1H), 7.06 (*d*, *J* = 8.0 Hz, 2H), 7.17 (*d*, *J* = 8.0 Hz, 2H), 7.21–7.25 (*m*, 1H), 7.60 (*d*, *J* = 9.2 Hz, 1H), 7.78 (*d*, *J* = 8.0 Hz, 2H), 7.85 (*d*, *J* = 8.0 Hz, 2H), 9.16 (*d*, *J* = 8.0 Hz, 1H); ^13^C NMR (100 MHz, CDCl_3_), *δ*, ppm: 17.08, 48.79, 63.10, 63.17, 107.52, 108.64, 113.81, 114.98, 116.75, 121.29, 122.62, 123.51, 125.58, 125.99, 128.36, 131.34, 133.84, 135.50, 138.93, 141.41; ESI-HRMS, *m*/*z*: Calcd for C_26_H_25_N_4_O [M + H]^+^, 409.2023, found: 409.2034.
*4-((2-(3,4-Dimethoxyphenyl)imidazo[1,2-a]pyridin-3-yl)(p-tolyl)methyl)morpholine* (**4s**), light-yellow solid (70 mg, 79%); m.p. 194–196 °C; ^1^H NMR (400 MHz, CDCl_3_), *δ*, ppm: 2.25 (*s*, 3H), 2.31–2.32 (*m*, 2H), 2.62–2.66 (*m*, 2H), 3.71–3.78 (*m*, 4H), 3.95 (*s*, 3H), 3.97 (*s*, 3H), 5.10 (*s*, 1H), 6.79–6.83 (*m*, 1H), 6.99 (*d*, *J* = 8.0 Hz, 1H), 7.03 (*d*, *J* = 8.0 Hz, 2H), 7.15–7.24 (*m*, 5H), 7.59 (*d*, *J* = 8.0 Hz, 1H), 9.09 (*d*, *J* = 8.0 Hz, 1H); ^13^C NMR (100 MHz, CDCl_3_), *δ*, ppm: 21.00, 52.75, 55.93, 56.03, 66.77, 67.24, 110.94, 112.00, 112.66, 117.33, 119.27, 121.81, 124.47, 126.26, 127.32, 127.51, 129.26, 135.79, 137.31, 144.82, 144.95, 148.91; ESI-HRMS, *m*/*z*: Calcd for C_27_H_30_N_3_O_3_ [M + H]^+^, 444.2282, found: 444.2268.
*4-((6-Methyl-2-phenylimidazo[1,2-a]pyridin-3-yl)(p-tolyl)methyl)morpholine* (**4t**), light-yellow solid (64 mg, 81%); m.p. 195–197 °C; ^1^H NMR (400 MHz, CDCl_3_), *δ*, ppm: 2.17 (*s*, 3H), 2.22–2.25 (*m*, 2H), 2.28 (*s*, 3H), 2.54–2.61 (*m*, 2H), 3.65–3.71 (*m*, 4H), 4.98 (*s*, 1H), 6.92–6.97 (*m*, 3H), 7.12 (*d*, *J* = 8.0 Hz, 2H), 7.30–7.34 (*m*, 1H), 7.38–7.42 (*m*, 3H), 7.61 (*d*, *J* = 8.0 Hz, 2H), 7.86 (*s*, 1H); ^13^C NMR (100 MHz, CDCl_3_), *δ*, ppm: 18.69, 21.02, 52.64, 66.60, 67.30, 116.77, 119.25, 121.55, 123.78, 127.52, 127.60, 127.79, 128.42, 129.24, 129.48, 134.83, 135.85, 137.16, 144.09, 144.96; ESI-HRMS, *m*/*z*: Calcd for C_26_H_28_N_3_O [M + H]^+^, 398.2227, found: 398.2236.
*4-((7-Chloro-2-phenylimidazo[1,2-a]pyridin-3-yl)(p-tolyl)methyl)morpholine* (**4u**), white solid (71 mg, 85%); m.p. 197–199 °C; ^1^H NMR (400 MHz, CDCl_3_), *δ*, ppm: 2.26 (*s*, 3H), 2.31–2.35 (*m*, 2H), 2.63–2.71 (*m*, 2H), 3.73–3.80 (*m*, 4H), 5.06 (*s*, 1H), 6.80–6.82 (*m*, 1H), 7.04 (*d*, *J* = 8.0 Hz, 2H), 7.16 (*d*, *J* = 8.0 Hz, 2H), 7.42–7.45 (*m*, 1H), 7.48–7.52 (*m*, 2H), 7.58–7.67 (*m*, 3H), 9.06 (*d*, *J* = 8.0 Hz, 1H); ^13^C NMR (100 MHz, CDCl_3_), *δ*, ppm: 20.96, 52.61, 66.52, 67.14, 113.84, 116.07, 120.01, 126.61, 127.33, 128.27, 128.57, 129.18, 129.36, 129.41, 129.57, 133.84, 135.28, 137.56, 144.75; ESI-HRMS, *m*/*z*: Calcd for C_25_H_25_ClN_3_O [M + H]^+^, 418.1681, found: 418.1662.
*4-((8-Chloro-2-phenylimidazo[1,2-a]pyridin-3-yl)(p-tolyl)methyl)morpholine* (**4v**), white solid (68 mg, 82%); m.p. 189–191 °C; ^1^H NMR (400 MHz, CDCl_3_), *δ*, ppm: 2.25 (*s*, 3H), 2.30–2.35 (*m*, 2H), 2.64–2.67 (*m*, 2H), 3.70–3.79 (*m*, 4H), 5.06 (*s*, 1H), 6.73–6.77 (*m*, 1H), 7.03 (*d*, *J* = 8.0 Hz, 2H), 7.17 (*d*, *J* = 8.0 Hz, 2H), 7.25 (*d*, *J* = 8.0 Hz, 1H), 7.41–7.51 (*m*, 3H), 7.69 (*d*, *J* = 8.0 Hz, 2H), 9.05 (*d*, *J* = 8.0 Hz, 1H); ^13^C NMR (100 MHz, CDCl_3_), *δ*, ppm: 21.01, 52.65, 66.67, 67.18, 111.62, 121.44, 123.16, 123.42, 125.04, 127.38, 128.16, 128.48, 129.35, 129.78, 134.21, 135.36, 137.44, 142.38, 145.91; ESI-HRMS, *m*/*z*: Calcd for C_25_H_25_ClN_3_O [M + H]^+^, 418.1681, found: 418.1662.
*4-((2-(Pyridin-2-yl)imidazo[1,2-a]pyridin-3-yl)(p-tolyl)methyl)morpholine* (**4w**), white solid (60 mg, 79%); m.p. 201–203 °C; ^1^H NMR (400 MHz, CDCl_3_), *δ*, ppm: 2.25 (*s*, 3H), 2.28–2.33 (*m*, 2H), 2.71–2.74 (*m*, 2H), 3.74–3.79 (*m*, 4H), 6.66 (*s*, 1H), 6.76–6.79 (*m*, 1H), 7.04 (*d*, *J* = 8.0 Hz, 2H), 7.13–7.17 (*m*, 1H), 7.23–7.24 (*m*, 1H), 7.55–7.60 (*m*, 3H), 7.76–7.80 (*m*, 1H), 8.25 (*d*, *J* = 8.0 Hz, 1H), 8.72 (*d*, *J* = 8.0 Hz, 1H), 9.14 (*d*, *J* = 6.0 Hz, 1H); ^13^C NMR (100 MHz, CDCl_3_), *δ*, ppm: 21.04, 52.52, 64.47, 67.29, 112.05, 117.59, 122.13, 122.55, 122.62, 124.68, 126.92, 127.70, 129.06, 136.37, 136.48, 136.73, 141.76, 144.68, 148.61, 154.61; ESI-HRMS, *m*/*z*: Calcd for C_24_H_25_N_4_O [M + H]^+^, 385.2033, found: 385.2059.
*2-Methyl-4-((2-phenylimidazo[1,2-a]pyridin-3-yl)(p-tolyl)methyl)morpholine* (**4x**), white solid (38 mg, 48%); m.p. 182–184 °C; ^1^H NMR (400 MHz, CDCl_3_), *δ*, ppm: 1.12 (*d*, *J* = 8.0 Hz, 3H), 1.84–1.87 (*m*, 1H), 2.26 (*s*, 3H), 2.40–2.44 (*m*, 2H), 2.87 (*d*, *J* = 8.8 Hz, 1H), 3.66–3.79 (*m*, 2H), 3.82–3.86 (*m*, 1H), 5.08 (*s*, 1H), 6.78–6.81 (*m*, 1H), 7.03 (*d*, *J* = 8.0 Hz, 2H), 7.15–7.20 (*m*, 3H), 7.41–7.44 (*m*, 1H), 7.48–7.51 (*m*, 2H), 7.58 (*d*, *J* = 8.8 Hz, 1H), 7.70 (*d*, *J* = 8.0 Hz, 2H), 9.03 (*d*, *J* = 8.0 Hz, 1H); ^13^C NMR (100 MHz, CDCl_3_), *δ*, ppm: 19.18, 21.02, 51.64, 59.22, 66.39, 67.12, 71.94, 112.02, 117.50, 119.71, 124.47, 126.23, 127.39, 127.91, 128.48, 129.27, 129.52, 134.66, 135.70, 137.19, 144.98, 145.04; ESI-HRMS, *m*/*z*: Calcd for C_26_H_28_N_3_O [M + H]^+^, 398.2227, found: 398.2236.
*4-((2-phenylimidazo[1,2-a]pyridin-3-yl)(p-tolyl)methyl)thiomorpholine* (**4y**), yellow solid (42 mg, 62%); m.p. 190–192 °C; ^1^H NMR (400 MHz, CDCl_3_), *δ*, ppm: 2.23 (*s*, 3H), 2.63–2.74 (*m*, 6H), 2.89–2.94 (*m*, 2H), 5.20 (*s*, 1H), 6.75–6.79 (*m*, 1H), 7.01 (*d*, *J* = 8.0 Hz, 2H), 7.10–7.16 (*m*, 3H), 7.41–7.43 (*m*, 1H), 7.46–7.50 (*m*, 2H), 7.54 (*d*, *J* = 8.4 Hz, 1H), 7.68 (*d*, *J* = 8.0 Hz, 2H), 8.87 (*d*, *J* = 8.0 Hz, 1H); ^13^C NMR (100 MHz, CDCl_3_), *δ*, ppm: 21.00, 28.47, 53.92, 66.28, 112.02, 117.47, 119.51, 124.57, 126.20, 127.22, 127.95, 128.54, 129.27, 129.51, 134.62, 135.84, 137.12, 145.08, 145.25; ESI-HRMS, *m*/*z*: Calcd for C_25_H_26_N_3_S [M + H]^+^, 400.1842, found: 400.1857.
*2-Phenyl-3-((4-phenylpiperazin-1-yl)(p-tolyl)methyl)imidazo[1,2-a]pyridine* (**4z**), yellow solid (43 mg, 47%); m.p. 95–97 °C; 2.26 (*s*, 3H), 2.51–2.55 (*m*, 2H), 2.82–2.87 (*m*, 2H), 3.19–3.28 (*m*, 4H), 5.17 (*s*, 1H), 6.79–6.80 (*m*, 1H), 6.85–6.88 (*m*, 1H), 6.92 (*d*, *J* = 8.0 Hz, 2H), 7.04 (*d*, *J* = 8.0 Hz, 2H), 7.15–7.19 (*m*, 1H), 7.21–7.24 (*m*, 2H), 7.25–7.28 (*m*, 2H), 7.43 (*d*, *J* = 8.0 Hz, 1H), 7.48–7.51 (*m*, 2H), 7.60 (*d*, *J* = 9.2 Hz, 1H), 7.73 (*d*, *J* = 8.0 Hz, 2H), 9.06 (*d*, *J* = 7.2 Hz, 1H); ^13^C NMR (100 MHz, CDCl_3_), *δ*, ppm: 21.04, 49.53, 52.09, 66.19, 112.00, 116.02, 117.51, 119.89, 119.94, 124.51, 126.42, 127.37, 127.93, 128.52, 128.77, 129.17, 129.28, 129.57, 134.74, 136.05, 137.19, 145.06, 151.15; ESI-HRMS, *m*/*z*: Calcd for C_31_H_31_N_4_ [M + H]^+^, 459.2543, found: 459.2557.
*3-((4-Methylpiperidin-1-yl)(p-tolyl)methyl)-2-phenylimidazo[1,2-a]pyridine* (**4aa**), light-yellow solid (40 mg, 52%); m.p. 156–158 °C; ^1^H NMR (400 MHz, CDCl_3_), *δ*, ppm: 0.94 (*d*, *J* = 8.0 Hz, 3H), 1.23–1.27 (*m*, 3H), 1.63 (*d*, *J* = 9.2 Hz, 1H), 1.88–1.94 (*m*, 1H), 2.15–2.21 (*m*, 1H), 2.23 (*s*, 3H), 2.68 (*d*, *J* = 9.2 Hz, 1H), 2.98 (*d*, *J* = 9.2 Hz, 1H), 5.08 (*s*, 1H), 6.74–6.78 (*m*, 1H), 6.99 (*d*, *J* = 8.0 Hz, 2H), 7.11–7.14 (*m*, 1H), 7.17 (*d*, *J* = 8.0 Hz, 2H), 7.38–7.42 (*m*, 1H), 7.47–7.50 (*m*, 2H), 7.56 (*d*, *J* = 9.2 Hz, 1H), 7.72 (*d*, *J* = 8.0 Hz, 2H), 9.06 (*d*, *J* = 8.0 Hz, 1H); ^13^C NMR (100 MHz, CDCl_3_), *δ*, ppm: 19.93, 20.84, 33.55, 51.37, 52.04, 65.41, 110.67, 116.20, 119.72, 123.23, 125.66, 126.13, 126.65, 127.34, 128.00, 128.51, 133.91, 135.68, 136.02, 143.44, 143.82; ESI-HRMS, *m*/*z*: Calcd for C_27_H_30_N_3_ [M + H]^+^, 396.2434, found: 396.2445.
*2-Phenyl-3-((4-phenylpiperidin-1-yl)(p-tolyl)methyl)imidazo[1,2-a]pyridine* (**4ab**), light-yellow solid (41 mg, 45%); m.p. 202–204 °C; ^1^H NMR (400 MHz, CDCl_3_), *δ*, ppm: 1.77–1.90 (*m*, 4H), 2.05–2.09 (*m*, 1H), 2.24 (*s*, 3H), 2.31–2.37 (*m*, 1H), 2.56–2.64 (*m*, 1H), 2.86 (*d*, *J* = 9.2 Hz, 1H), 2.16 (*d*, *J* = 9.2 Hz, 1H), 5.16 (*s*, 1H), 6.77–6.81 (*m*, 1H), 7.02 (*d*, *J* = 8.0 Hz, 2H), 7.13–7.26 (*m*, 7H), 7.29–7.33 (*m*, 2H), 7.40–7.44 (*m*, 1H), 7.49–7.53 (*m*, 2H), 7.58 (*d*, *J* = 8.0 Hz, 1H), 7.75 (*d*, *J* = 8.0 Hz, 2H), 9.08 (*d*, *J* = 8.0 Hz, 1H); ^13^C NMR (100 MHz, CDCl_3_), *δ*, ppm: 21.04, 33.66, 34.24, 42.74, 52.79, 53.50, 66.43, 111.88, 117.39, 120.54, 124.39, 126.39, 126.60, 126.84, 127.25, 127.80, 128.46, 128.47, 129.17, 129.61, 134.97, 136.84, 136.91, 144.72, 144.97, 146.21; ESI-HRMS, *m*/*z*: Calcd for C_32_H_32_N_3_ [M + H]^+^, 458.2591, found: 458.2598.

The detailed ^1^H, ^13^C NMR, and ^19^F NMR spectra for all compounds **4a**–**4ab** are provided in the Supplementary Materials.

## 4. Conclusions

In summary, the Y(OTf)_3_-catalyzed three-component aza-Friedel–Crafts reaction was developed for the synthesis of the C_3_-alkylated imidazo[1,2-a]pyridines from imidazo[1,2-a]pyridines, aldehydes, and amines. The developed protocol is operationally simple and provided a wide range of C_3_-alkylated imidazo[1,2-a]pyridines in good to excellent yields. High functional group tolerance and broad substrate scope were the salient features of the method. Additionally, the reaction also achieved gram-scale in excellent yields, showing the possibility of practical application. Moreover, this method may be conveniently applied to the further design and rapid synthesis of potential biologically active imidazo[1,2-a]pyridines with multifunctional groups.

## Figures and Tables

**Table 1:** Optimization of reaction conditions ^[a]^.

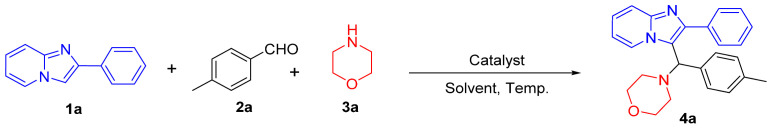				
**Entry**	**Catalyst**	**Solvent**	**Temp. (°C)**	**Yield of 4a (%) ^[b]^**
1	-	Toluene	110	trace
2	TFA (20%)	Toluene	110	trace
3	TsOH (20%)	Toluene	110	trace
4	HFIP (20%)	Toluene	110	trace
5	Sc(OTf)_3_ (20%)	Toluene	110	75
**6**	Y(OTf)_3_ (20%)	Toluene	110	90
7	Y(OTf)_3_ (10%)	Toluene	110	72
8	Y(OTf)_3_ (25%)	Toluene	110	90
9	Y(OTf)_3_ (20%)	DMF	110	42
10	Y(OTf)_3_ (20%)	1,4-Dioxane	110	35
11	Y(OTf)_3_ (20%)	Acetonitrile	110	18
12	Y(OTf)_3_ (20%)	Toluene	100	62
13	Y(OTf)_3_ (20%)	Toluene	120	90
14 ^[c]^	Y(OTf)_3_ (20%)	Toluene	110	66
15 ^[d]^	Y(OTf)_3_ (20%)	Toluene	110	82
16 ^[e]^	Y(OTf)_3_ (20%)	Toluene	110	88

^[a]^ Reaction conditions: **1a** (0.2 mmol), **2a** (0.3 mmol, 1.5 equiv.), **3a** (0.4 mmol, 2 equiv.), and solvent (1.0 mL), 12 h. ^[b]^ Isolated yield. ^[c]^ **1a** (0.2 mmol), **2a** (0.2 mmol, 1.0 equiv.), and **3a** (0.2 mmol, 1.0 equiv.). ^[d]^ **1a** (0.2 mmol), **2a** (0.3 mmol, 1.5 equiv.), and **3a** (0.3 mmol, 1.5 equiv.). ^[e]^ **1a** (0.2 mmol), **2a** (0.4 mmol, 2.0 equiv.), and **3a** (0.4 mmol, 2.0 equiv.).

## Data Availability

All data supporting the findings of this study are available within the paper and within its Supplementary Materials published online.

## Supplementary Materials

The following supporting information can be downloaded at: https://www.mdpi.com/article/10.3390/molecules29153463/s1, which contains details on the crystallographic information parameters (Table S1), ^1^H, ^13^C, and ^19^F NMR spectra for all compounds **4a**–**4ab**.
