# Supplementary material for: C3-Alkylation of Imidazo[1,2-a]pyridines via Three-Component Aza-Friedel–Crafts Reaction Catalyzed by Y(OTf)3
Source: Molecules. 2024 Jul 24;29(15):3463. doi: 10.3390/molecules29153463 (PMC11313794; doi:10.3390/molecules29153463)
Supplement: Supplementary file 1 [file molecules-29-03463-s001.zip › molecules-3102843-supplementary.pdf]

Supplementary Materials

C_3-Alkylation of Imidazo[1,2-a]pyridines via Three-Component Aza-Friedel–Crafts Reaction Catalyzed by $Y(OTf)_3$

Kai Yang [1,*] , Cai-Bo Chen [1], Zhao-Wen Liu [1] , Zhen-Lin Li [1], Yu Zeng [2] and Zhao-Yang Wang [2,*]

1 College of pharmacy, Gannan Medical University, Ganzhou 341000, China; ccb18046698424@outlook.com (C.-B.C.); liuzhaowenyifan@126.com (Z.-W.L.); lizhenlin1@gmu.cn (Z.-L.L.)

2 School of Chemistry, South China Normal University, Guangzhou Key Laboratory of Analytical Chemistry for Biomedicine, GDMPA Key Laboratory for Process Control and Quality Evaluation of Chiral Pharmaceuticals, Key Laboratory of Theoretical Chemistry of Environment, Ministry of Education, Guangzhou 510006, China; 2023022534@m.scnu.edu.cn

* Correspondence: kai_yang@gmu.edu.cn (K.Y.); wangzy@scnu.edu.cn (Z.-Y.W.);
Tel.: +86-0797-8169782 (K.Y.); +86-020-3931-0258 (Z.-Y.W.); Fax: +86-020-3931-0187 (Z.-Y.W.)

Table of Contents

General Information..[2]

Experimental Procedure for Compounds **1a-1n**..[3]

Experimental Procedure for Compounds **4a-4ab**..[4]

Data of Single-crystal X-ray Analysis for **4a**..[5]

Mechanism investigation..[6-7]

^{1}H, ^{13}C and ^{19}F NMR Spectra for All Products **4a-4ab**..[8-37]

References..[38]

General Information

Melting point (m.p.) was performed on a Büchi Melting Point B-545 instrument without correcting. The data of ^{1}H, ^{13}C and ^{19}F NMR spectra were collected on a BRUKER DRX-400 spectrometer using tetramethylsilane (TMS) as an internal standard.

High-resolution mass spectra (HRMS) were obtained with a LCMS-IT-TOF mass spectrometer. Single-crystal X-ray analysis was obtained using Bruker APEX2 Smart CCD. TLC was performed on commercially prepared 100-400 mesh silica gel plates (GF254) and visualization was detected at 254 or 365 nm.

All reagents and solvents were purchased from commercial sources and used without further purification. 2-Substituted imidazo[1,2-a]pyridines **1** were synthesized from 2-bromoacetophenones and various 2-aminopyridines (see the following for details) [1,2].

Experimental Procedure for Compounds 1a-1n

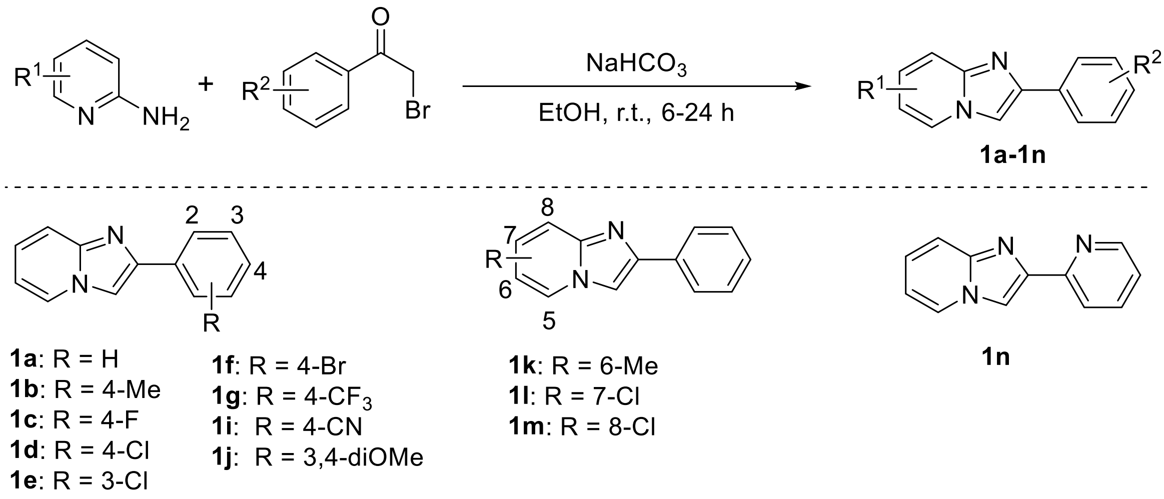

Compounds **1a**-**1n** were synthesized according to the reported procedure [1,2]. $NaHCO_3$ (24 mmol, 1.2 equiv.) was added to the ethanol solution containing 2-bromoacetophenones (20 mmol, 1.0 equiv) and 2-aminopyridines (22 mmol, 1.1 equiv.). Then, the reaction mixture was stirred at room temperature for 6-24 hours. After completion of the reaction, the resulting mixture was diluted with water (15 mL) and extracted with ether (3 × 20 mL). The combined organic layer was washed with brine (25 mL) and dried with anhydrous $MgSO_4$, then concentrated under vacuum. The analytically pure 2-arylimidazo[1,2-a]pyridines **1a**-**1n** were obtained by silica gel column with petroleum ether/EtOAc as the eluent with 50-90% yields.

Experimental Procedure for Compounds 4a-4ab

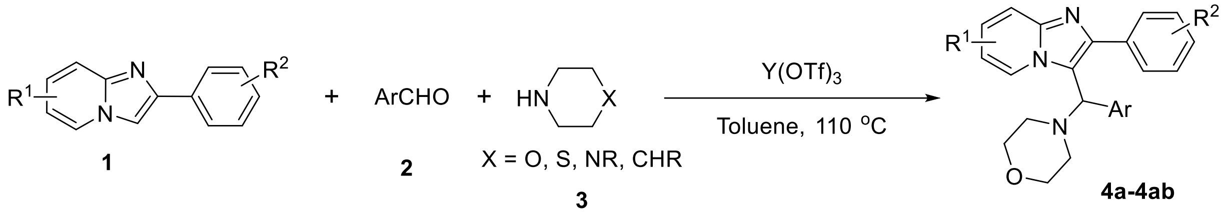

A mixture of imidazo[1,2-a]pyridine **1** (0.2 mmol, 1.0 equiv.), aromatic aldehyde **2** (0.3 mmol, 1.5 equiv.), cycloamine **3** (0.4 mmol, 2 equiv.) and $Y(OTf)_3$ (0.04 mmol, 0.2 equiv.) in toluene (1.0 mL) was stirred at 110 °C for 12 h. After the completion of the reaction, the reaction mixture was quenched with H_2O (15 mL) and extracted with ethyl acetate (3 × 15 mL). Then, the organic layer was dried over anhydrous Na_2SO_4. After filtration and evaporation of the solvents under reduced pressure, the crude product was purified by column chromatography on silica gel to afford the desired product **4**.

Data of Single-crystal X-ray Analysis

Table S1. Crystal data and structure refinement for **4a**

Compound	**4a**
Empirical formula	$C_{25}H_{26}N_3O$
Formula weight	382.49
Temperature/K	293(2)
Crystal system	Monoclinic
Space group	*C2/c*
a (Å)	28.454(9)
b (Å)	16.626(4)
c (Å)	11.930(3)
α (°)	90
β (°)	101.57(3)
γ (°)	90
Volume(Å^3)	5529(2)
Z	8
ρ_{calc}g/cm^3	0.924
μ /mm^{-1}	0.057
F (000)	1640.0
Crystal size/mm^3	0.08 * 0/06 * 0.04
Radiation	MoKα (λ = 0.71073)
2Θ range for data collection/°	4.726 to 58.182
Index ranges	-22≤h≤37, -22≤k≤19, -16≤l≤ 15
Reflections collected	12814
Independent reflections	6361 [Rint = 0.0714, Rsigma = 0.1707]
Data/restraints/parameters	6361/0/263
Goodness-of-fit on F2	0.975
Final R indexes [I>=2σ (I)]	R1 = 0.1019, wR2 = 0.2316
Final R indexes [all data]	R1 = 0.2011, wR2 = 0.2862
Largest diff. peak/hole / e Å^{-3}	0.24/-0.26

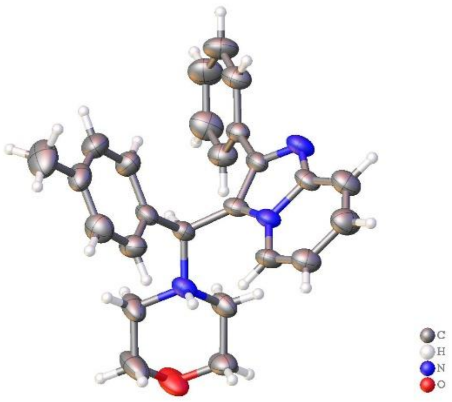

Figure S1. The molecular structure of compound **4a**.

Mechanism investigation

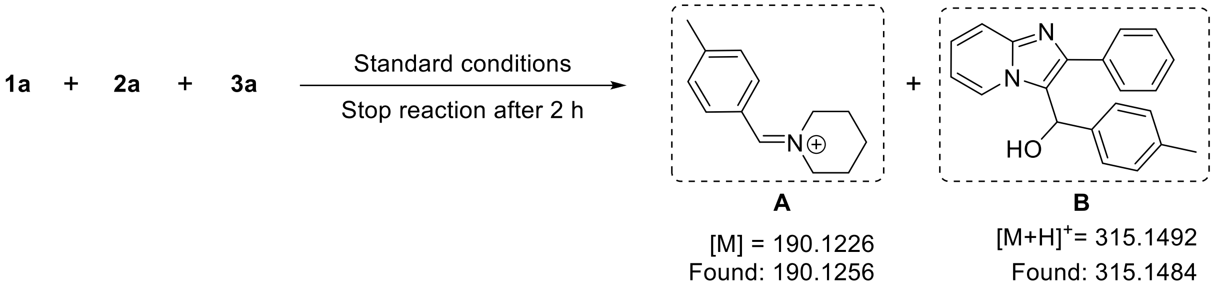

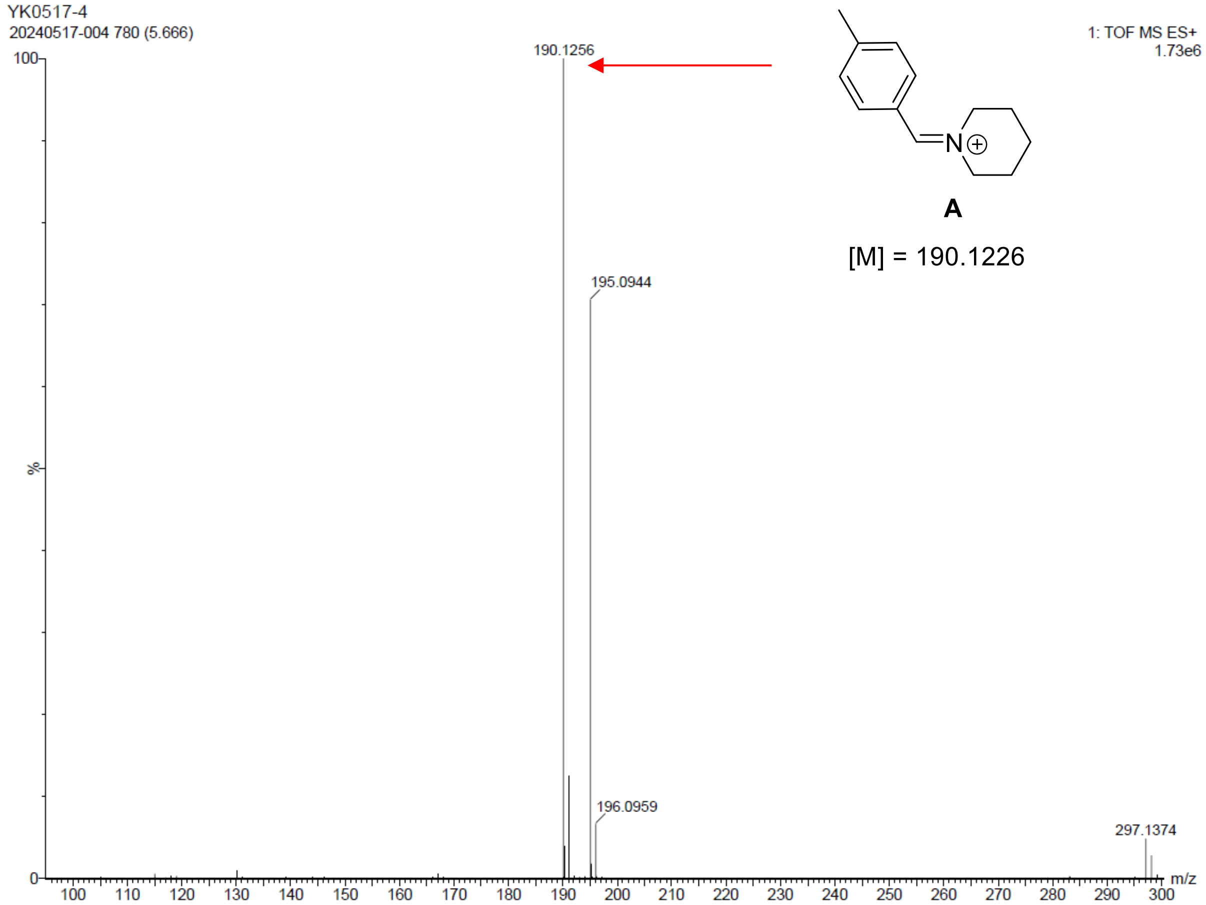

Figure S2. ESI-HRMS spectrum of imine **A**.

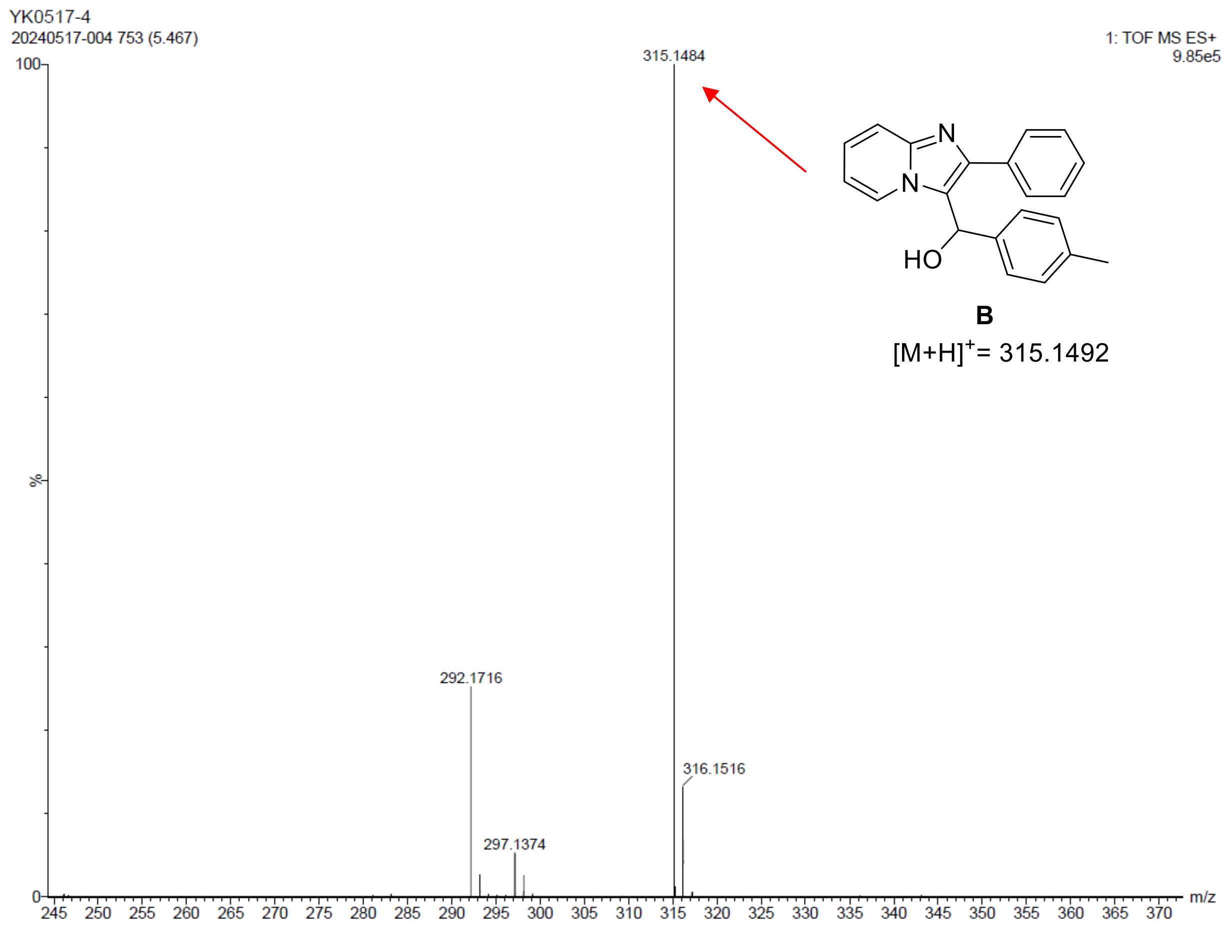

Figure S3. ESI-HRMS spectrum of benzyl alcohol **B**.

NMR Spectra for All Compounds 4a-4ab

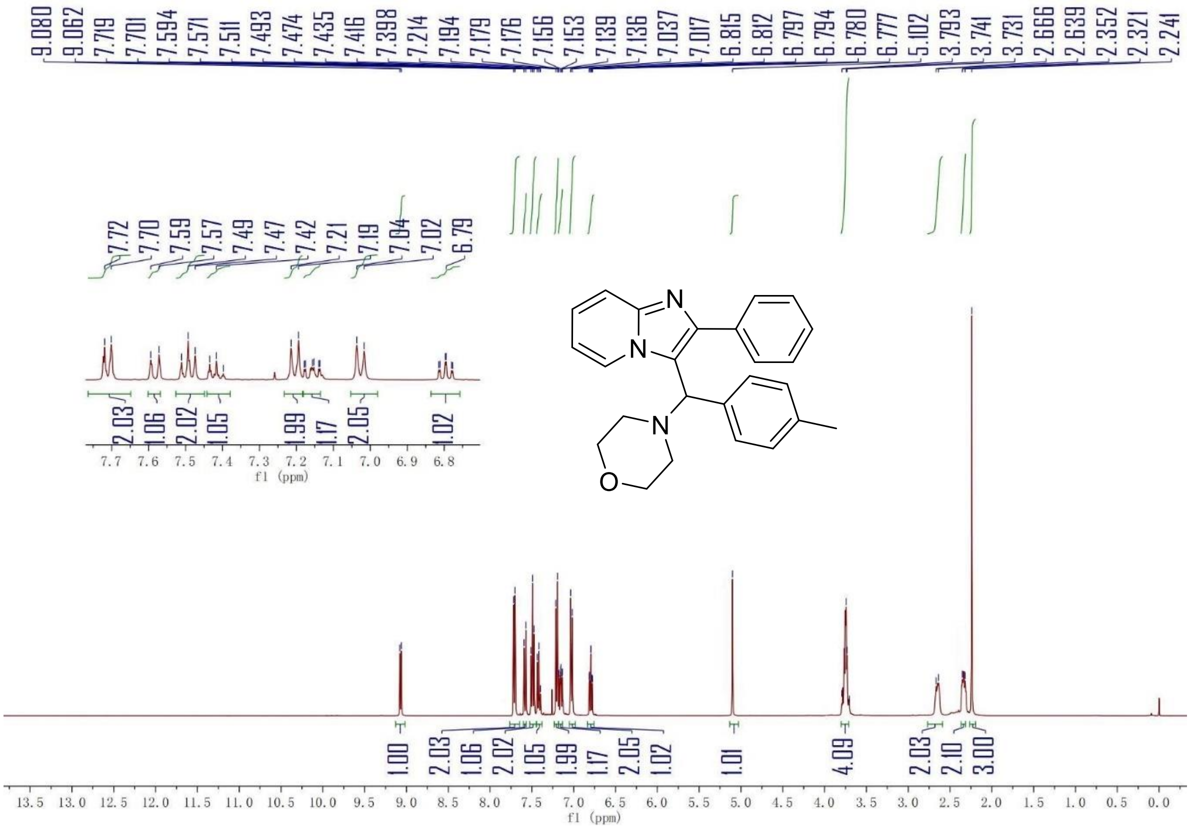

^{1}H NMR spectrum of compound **4a**

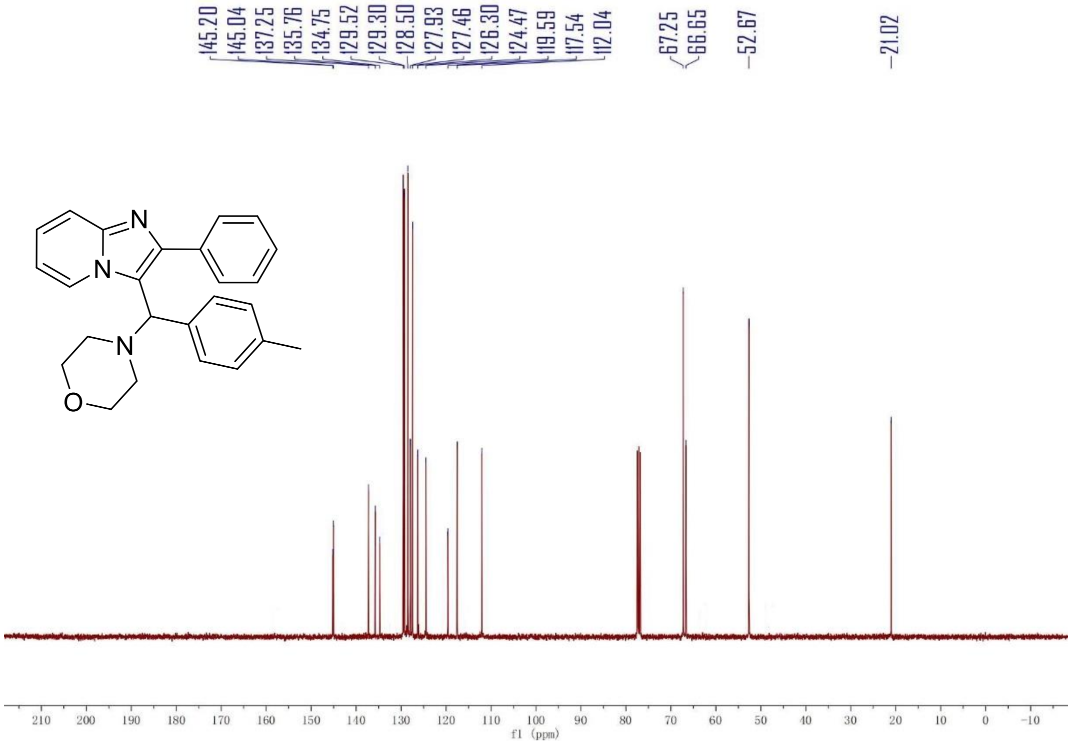

^{13}C NMR spectrum of compound **4a**

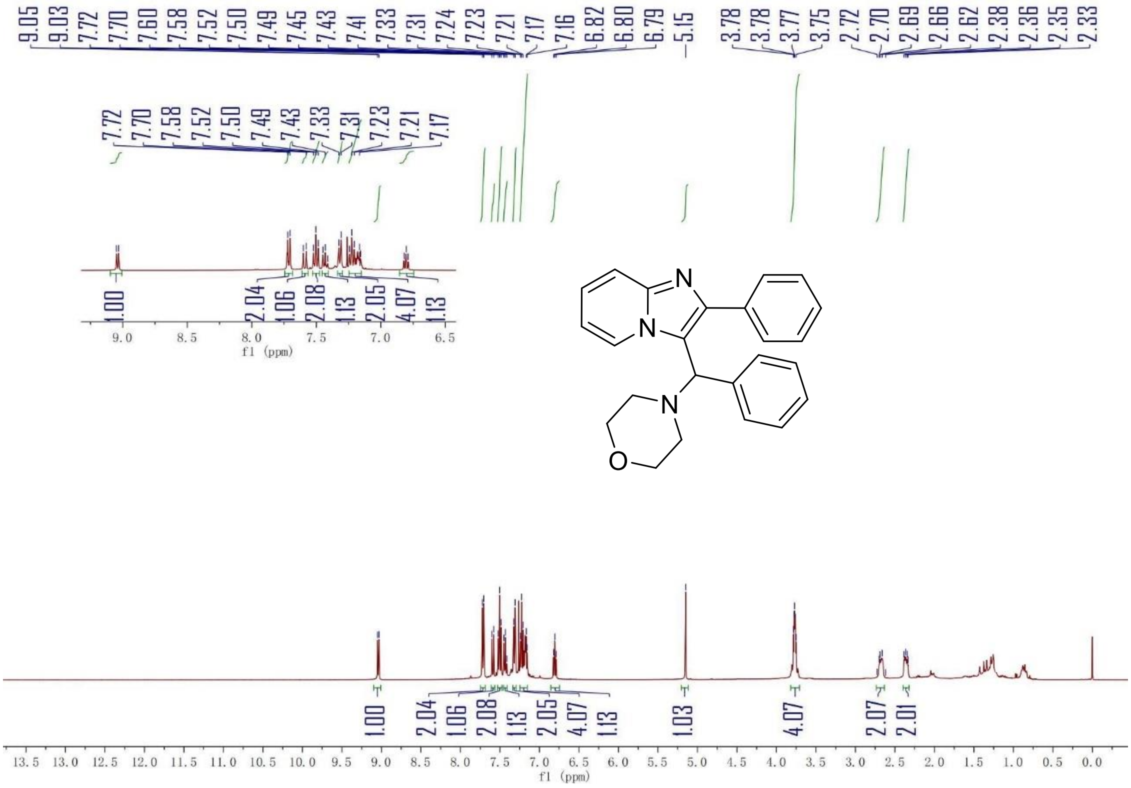

^{1}H NMR spectrum of compound **4b**

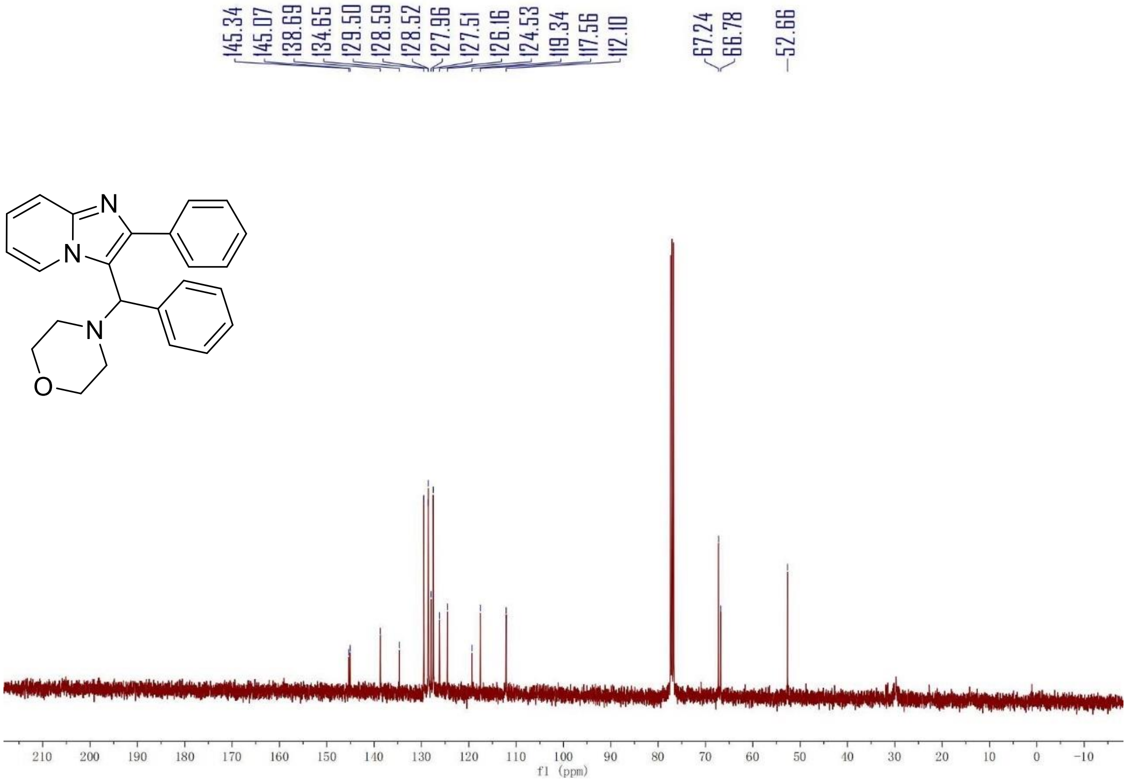

^{13}C NMR spectrum of compound **4b**

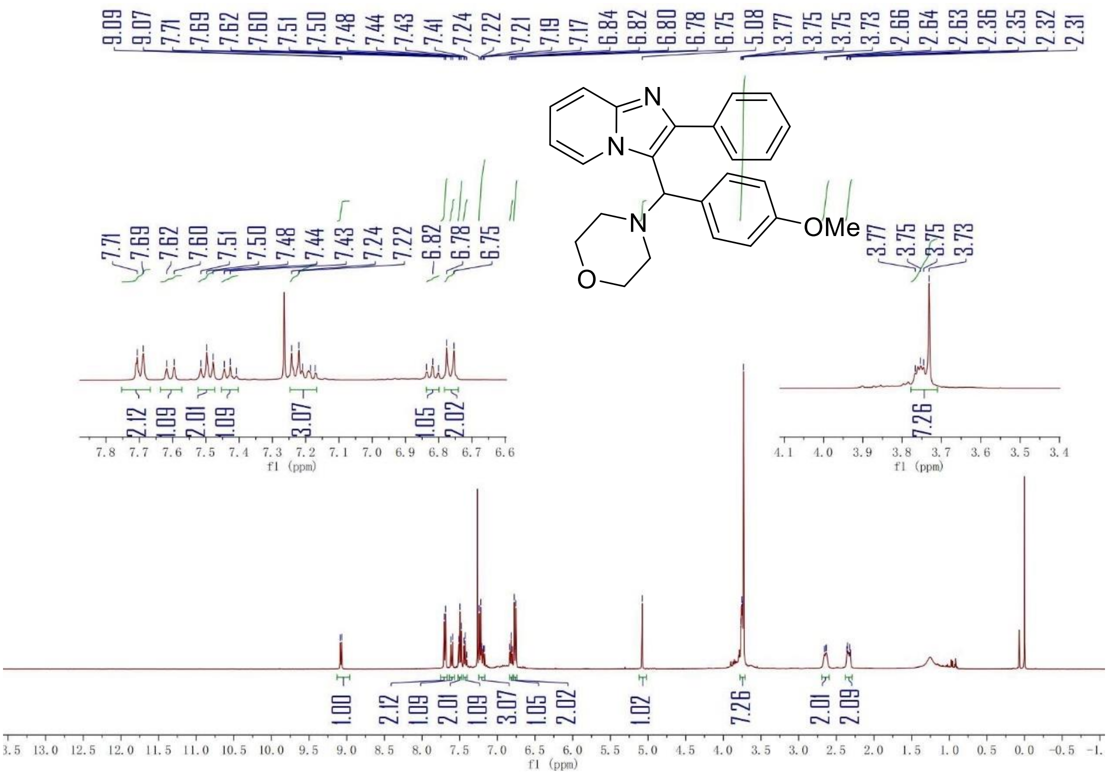

^{1}H NMR spectrum of compound **4c**

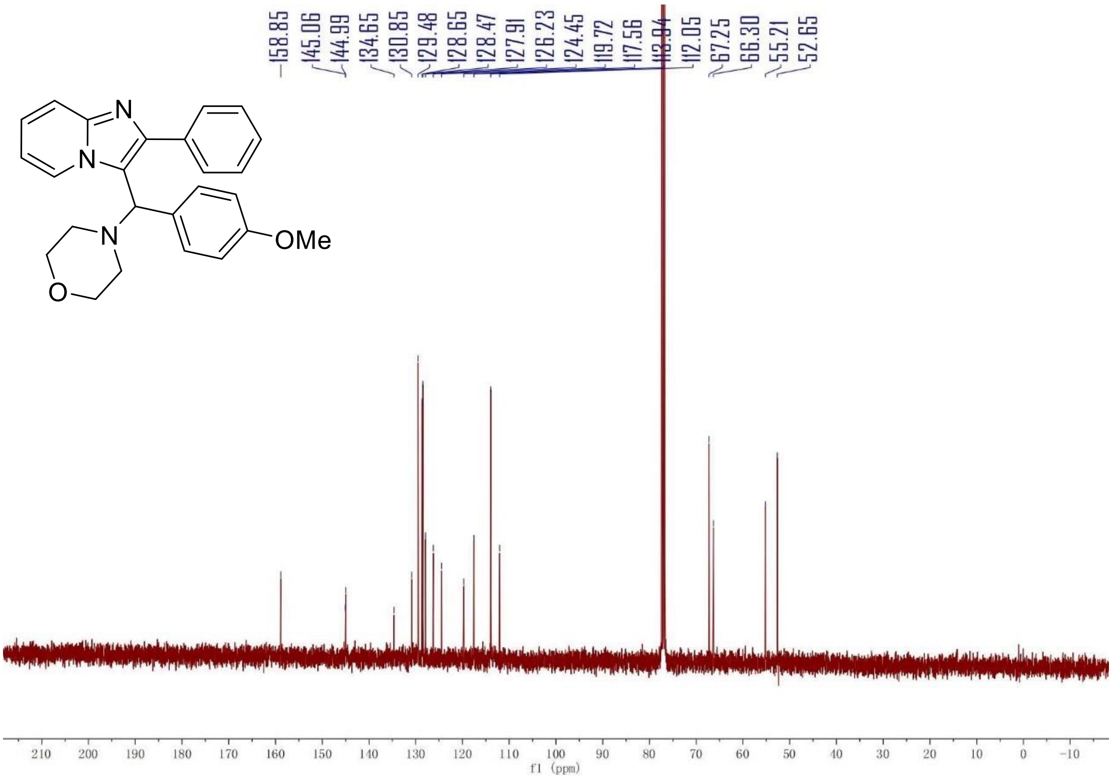

^{13}C NMR spectrum of compound **4c**

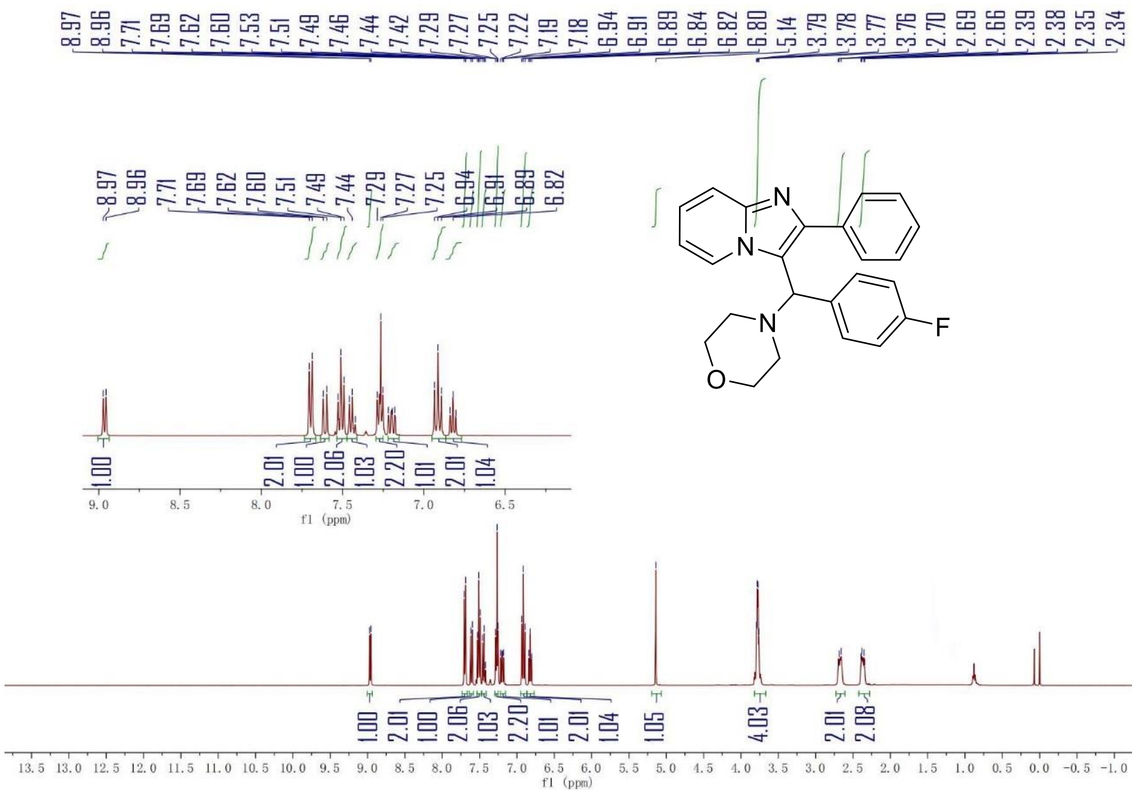

^{1}H NMR spectrum of compound **4d**

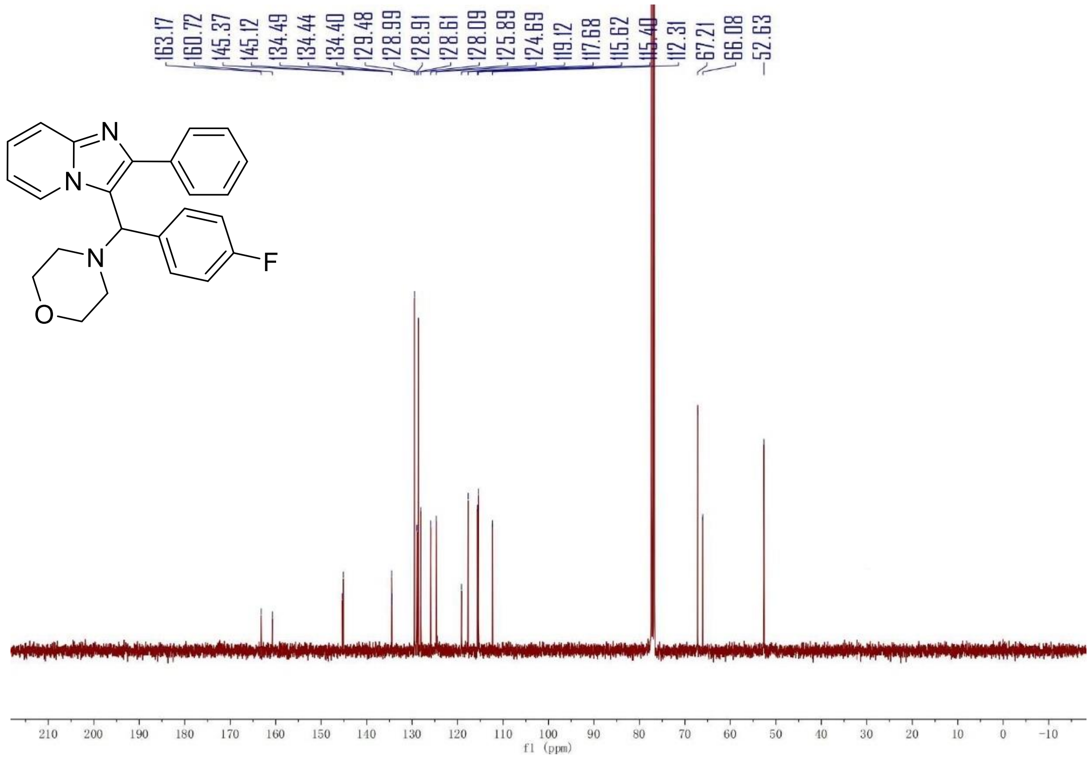

^{13}C NMR spectrum of compound **4d**

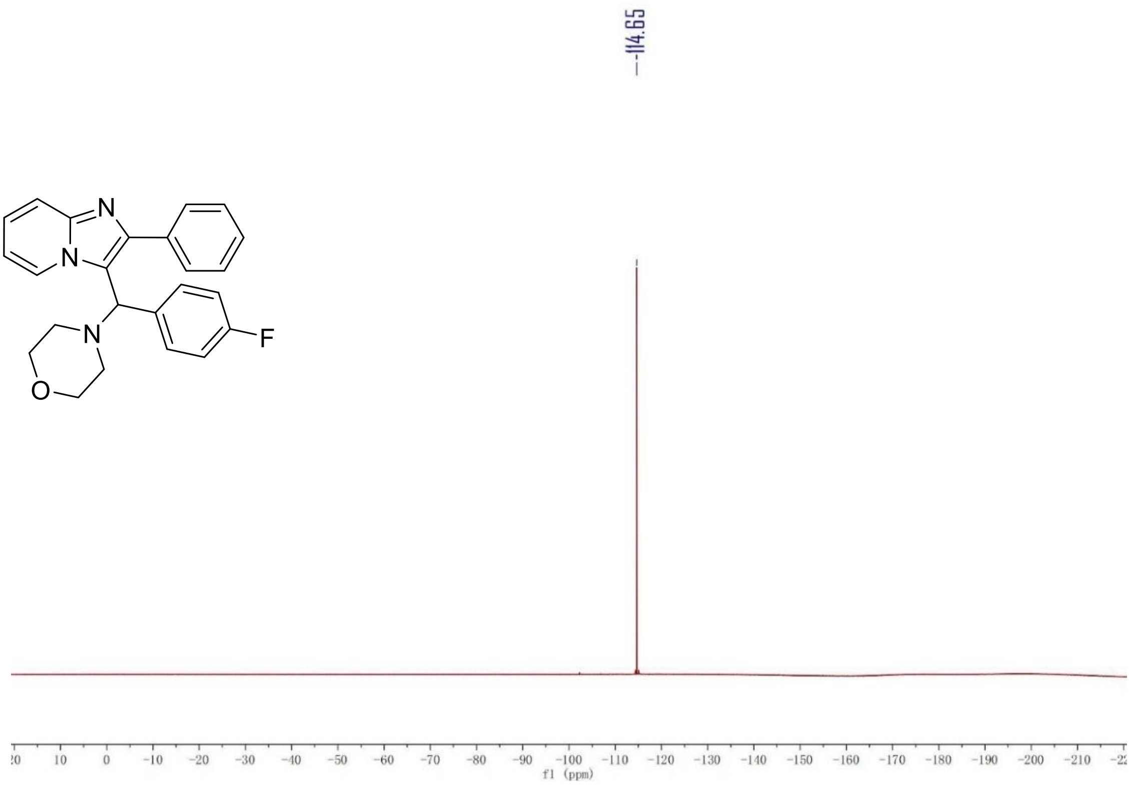

^{19}F NMR spectrum of compound **4d**

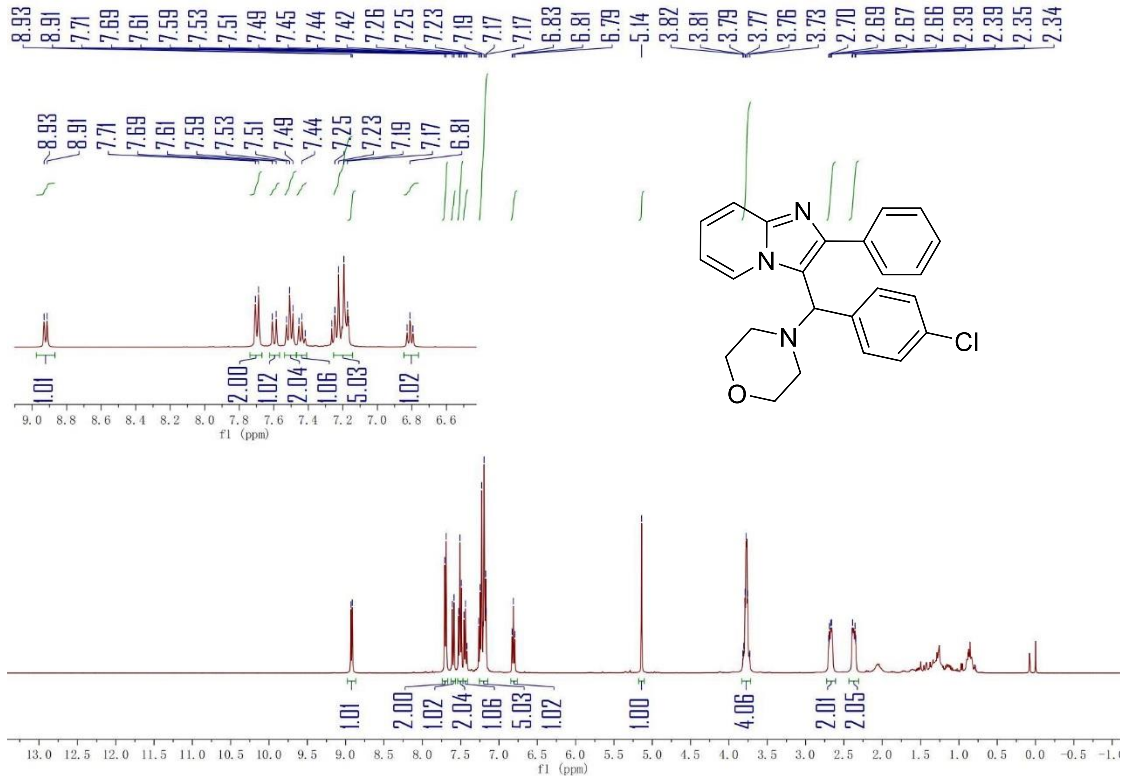

^{1}H NMR spectrum of compound **4e**

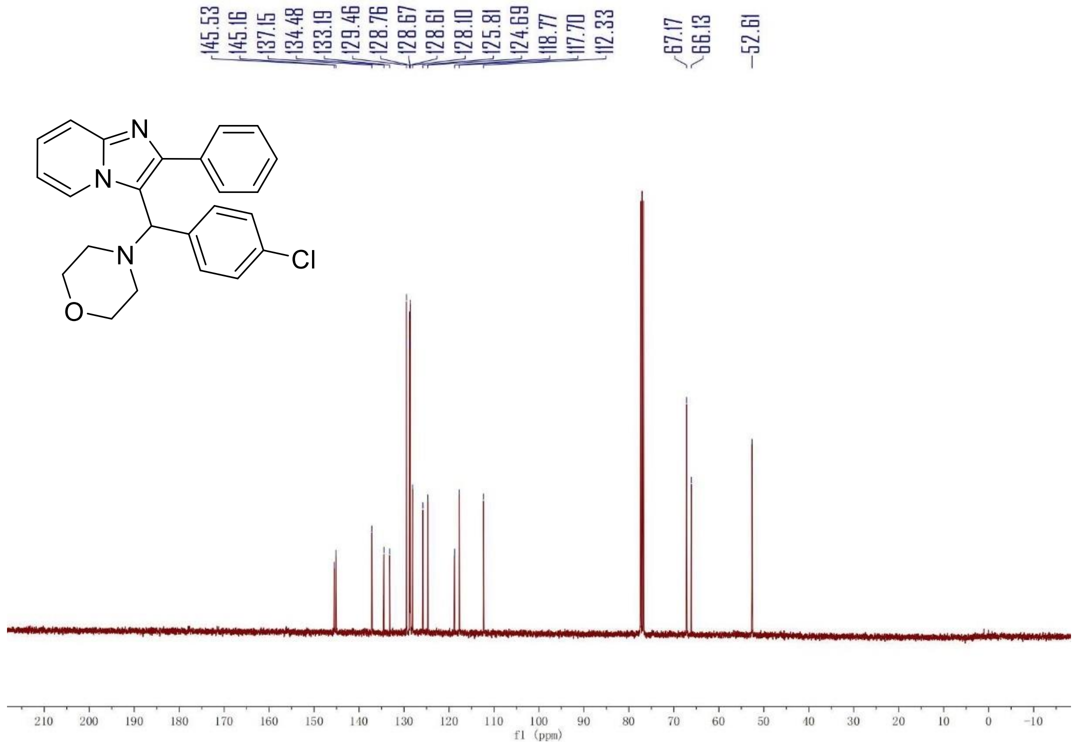

^{13}C NMR spectrum of compound **4e**

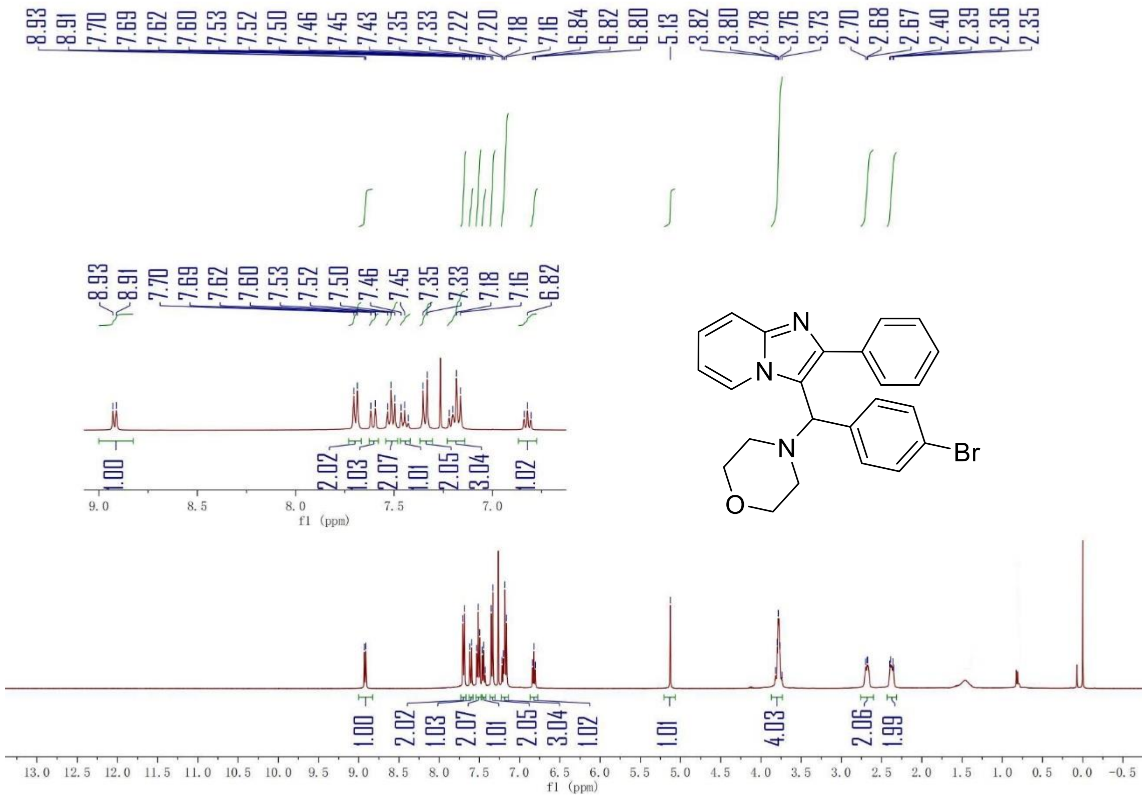

^{1}H NMR spectrum of compound **4f**

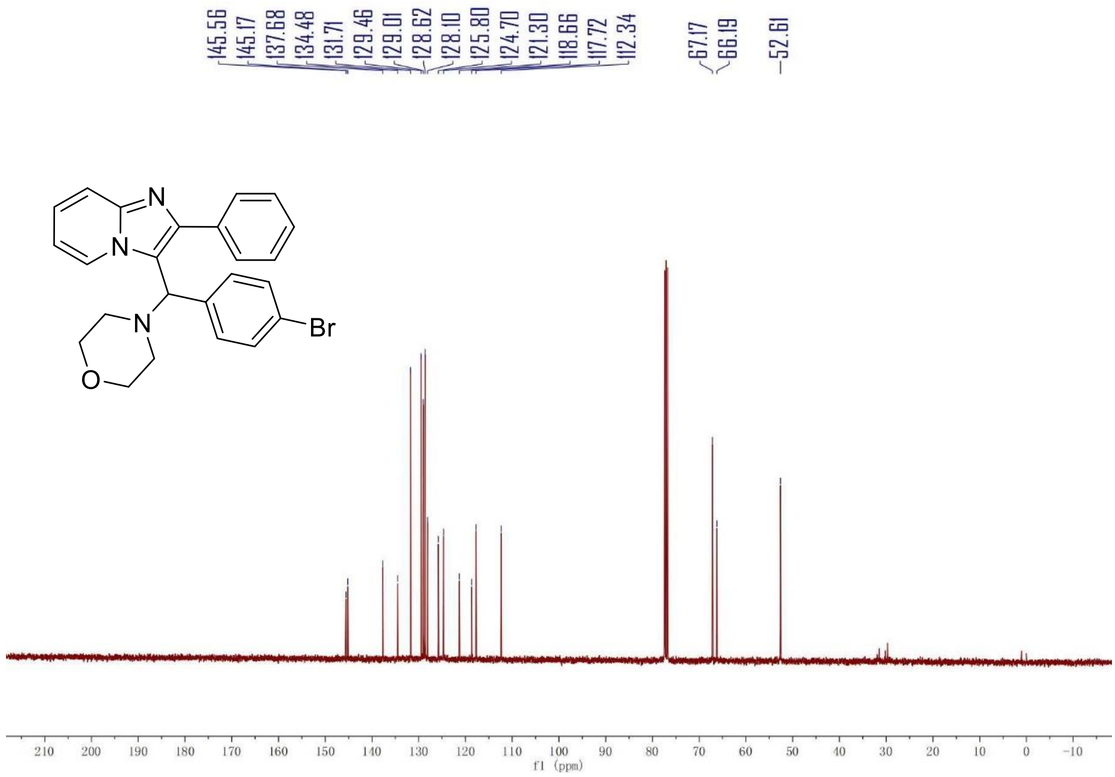

^{13}C NMR spectrum of compound **4f**

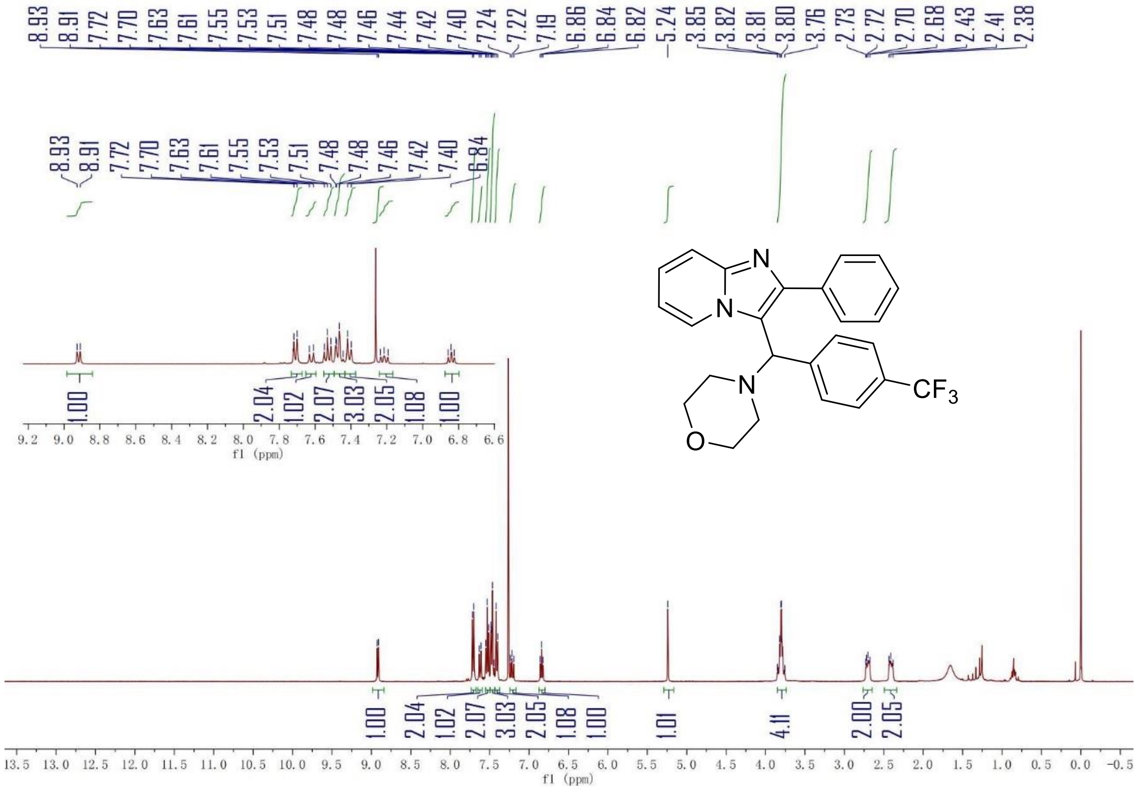

^{1}H NMR spectrum of compound **4g**

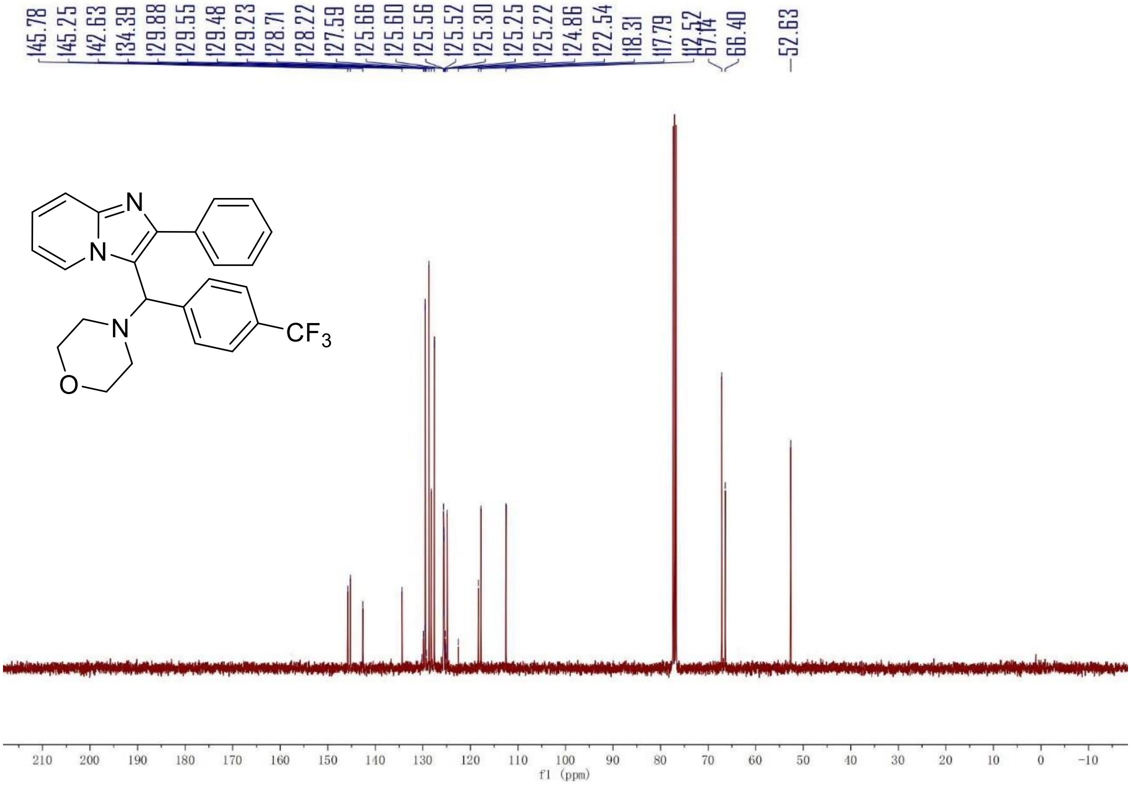

^{13}C NMR spectrum of compound **4g**

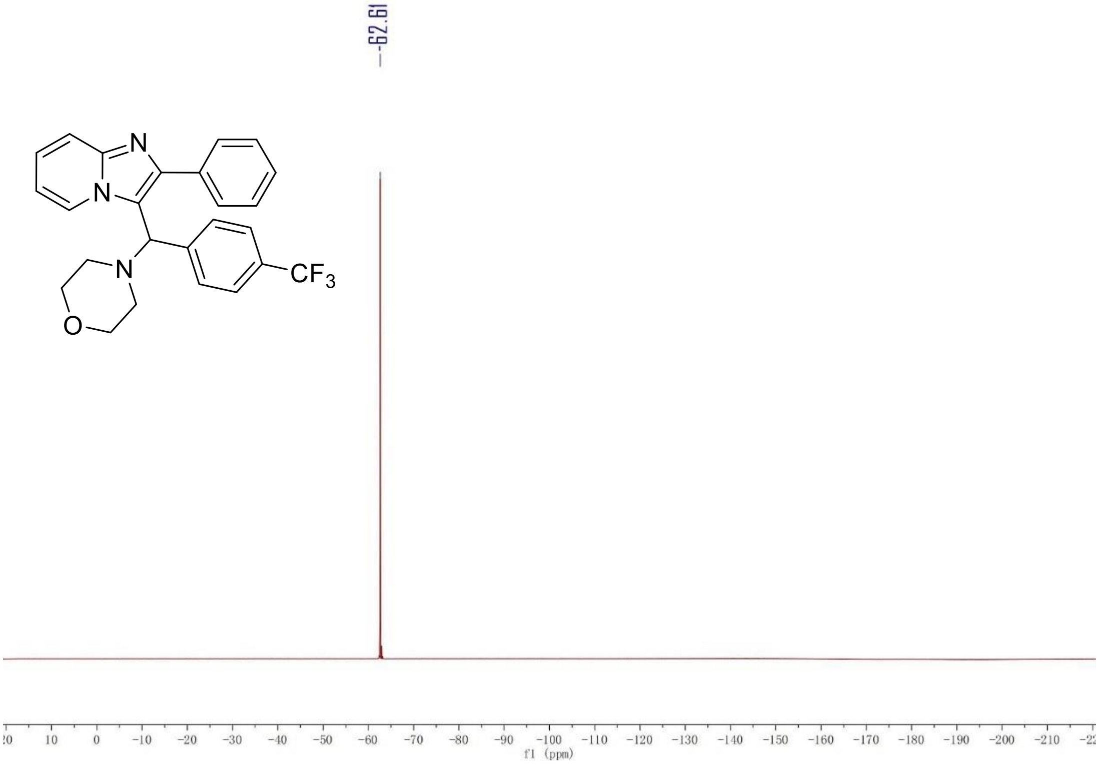

^{19}F NMR spectrum of compound **4g**

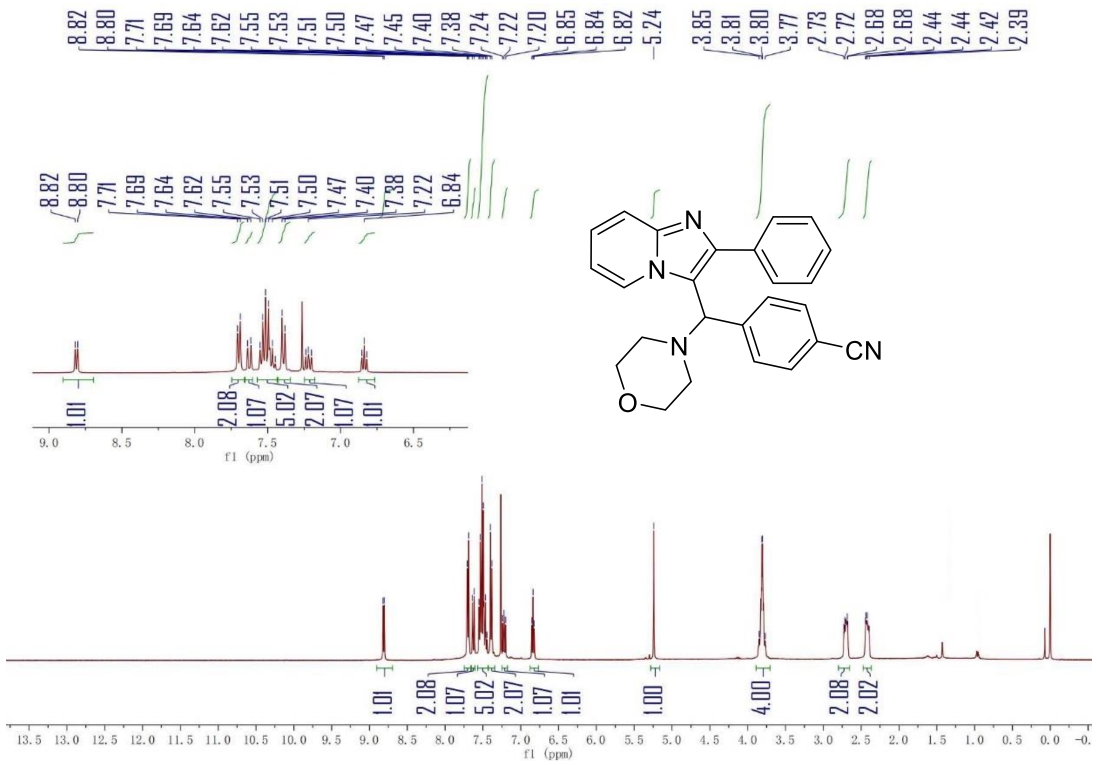

^{1}H NMR spectrum of compound **4h**

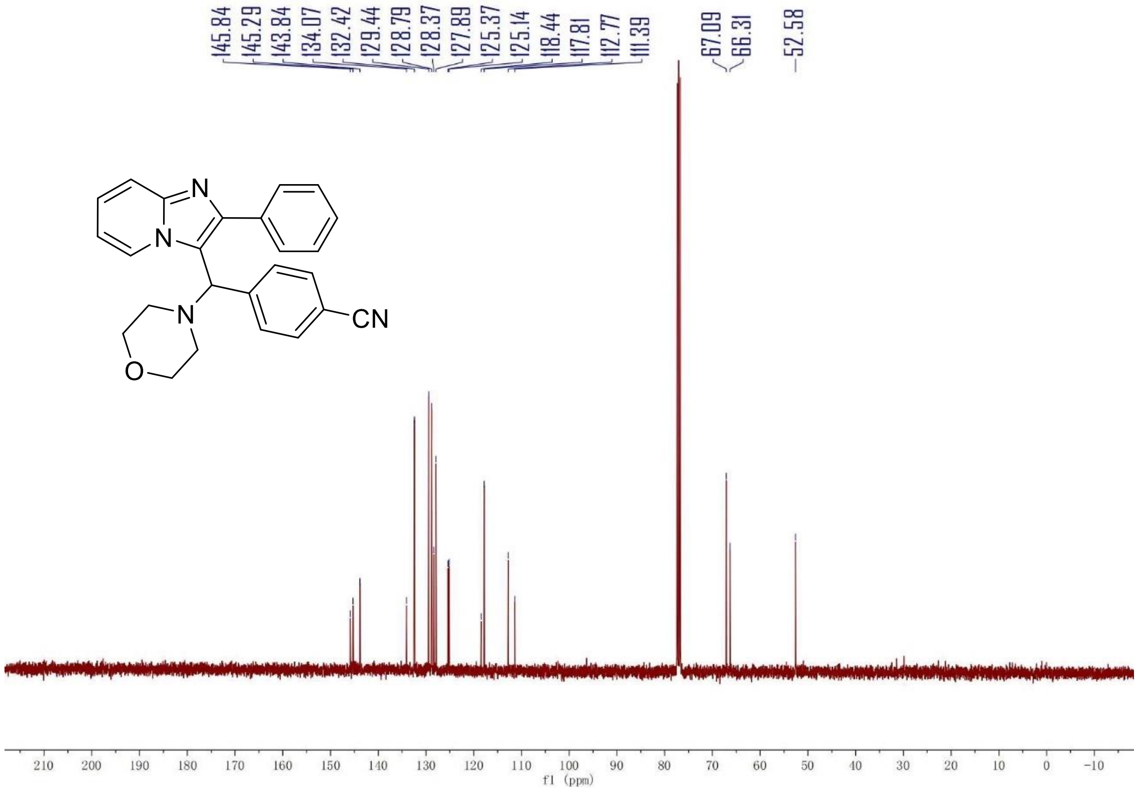

^{13}C NMR spectrum of compound **4h**

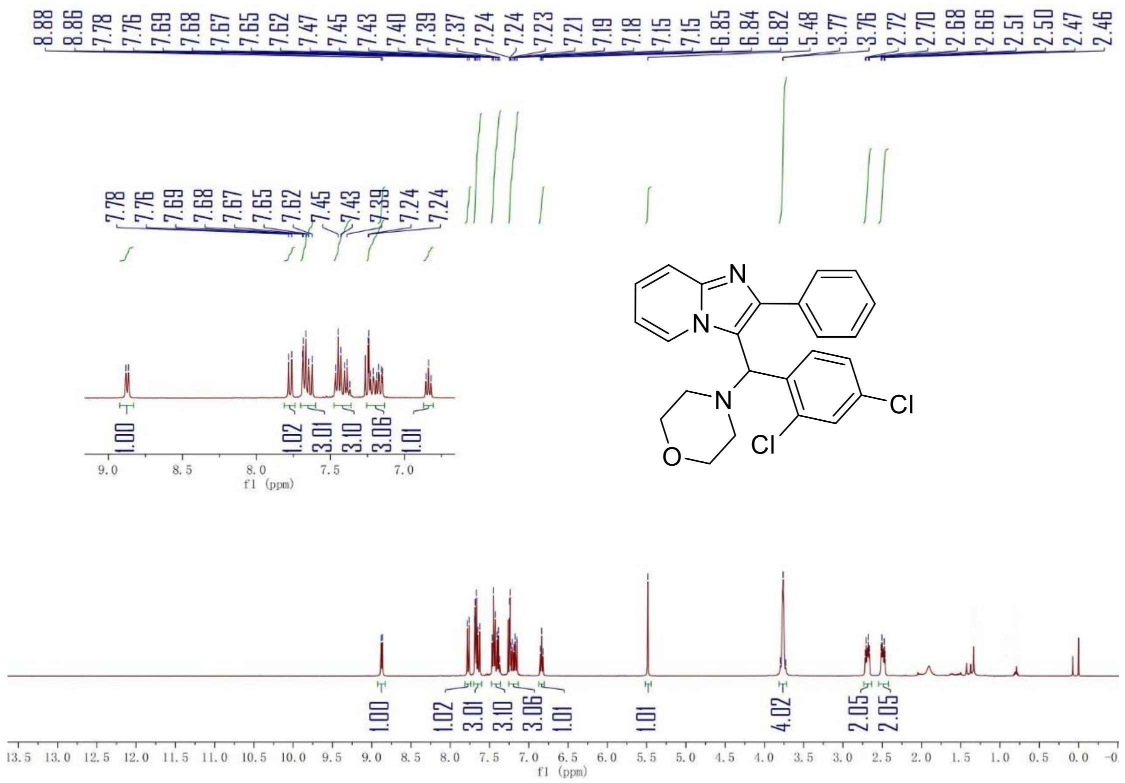

^{1}H NMR spectrum of compound **4i**

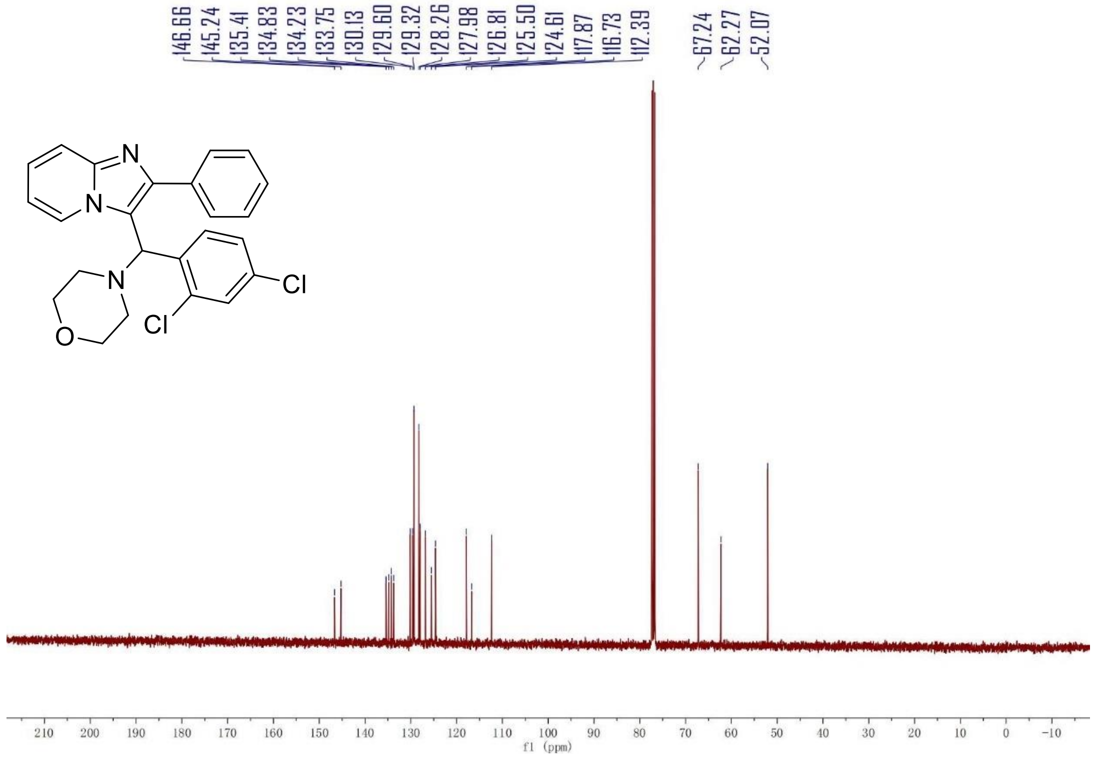

^{13}C NMR spectrum of compound **4i**

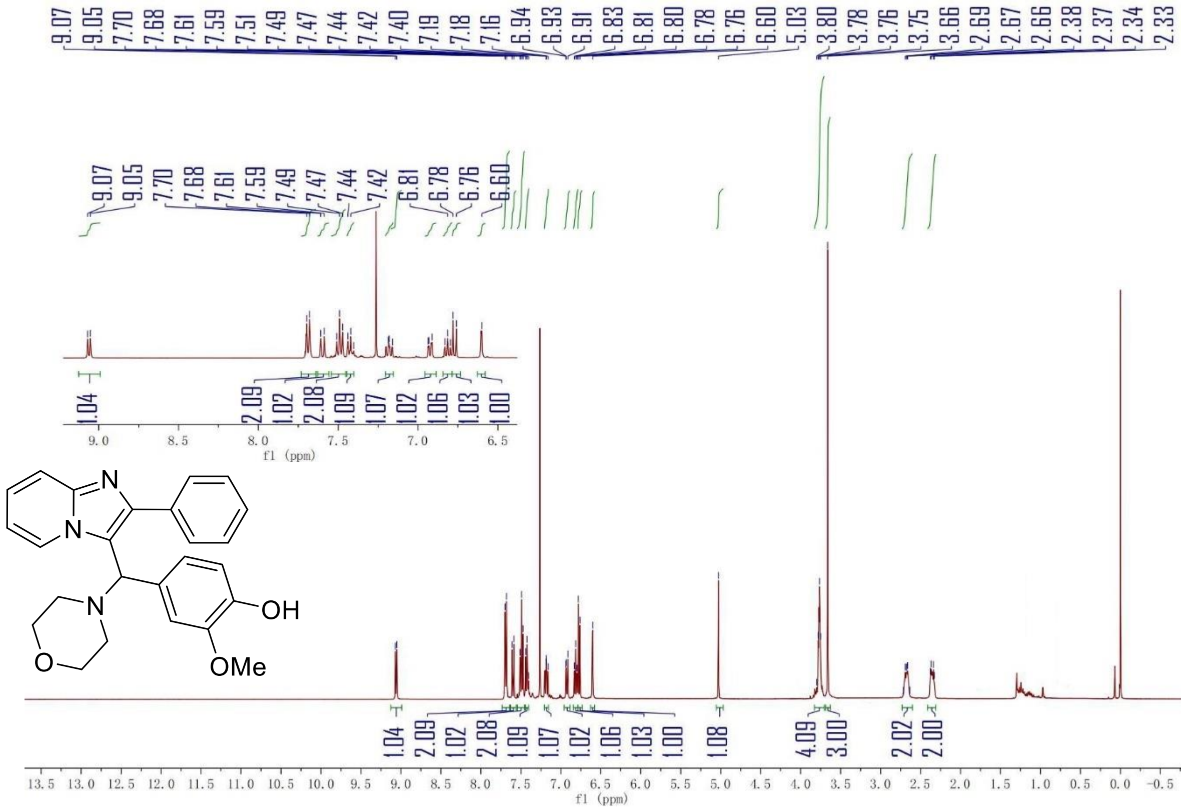

^{1}H NMR spectrum of compound **4j**

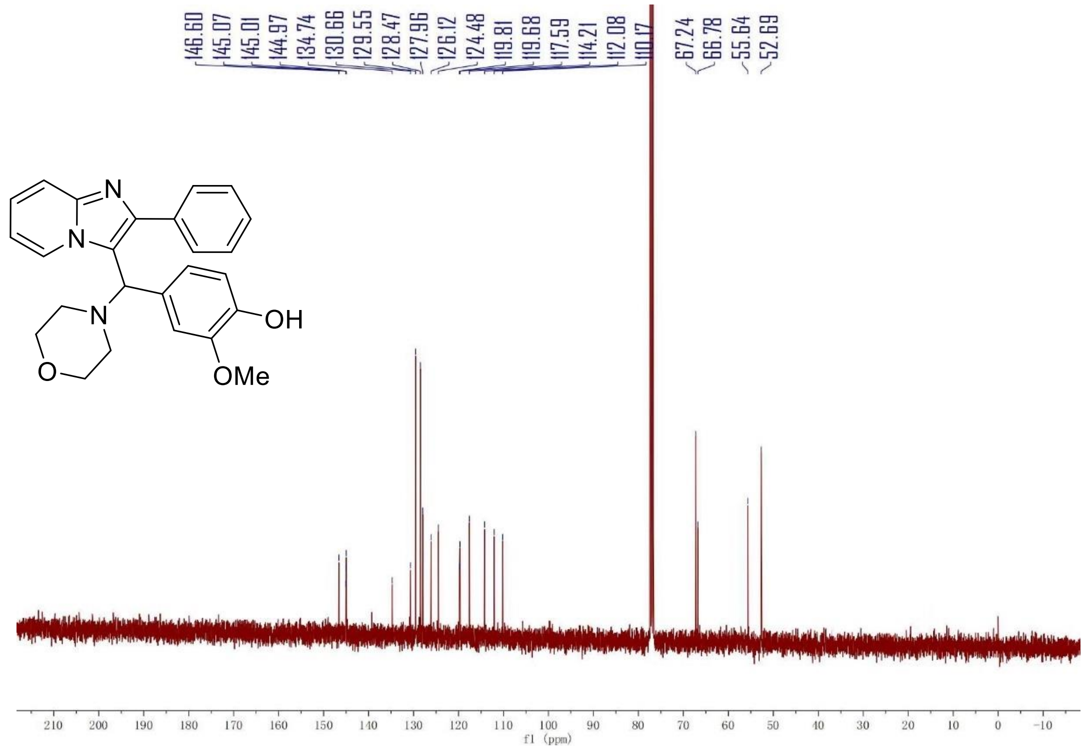

^{13}C NMR spectrum of compound **4j**

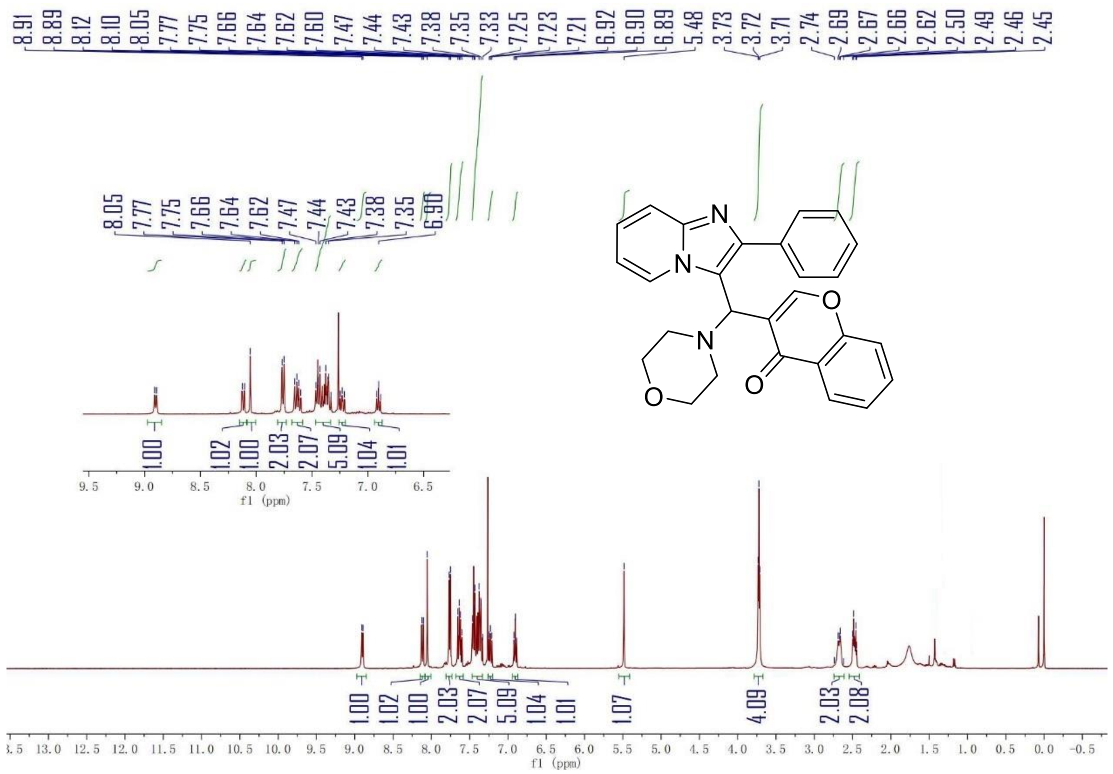

^{1}H NMR spectrum of compound **4k**

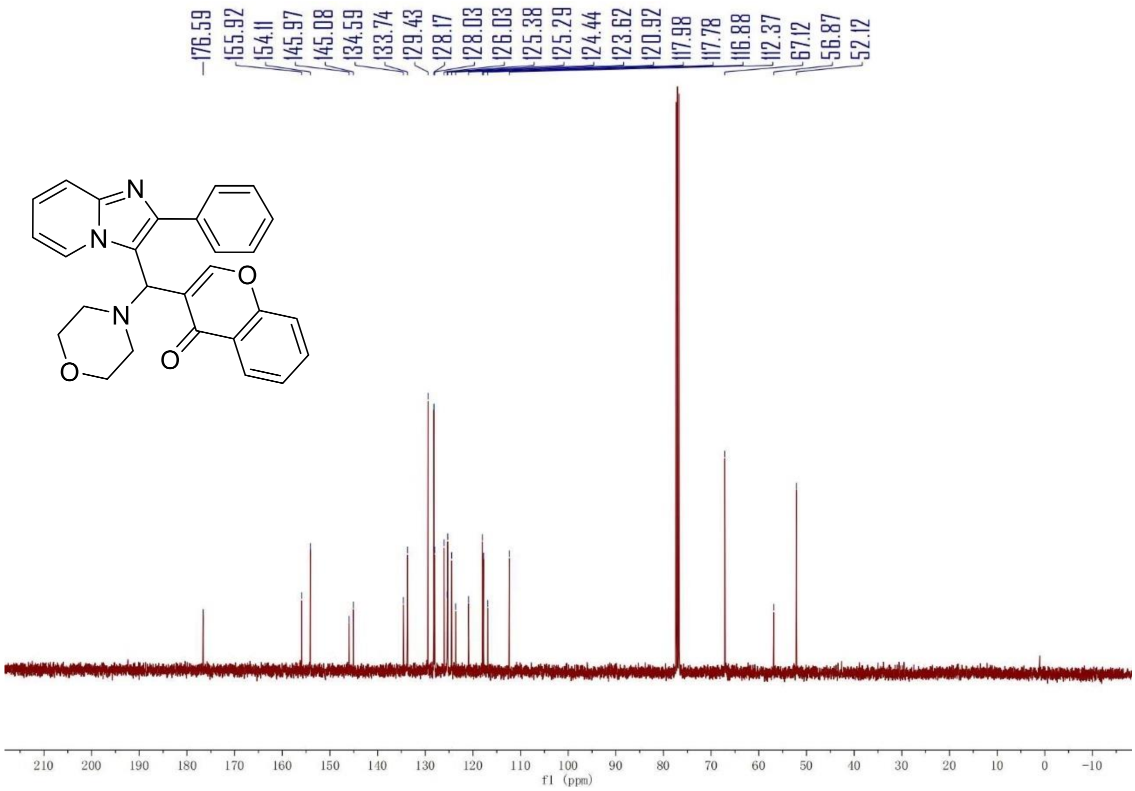

^{13}C NMR spectrum of compound **4k**

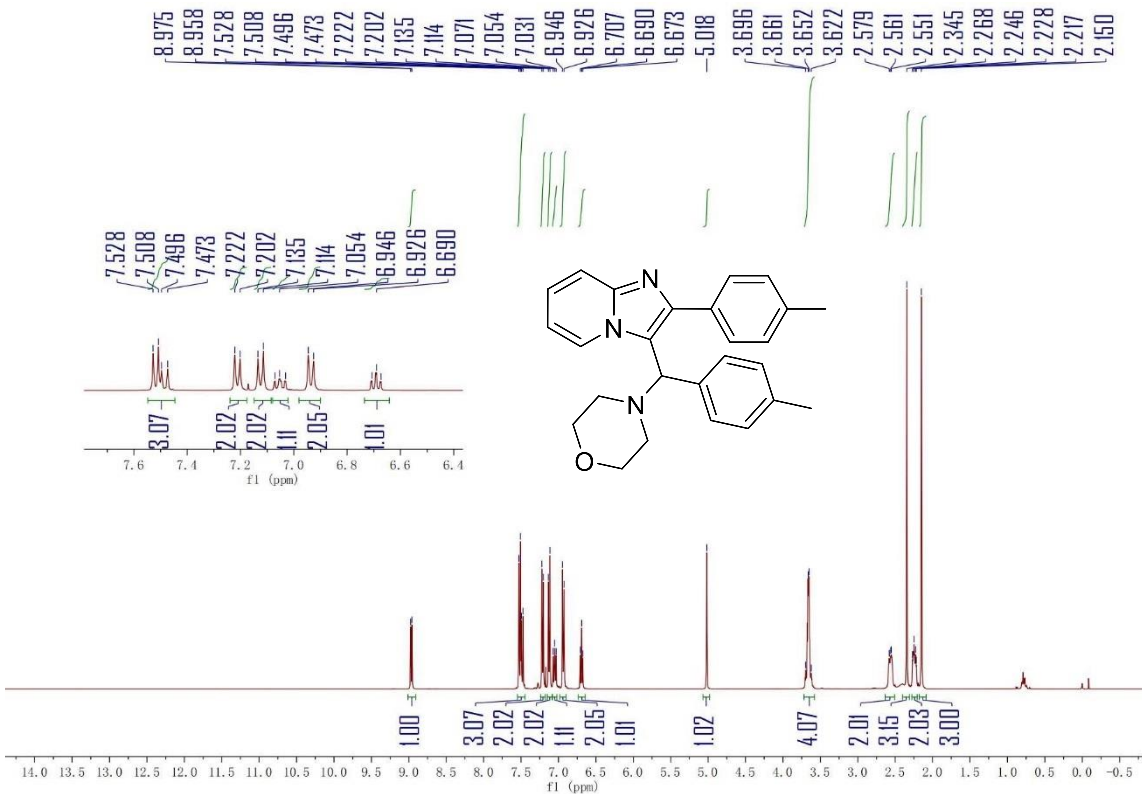

^{1}H NMR spectrum of compound **4l**

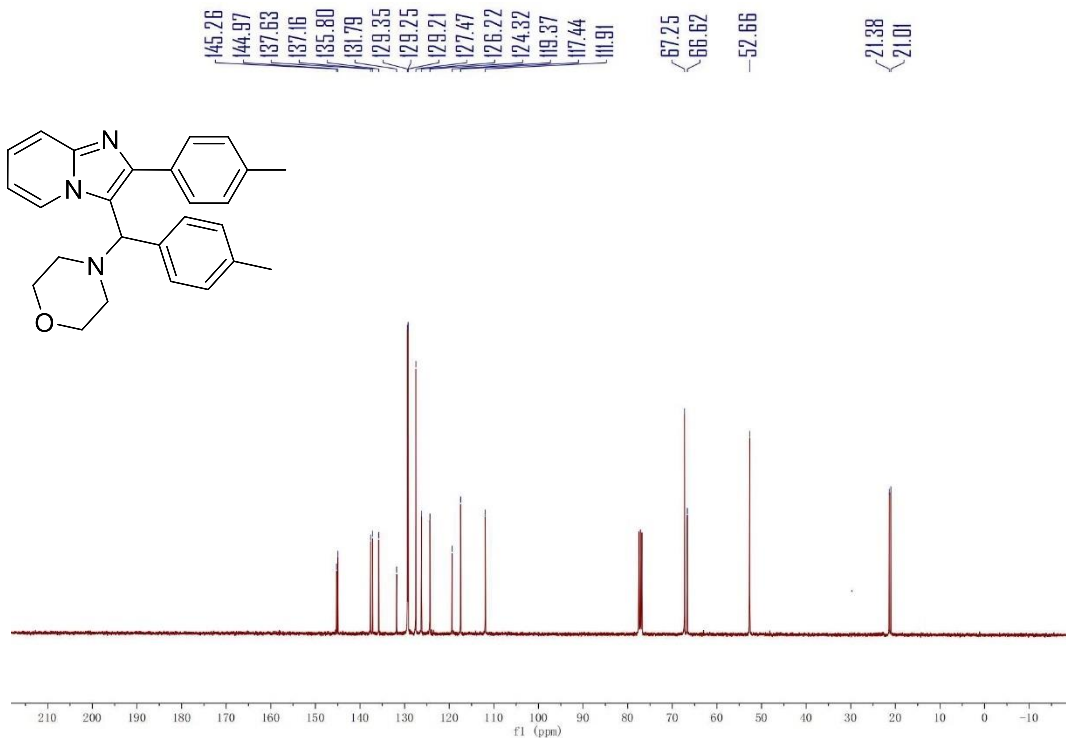

^{13}C NMR spectrum of compound **4l**

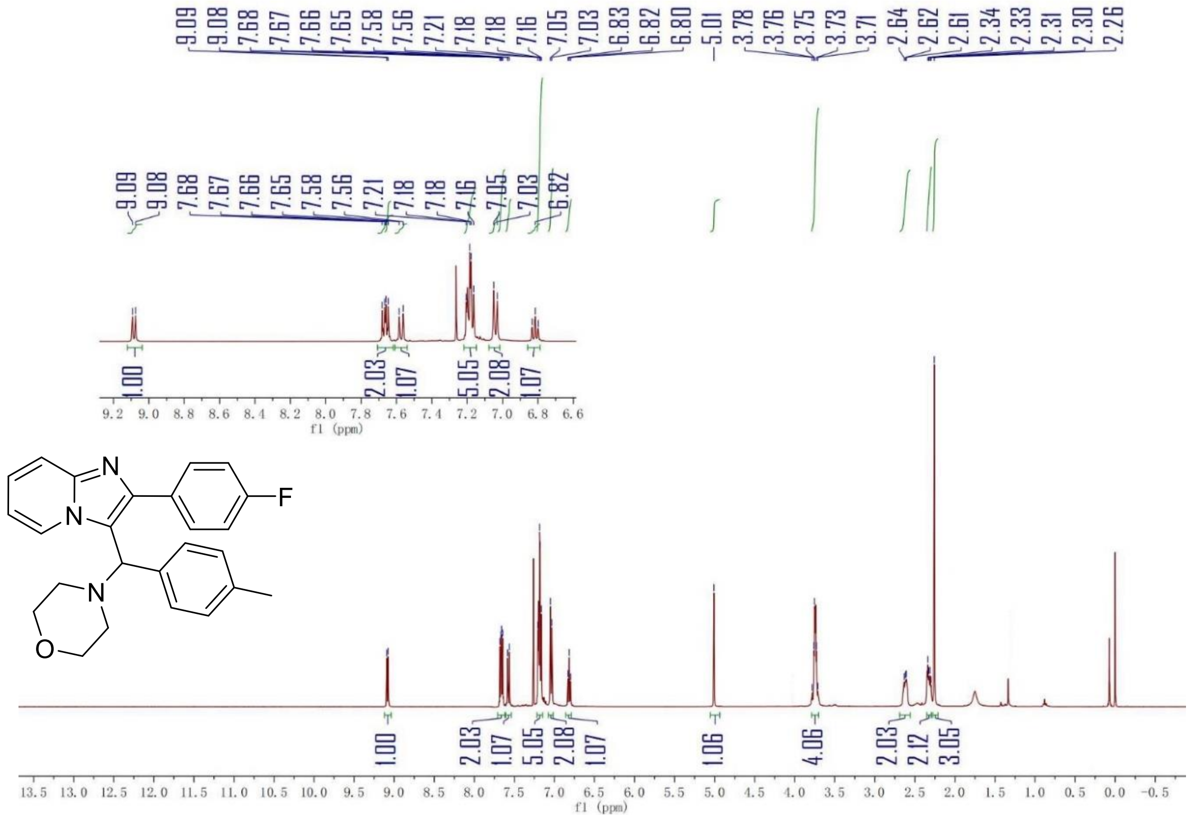

^{1}H NMR spectrum of compound **4m**

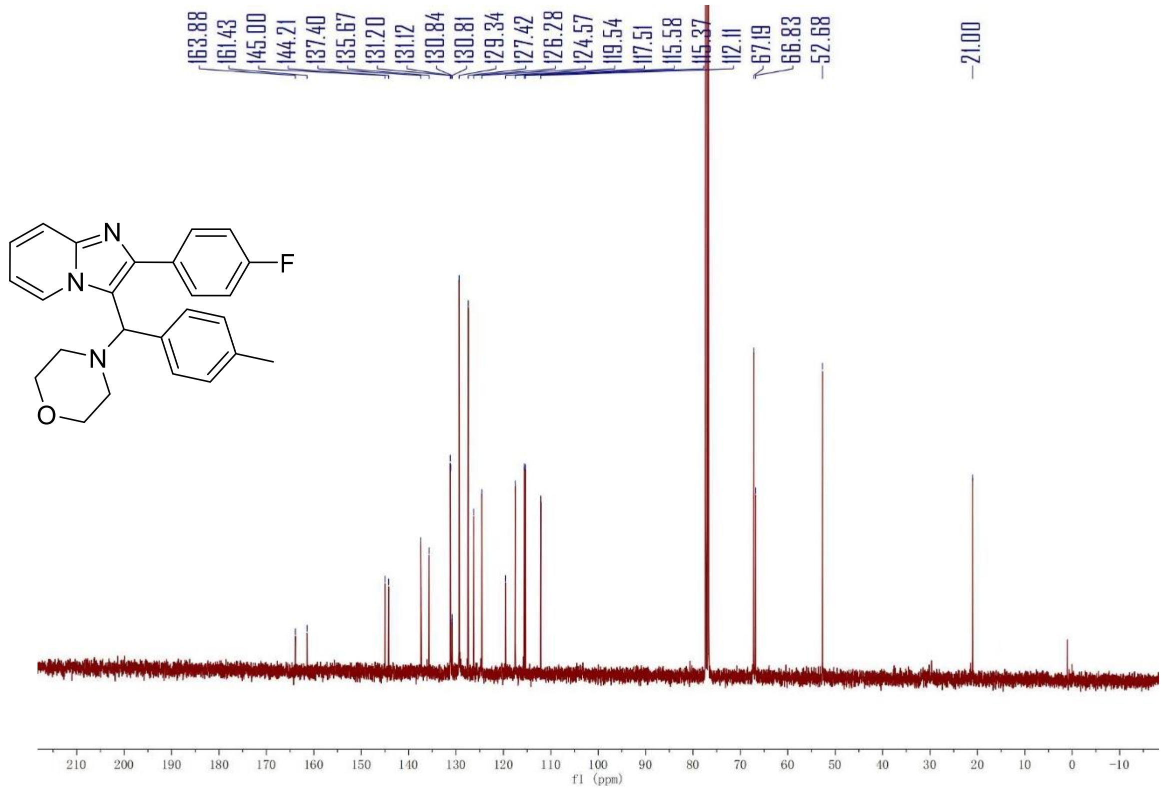

^{13}C NMR spectrum of compound **4m**

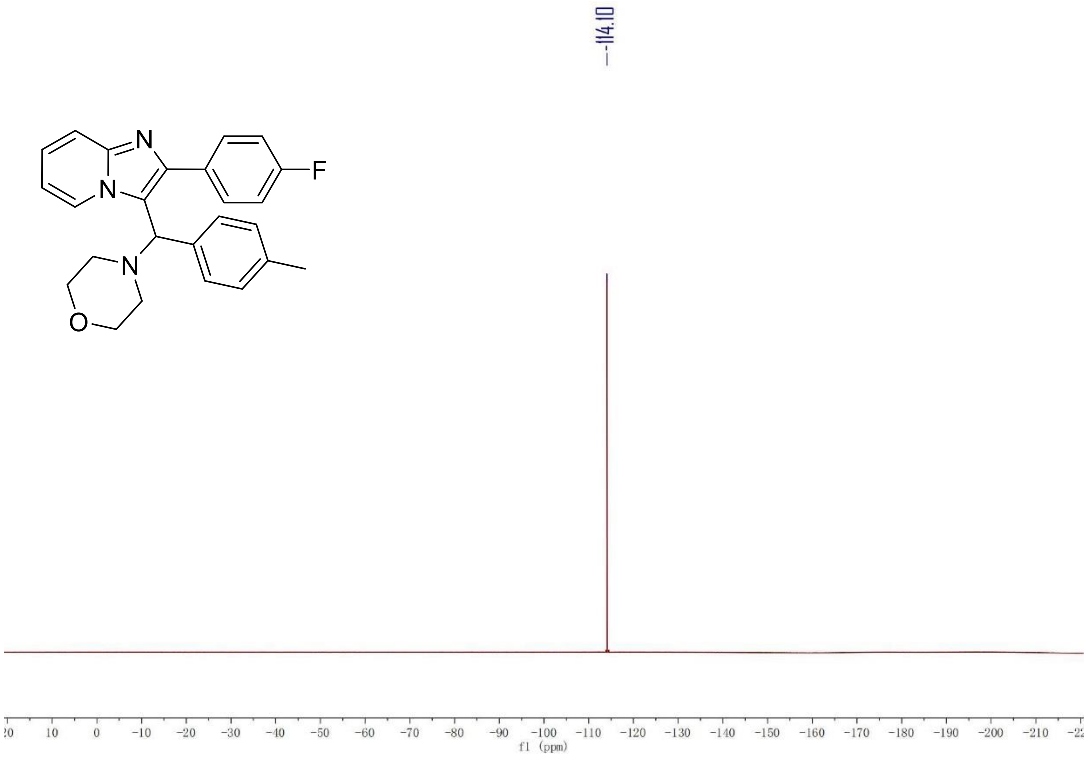

^{19}F NMR spectrum of compound **4m**

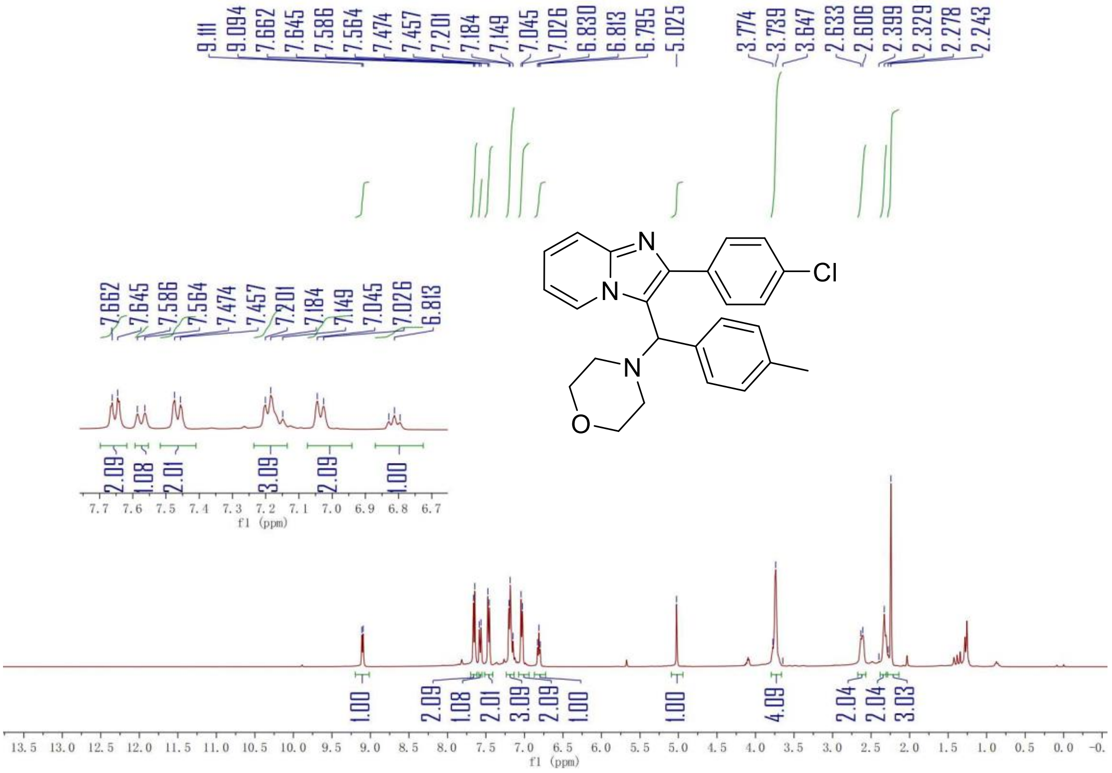

^{1}H NMR spectrum of compound **4n**

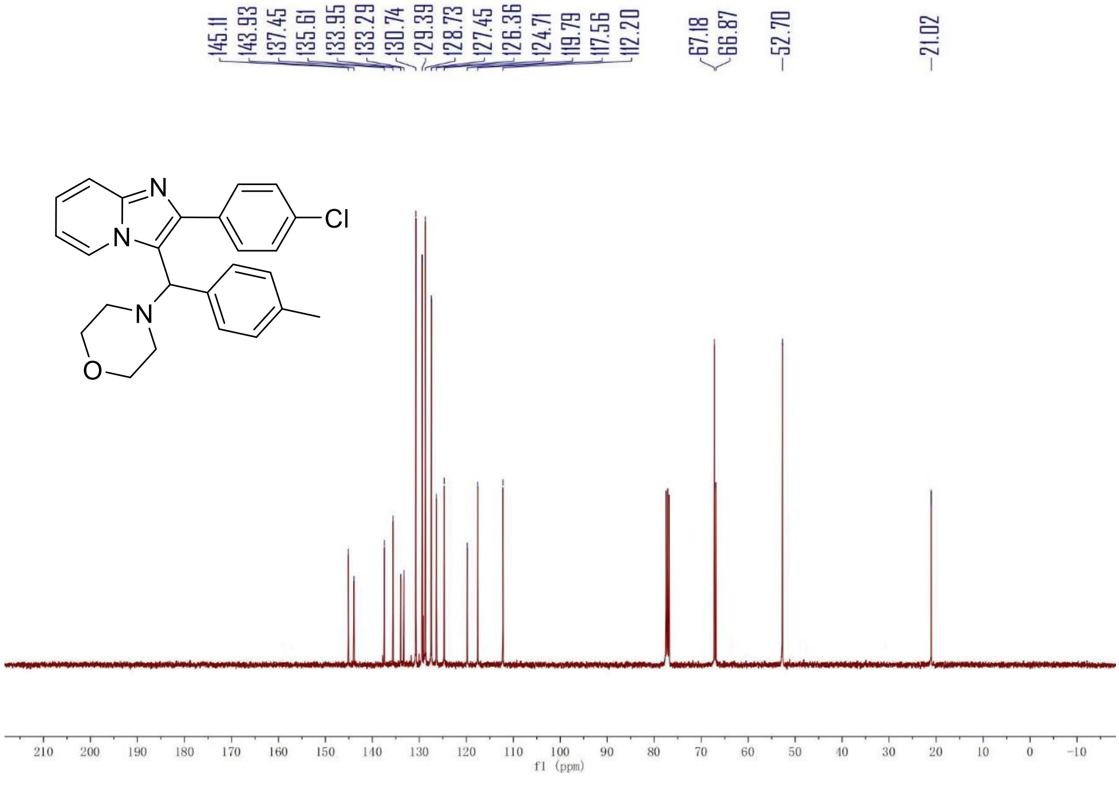

^{13}C NMR spectrum of compound **4n**

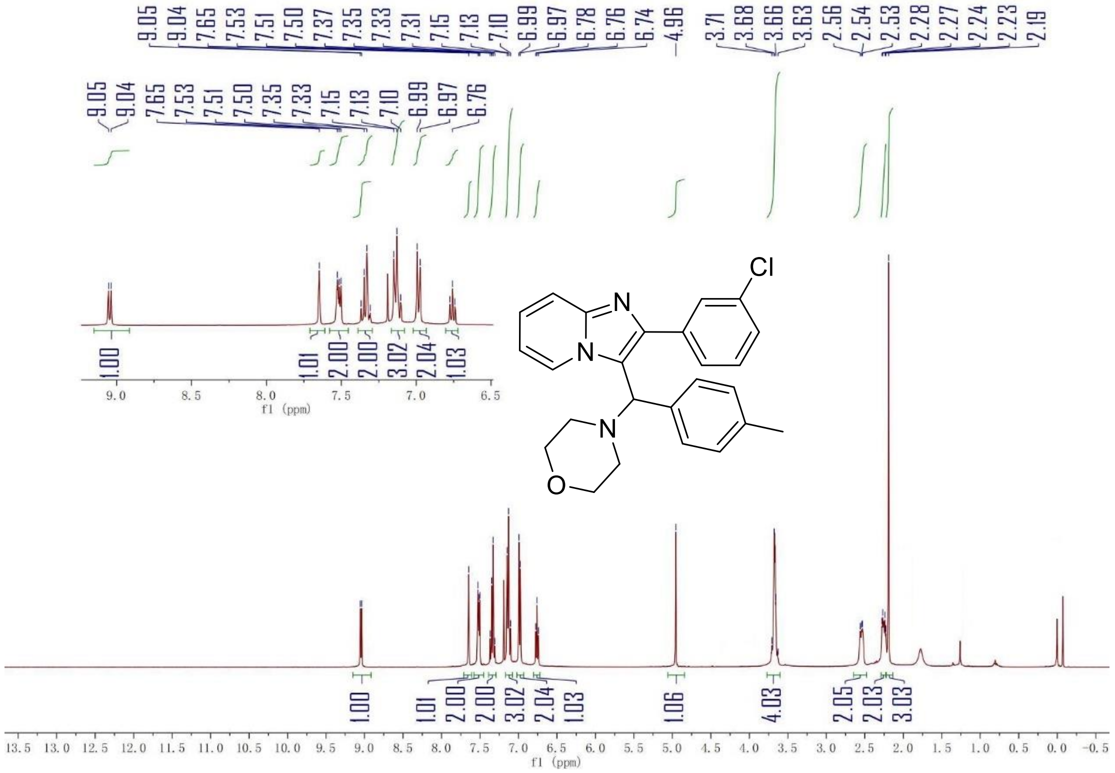

^{1}H NMR spectrum of compound **4o**

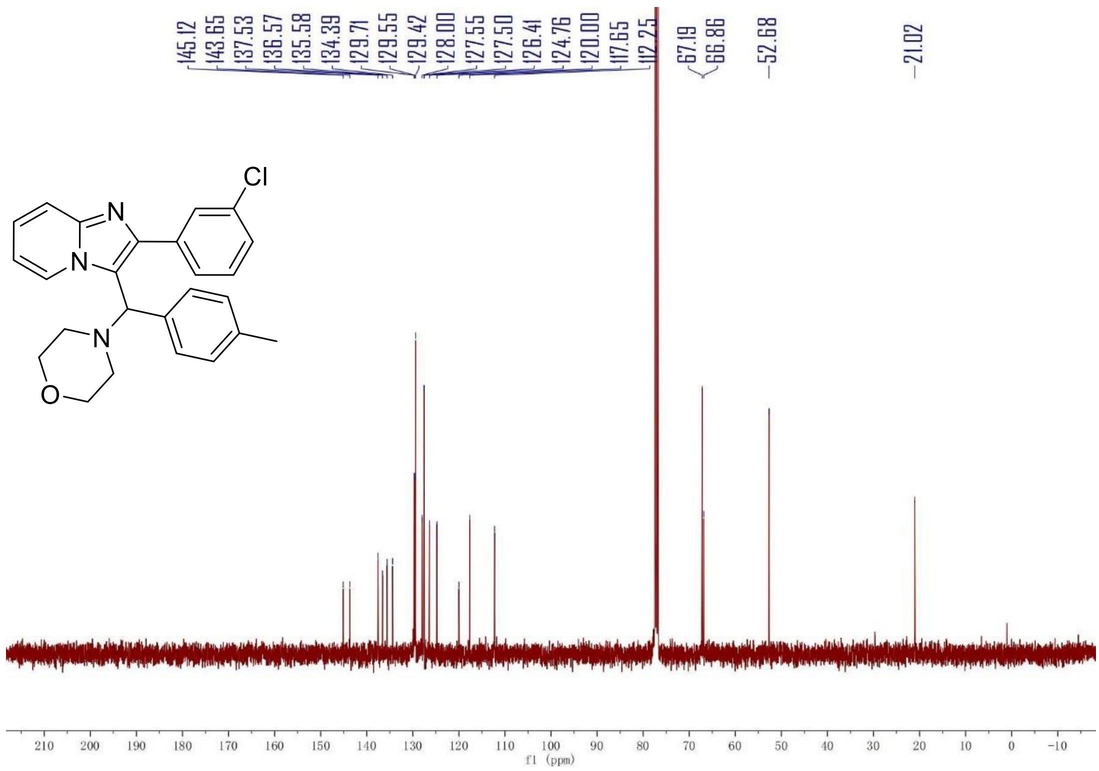

^{13}C NMR spectrum of compound **4o**

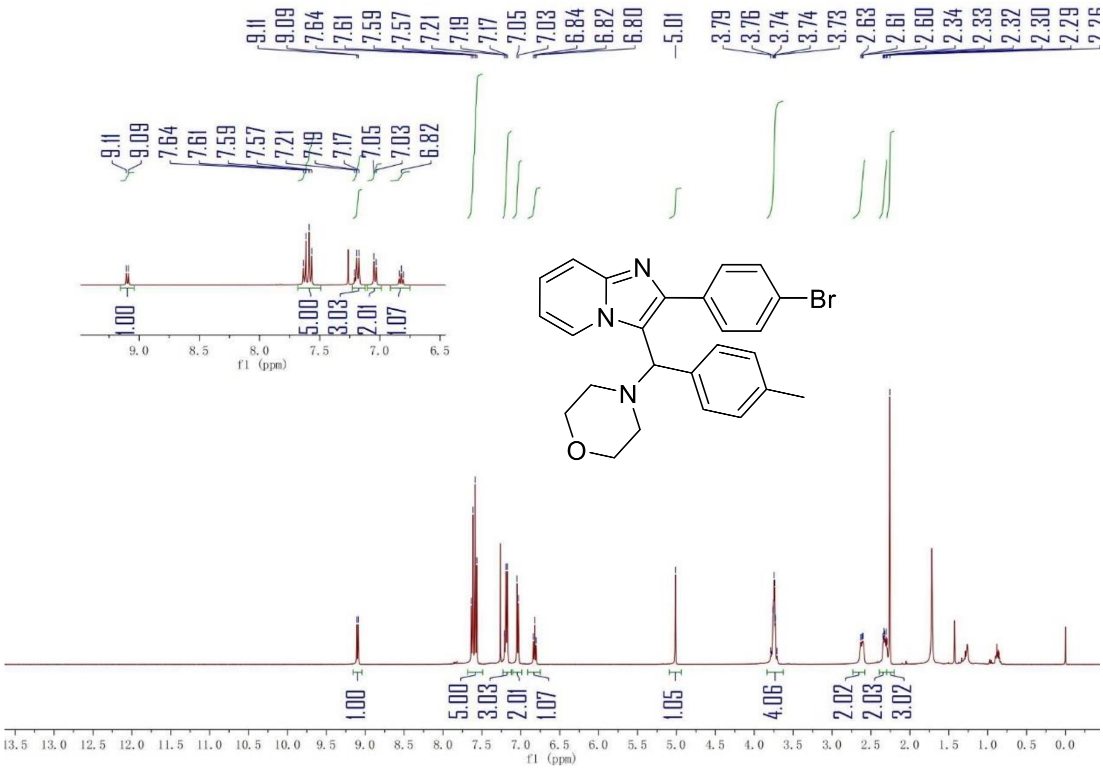

^{1}H NMR spectrum of compound **4p**

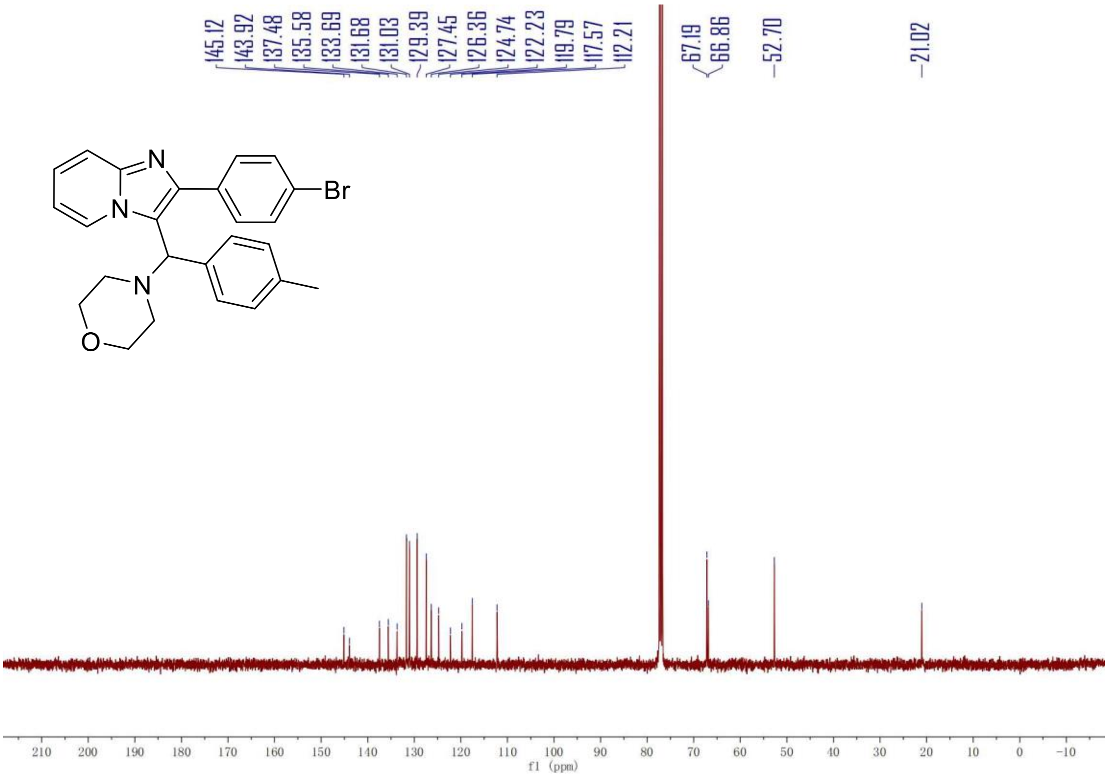

^{13}C NMR spectrum of compound **4p**

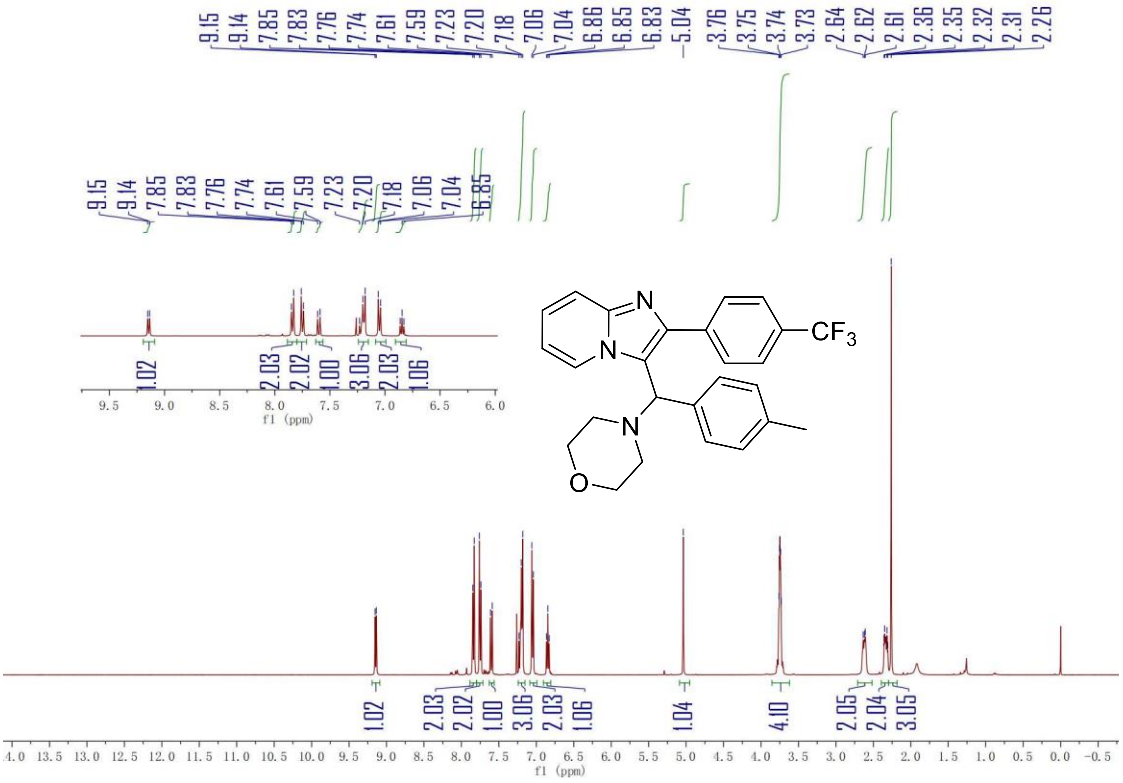

^{1}H NMR spectrum of compound **4q**

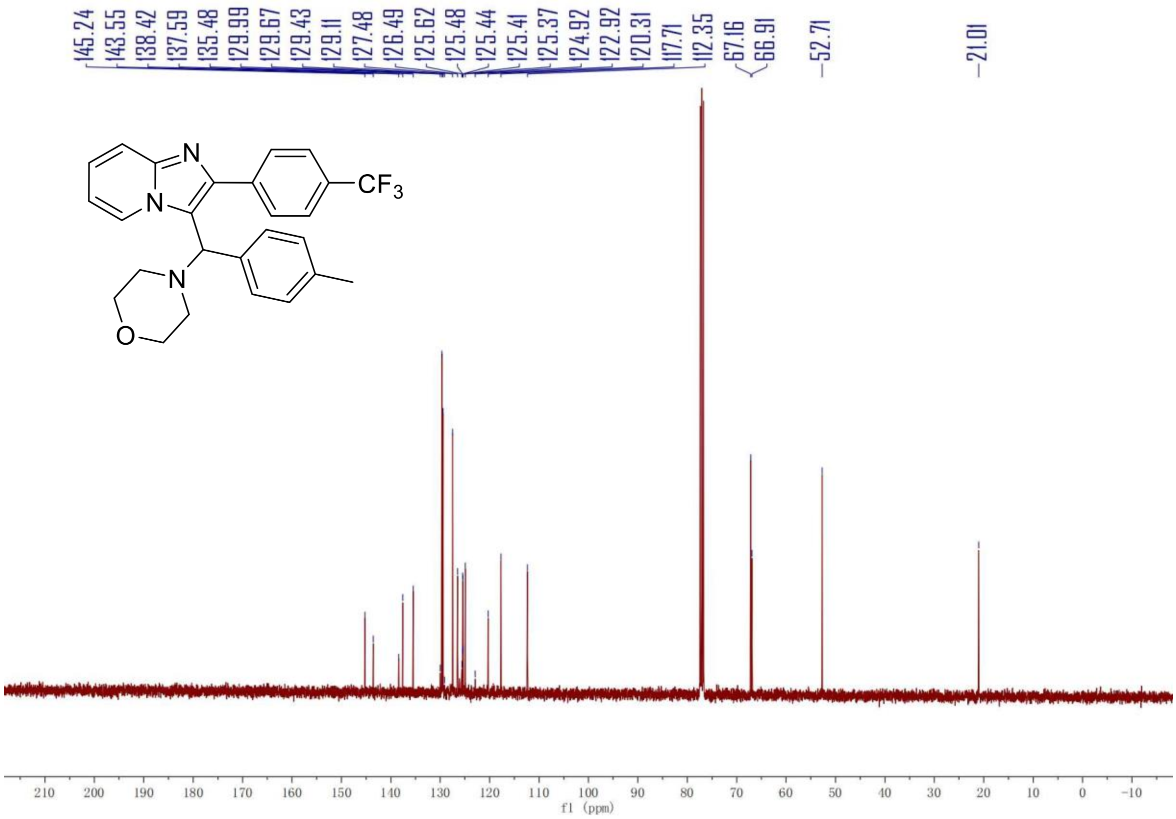

^{13}C NMR spectrum of compound **4q**

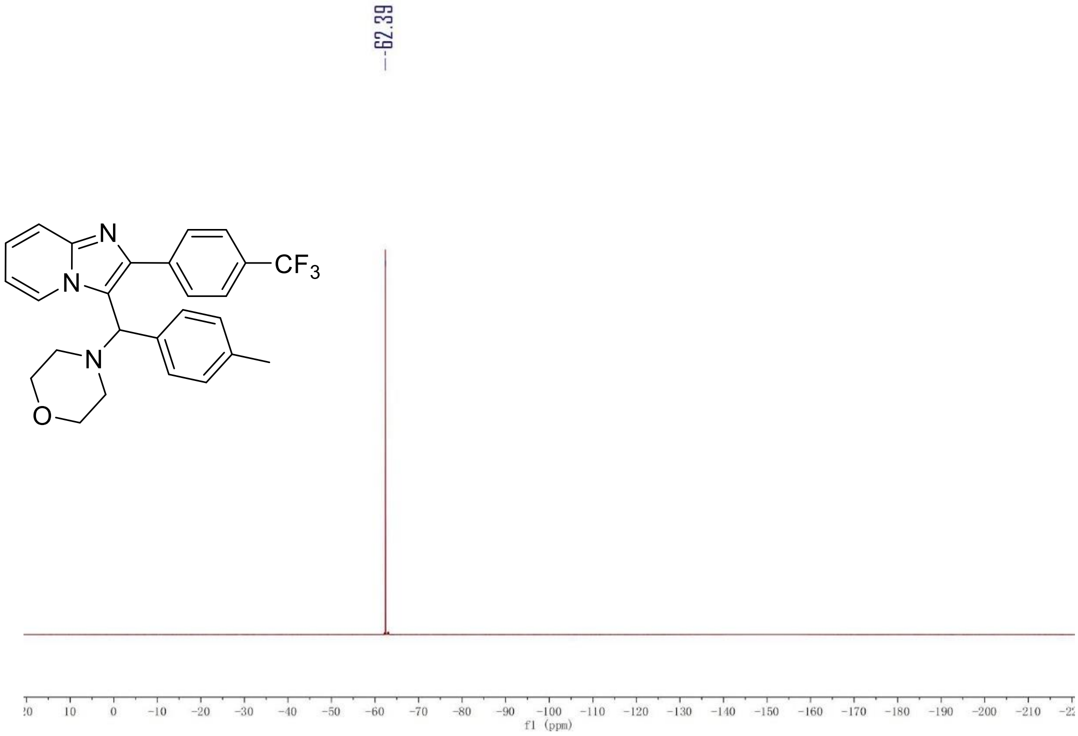

^{19}F NMR spectrum of compound **4q**

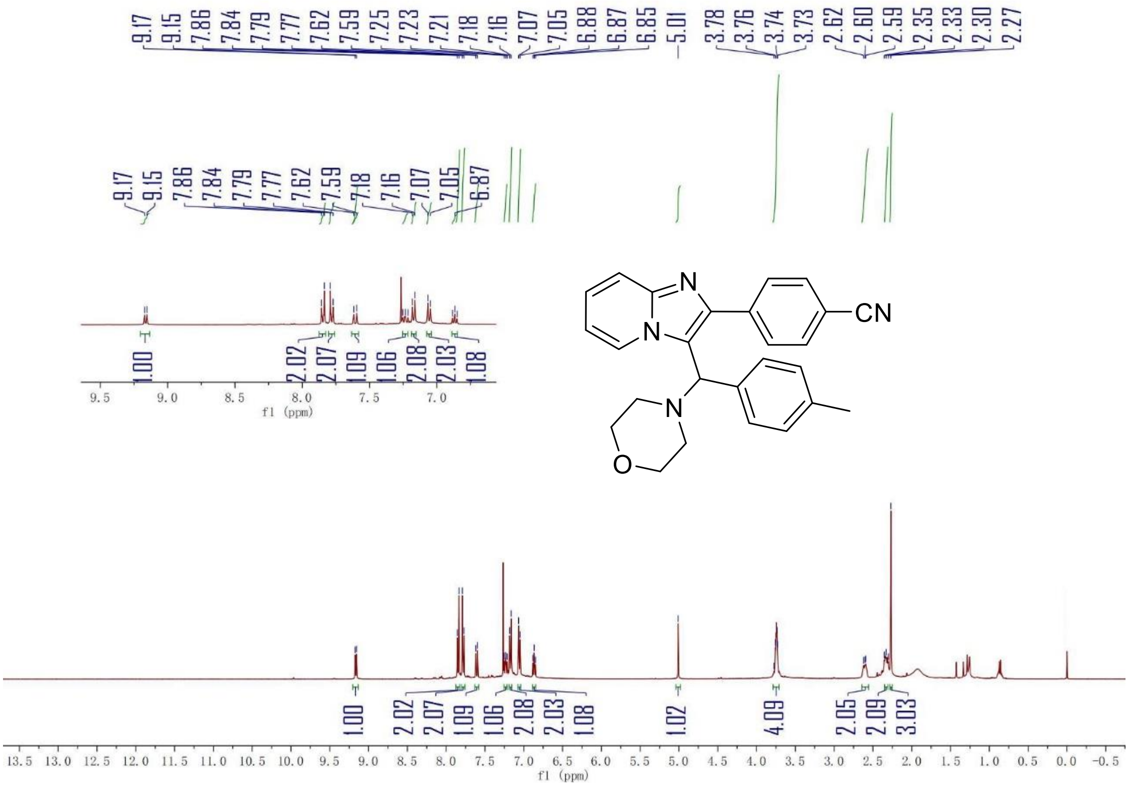

^{1}H NMR spectrum of compound **4r**

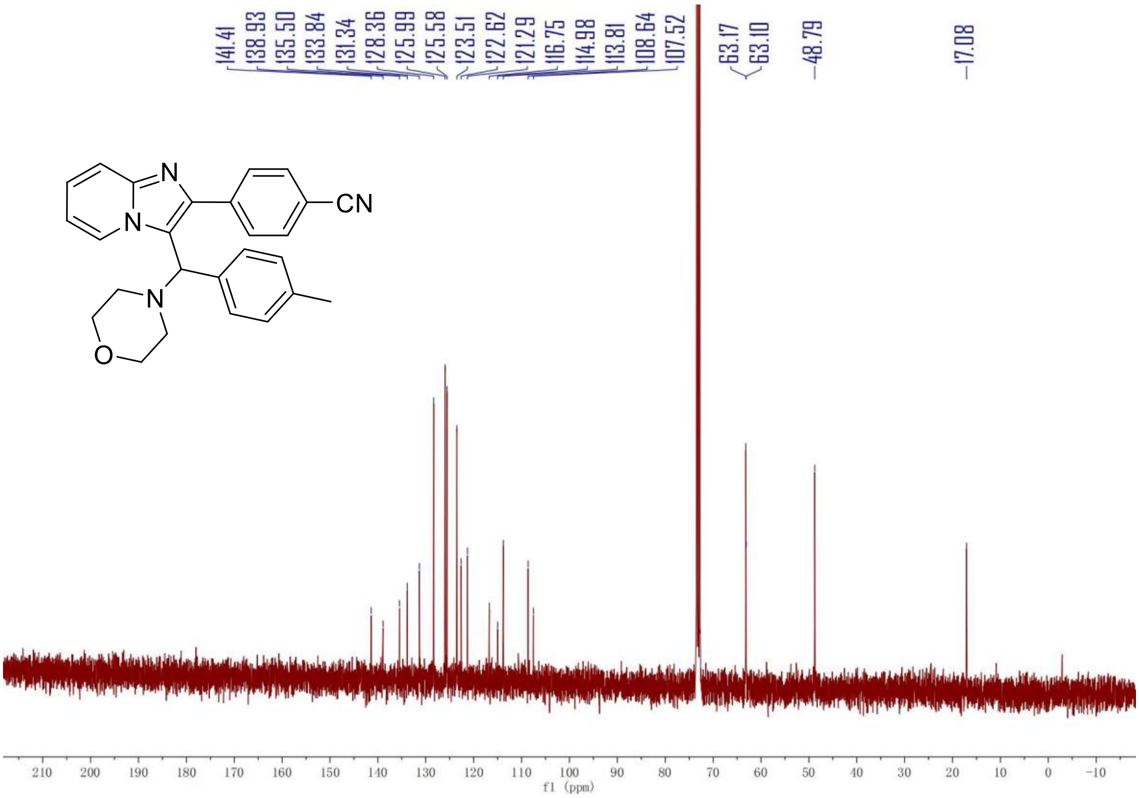

^{13}C NMR spectrum of compound **4r**

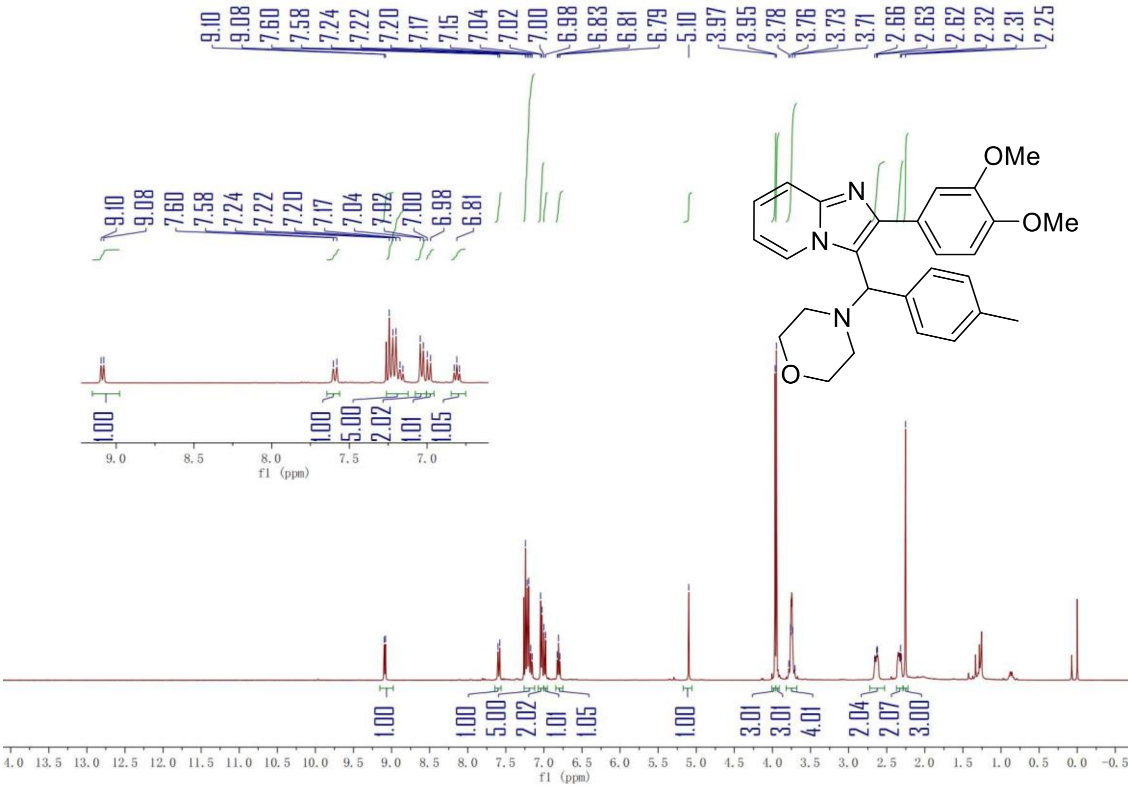

^{1}H NMR spectrum of compound **4s**

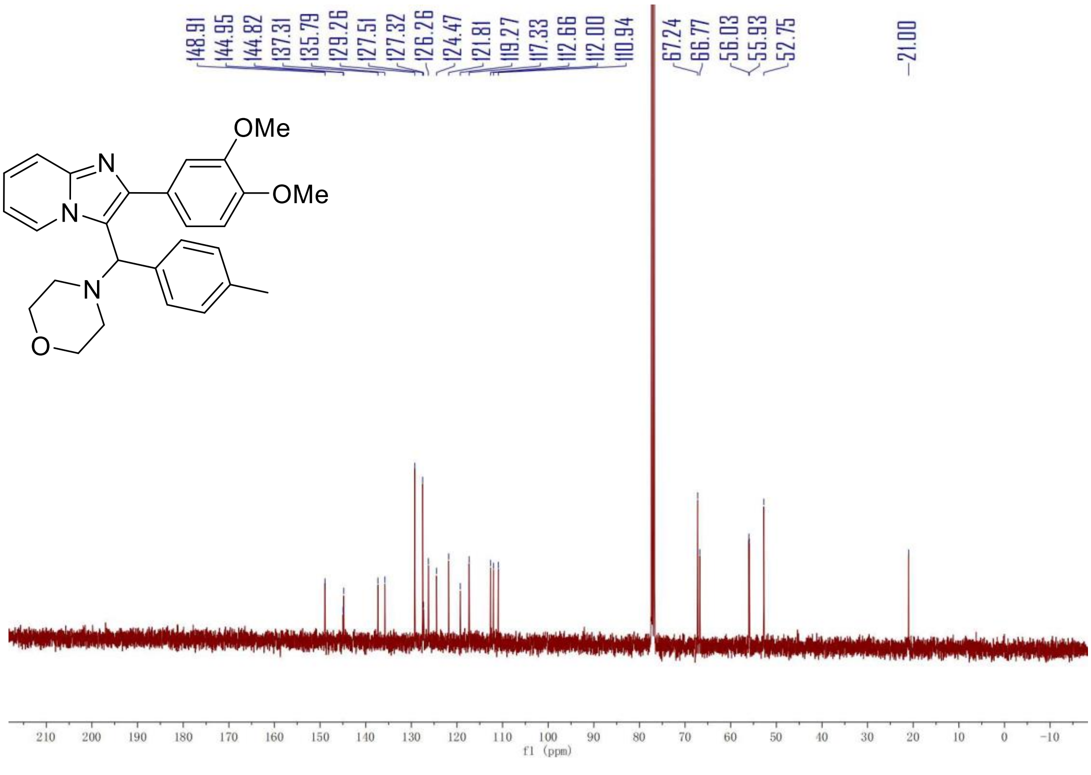

^{13}C NMR spectrum of compound **4s**

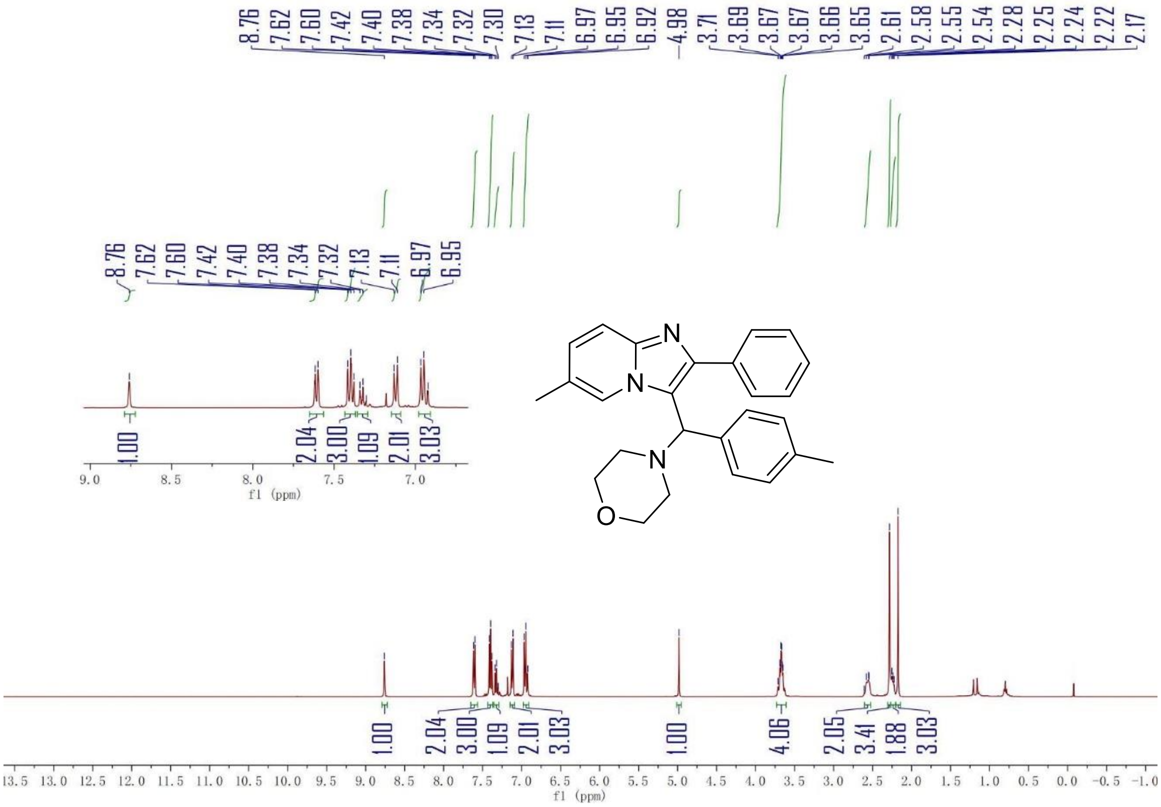

^{1}H NMR spectrum of compound **4t**

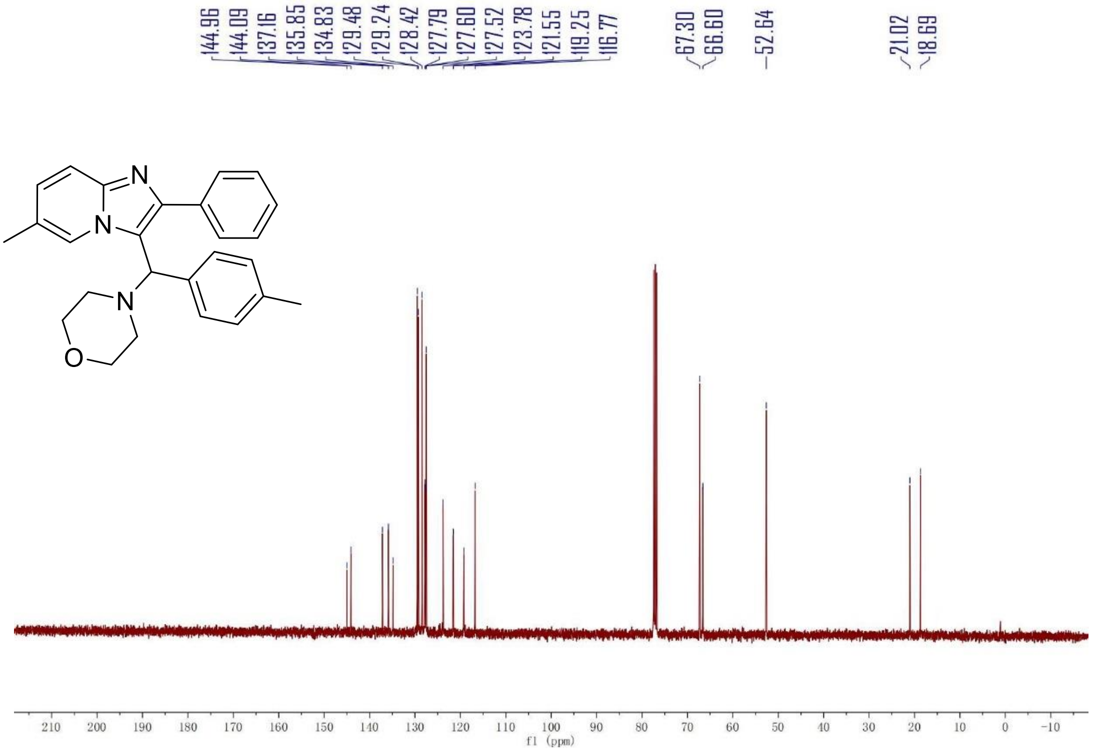

^{13}C NMR spectrum of compound **4t**

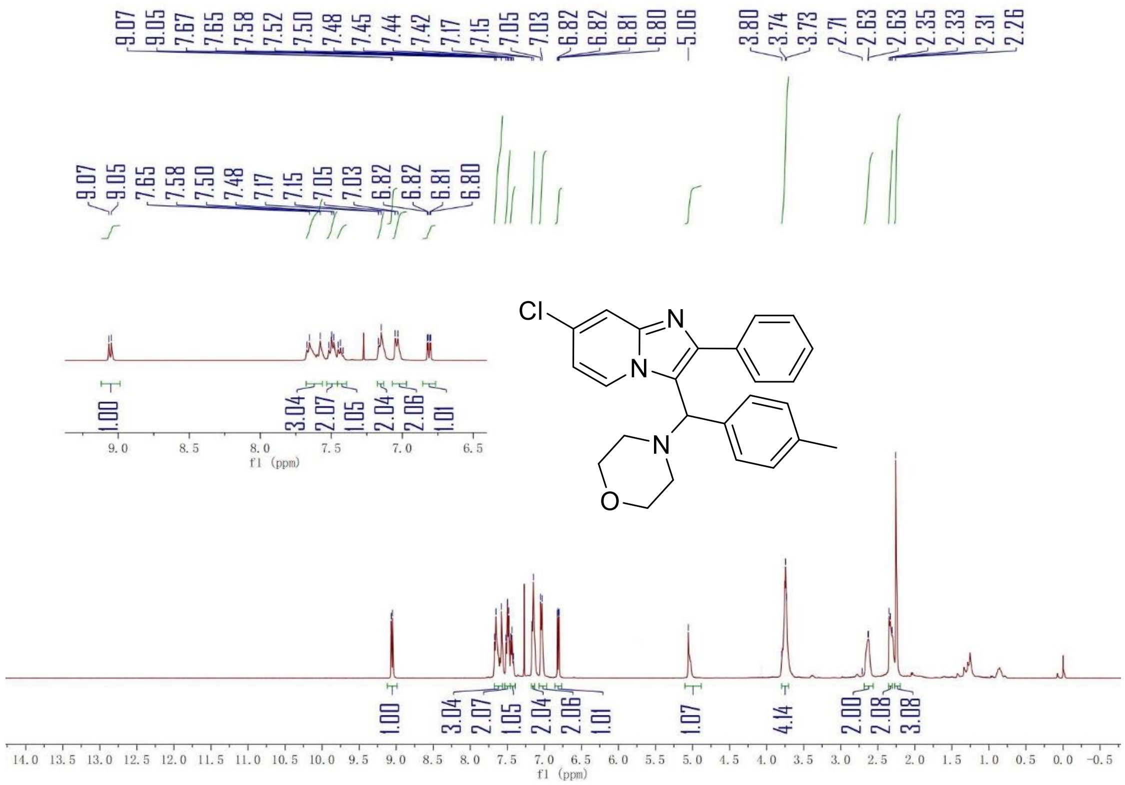

^{1}H NMR spectrum of compound **4u**

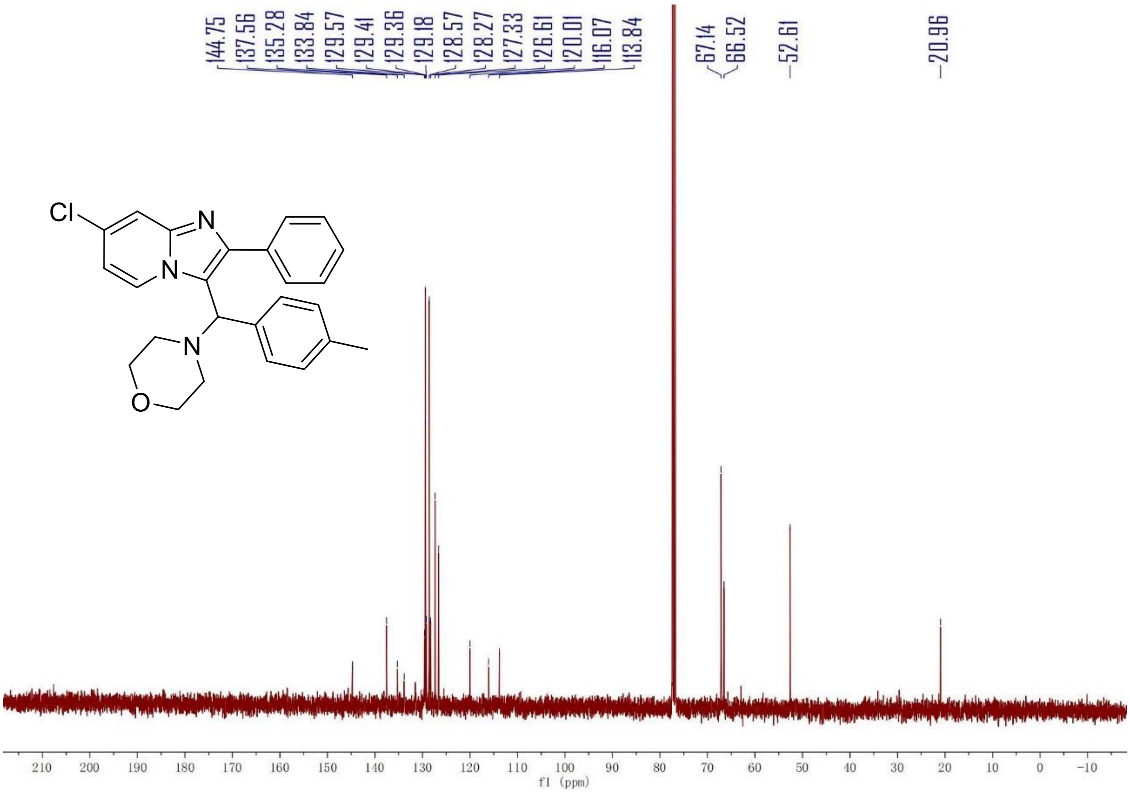

^{13}C NMR spectrum of compound **4u**

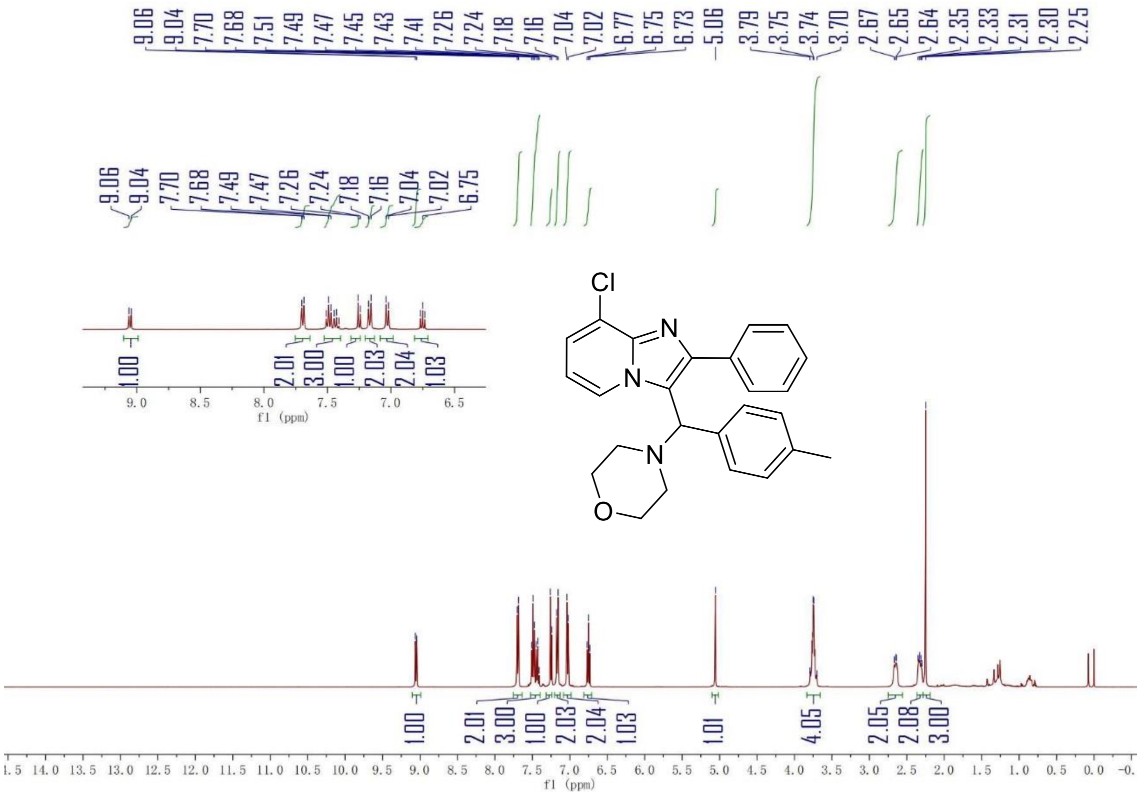

^{1}H NMR spectrum of compound **4v**

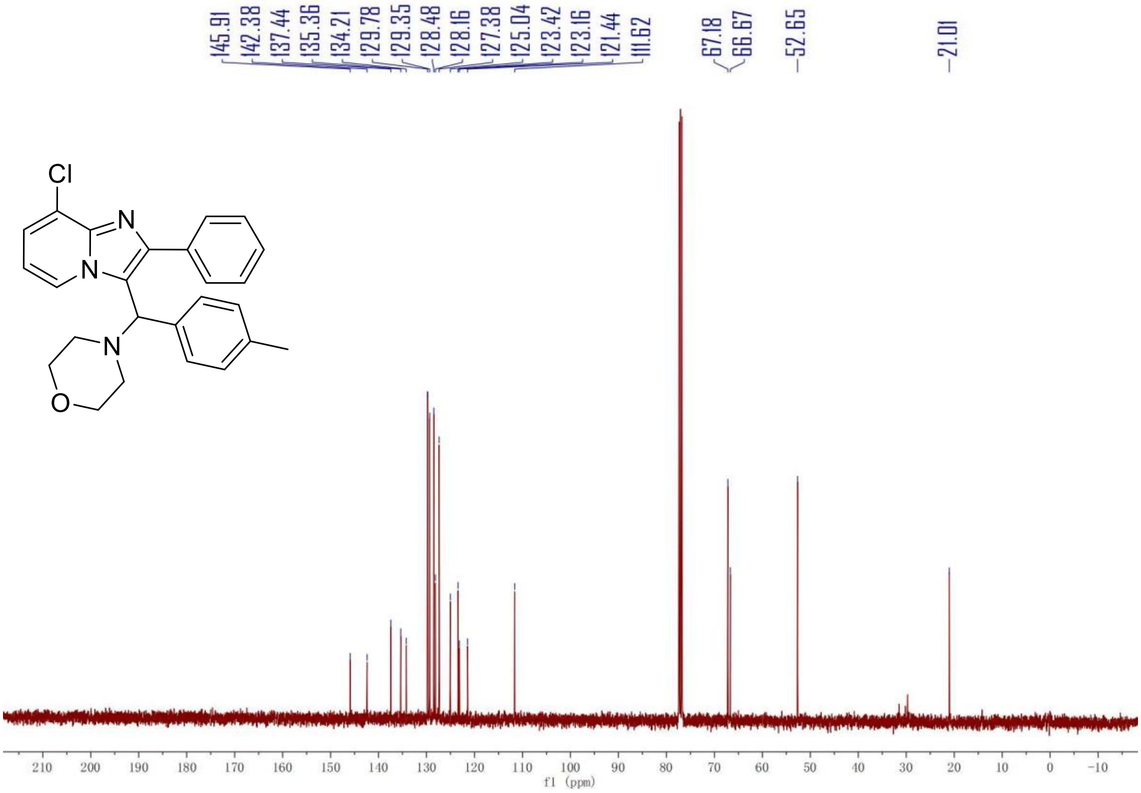

^{13}C NMR spectrum of compound **4v**

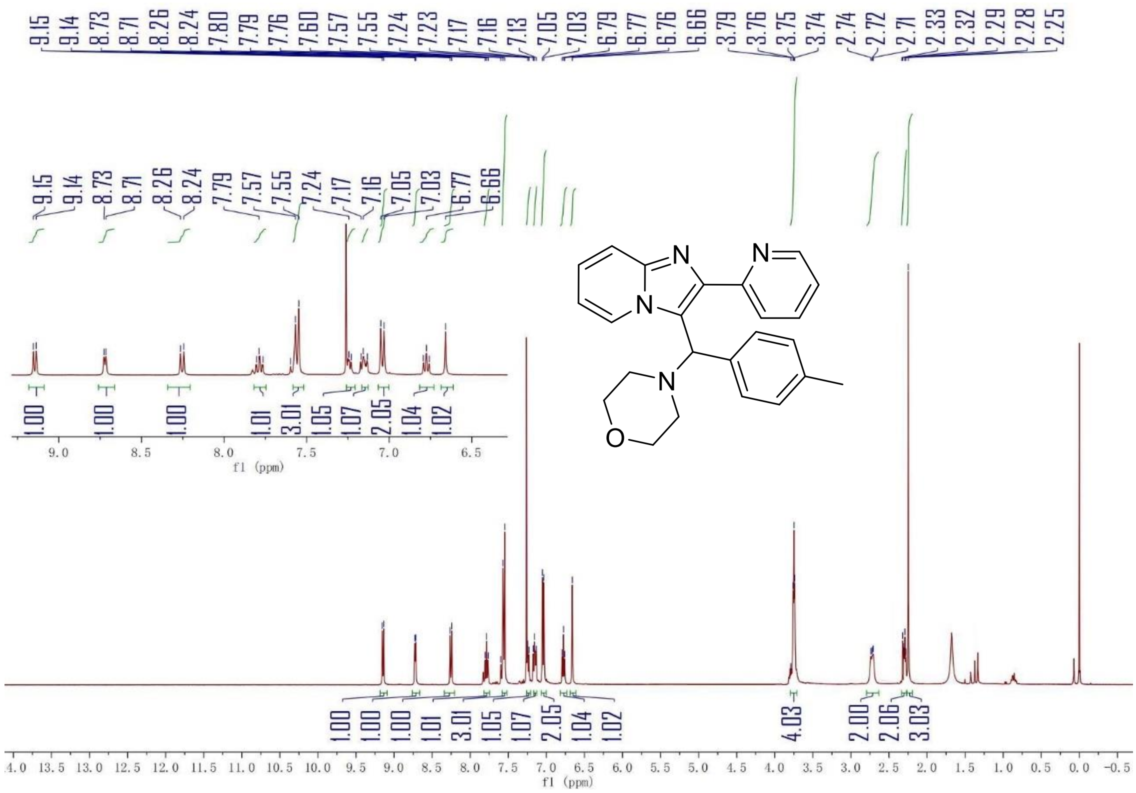

^{1}H NMR spectrum of compound **4w**

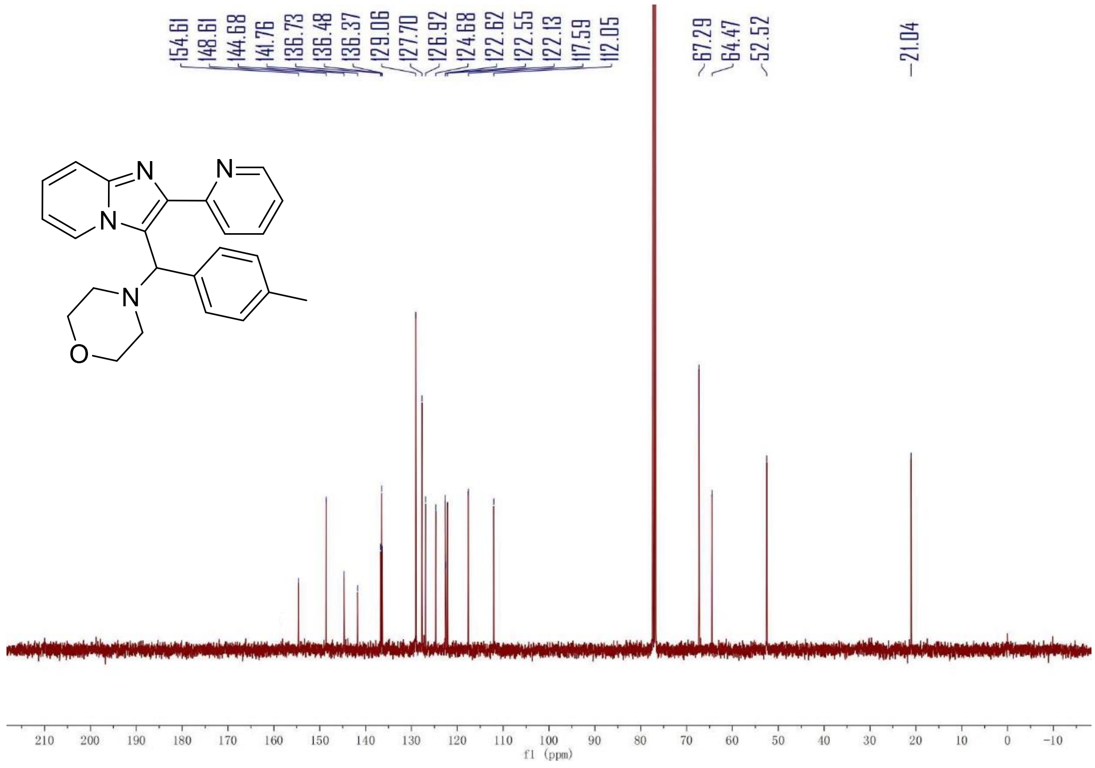

^{13}C NMR spectrum of compound **4w**

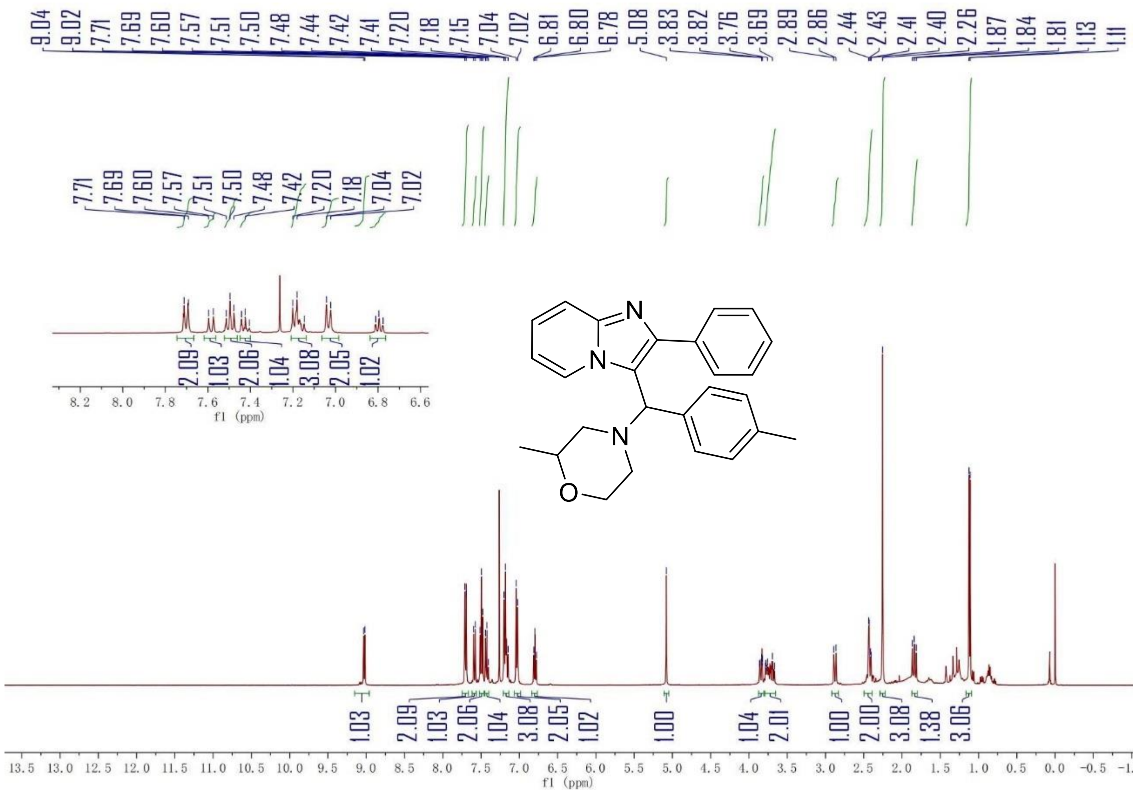

^{1}H NMR spectrum of compound **4x**

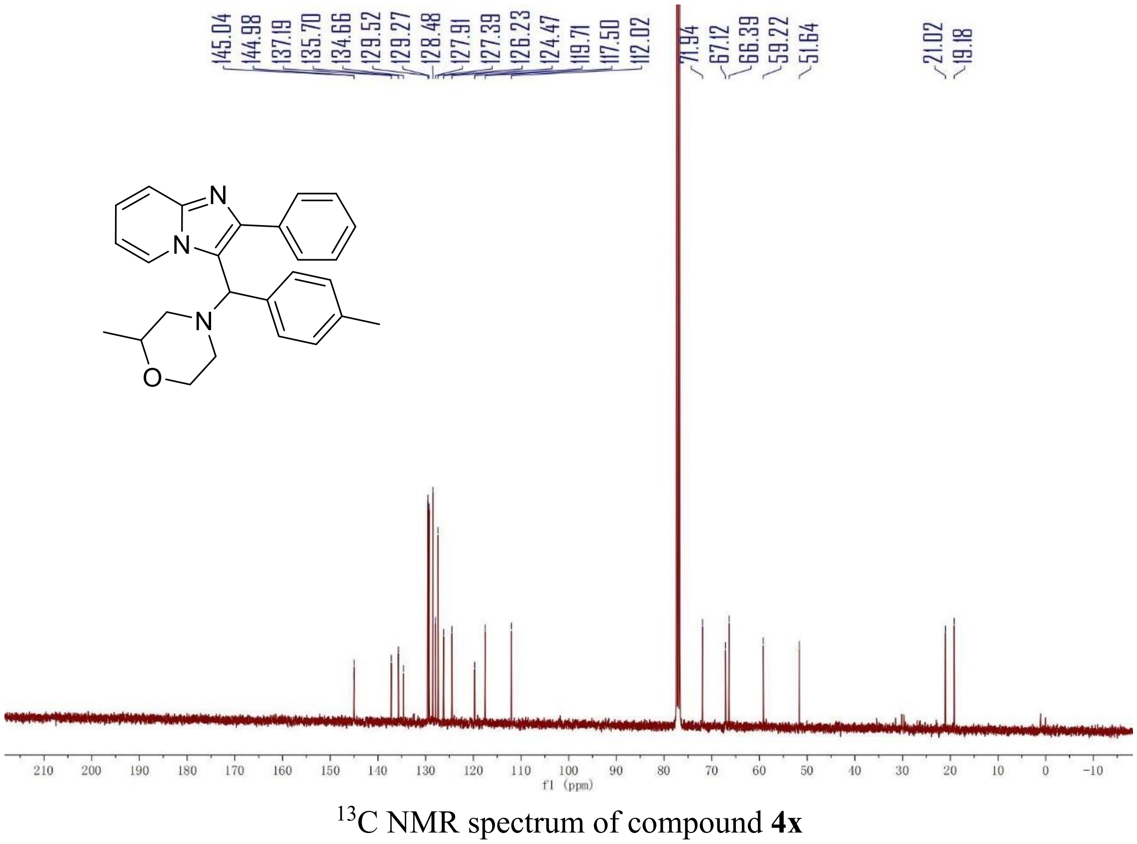

^{13}C NMR spectrum of compound **4x**

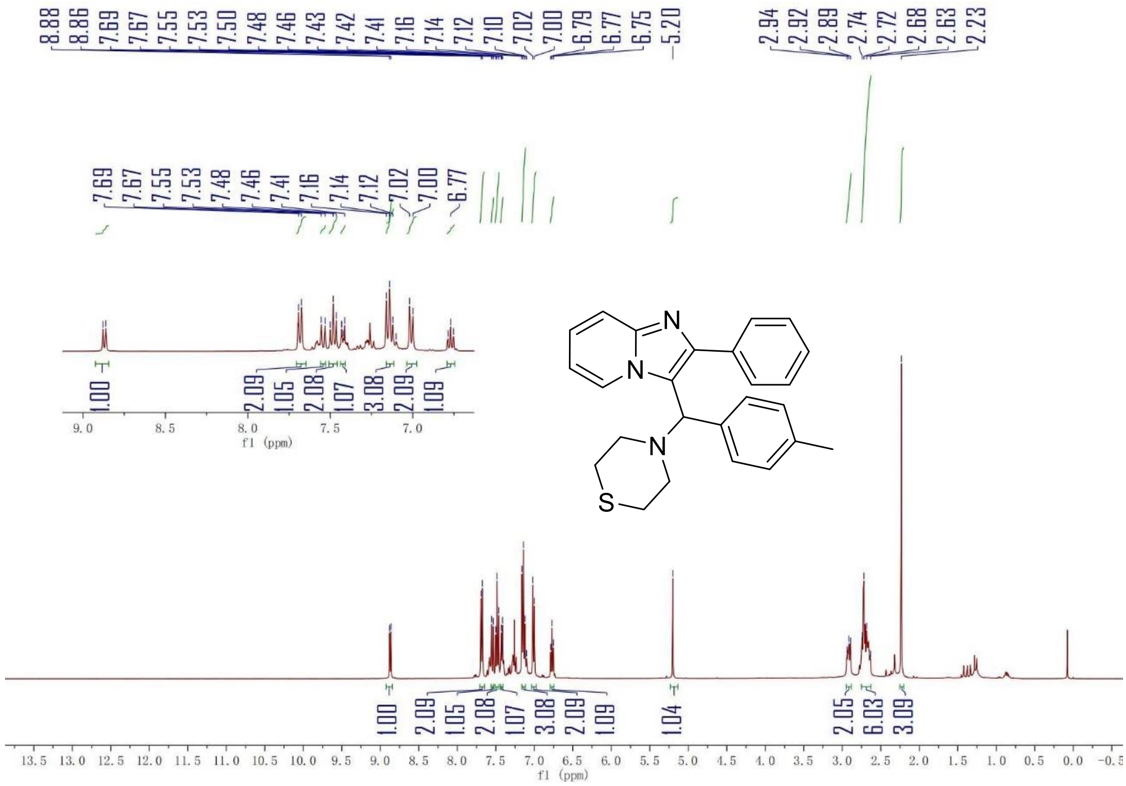

^{1}H NMR spectrum of compound **4y**

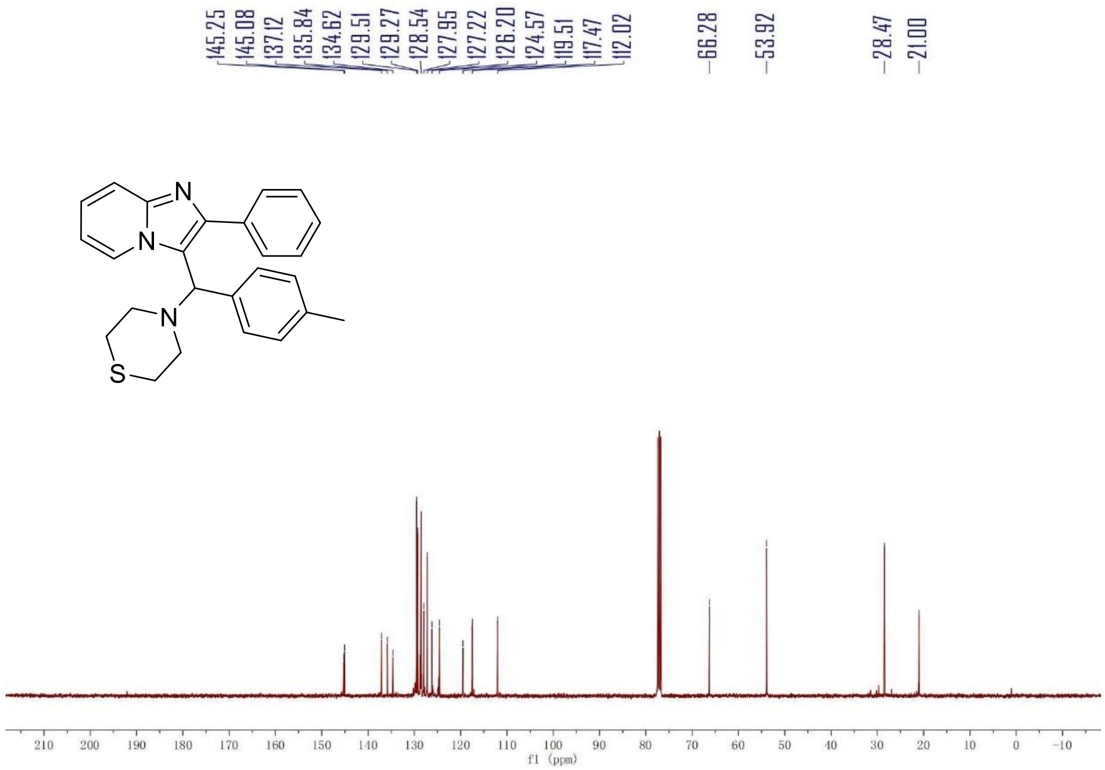

^{13}C NMR spectrum of compound **4y**

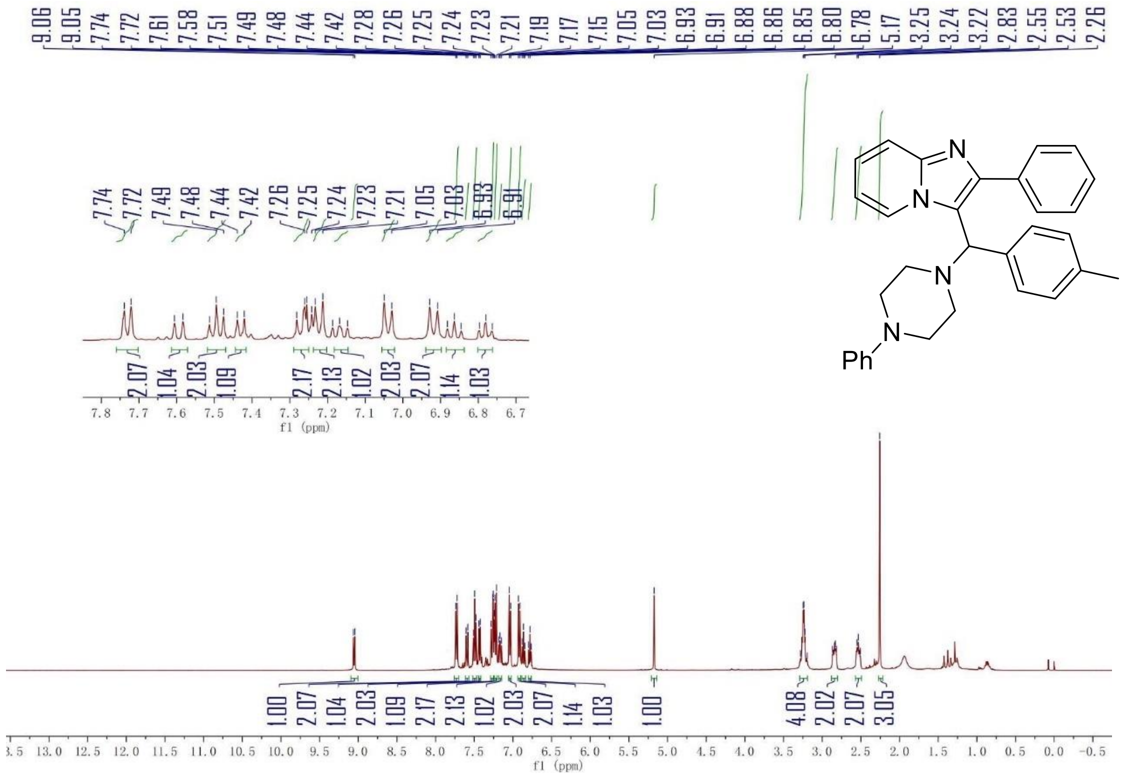

^{1}H NMR spectrum of compound **4z**

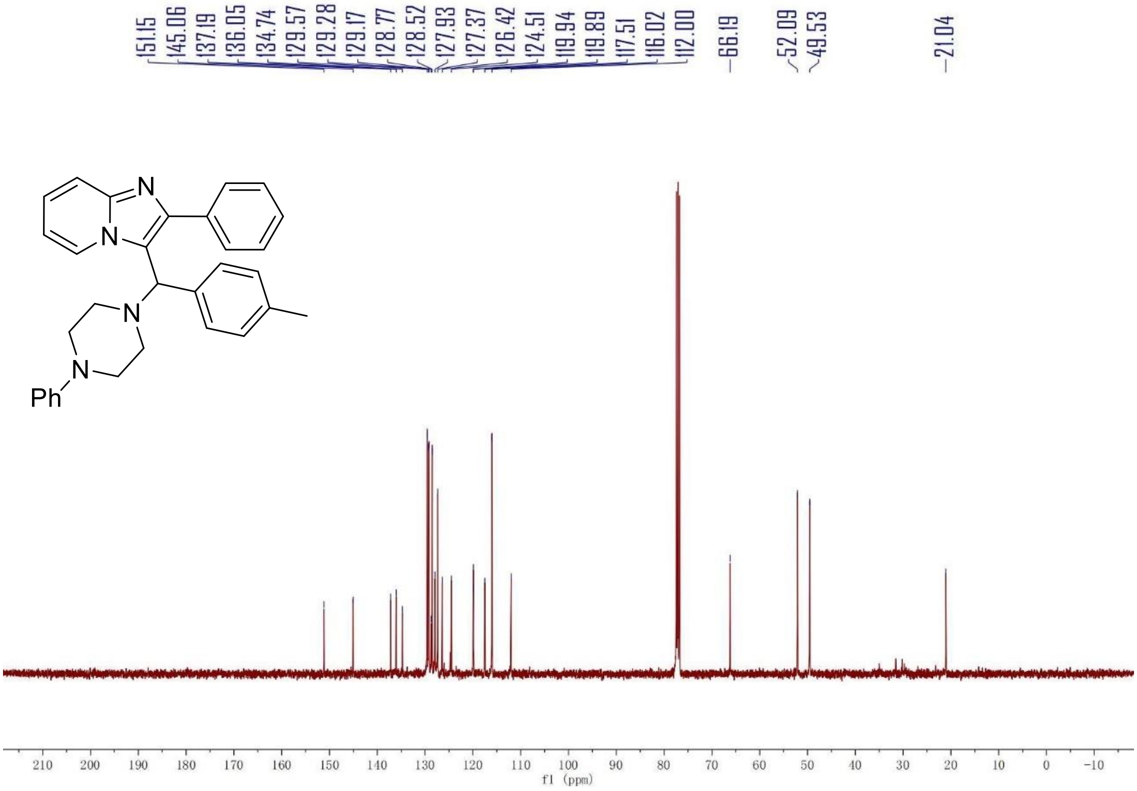

^{13}C NMR spectrum of compound **4z**

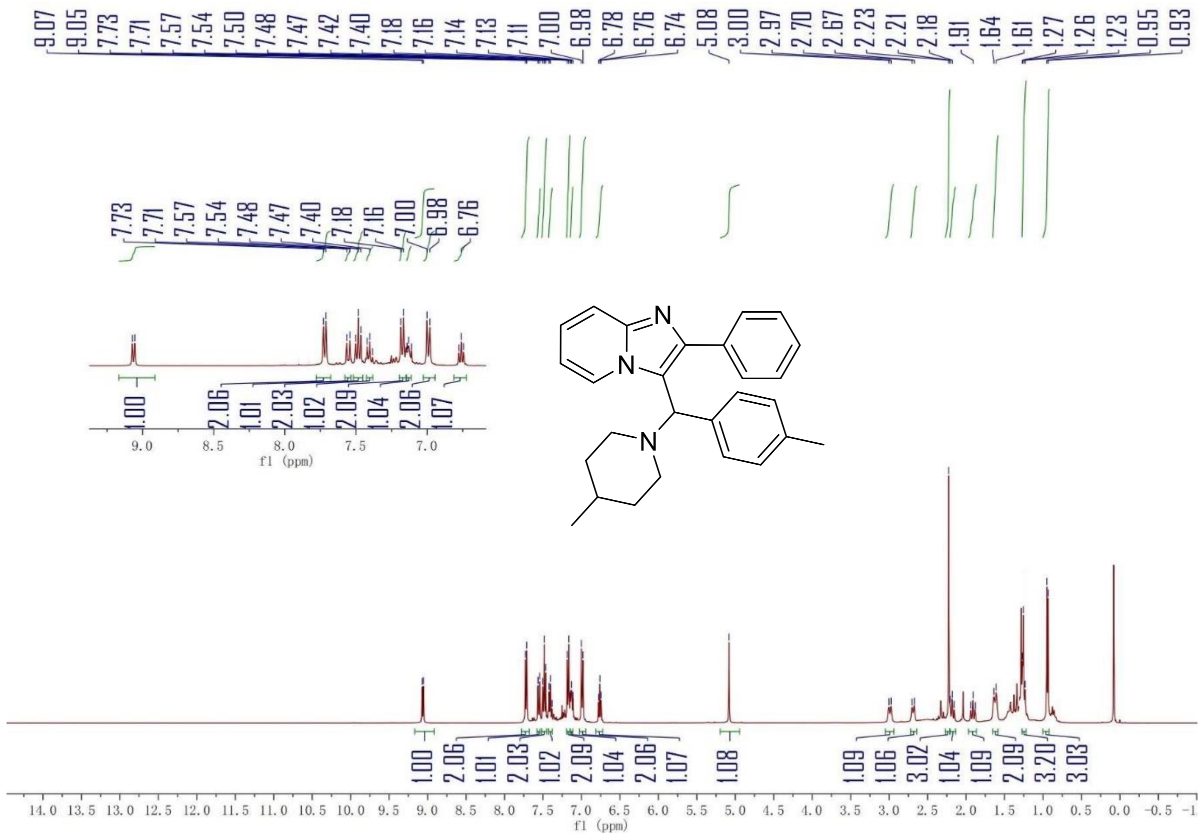

^{1}H NMR spectrum of compound **4aa**

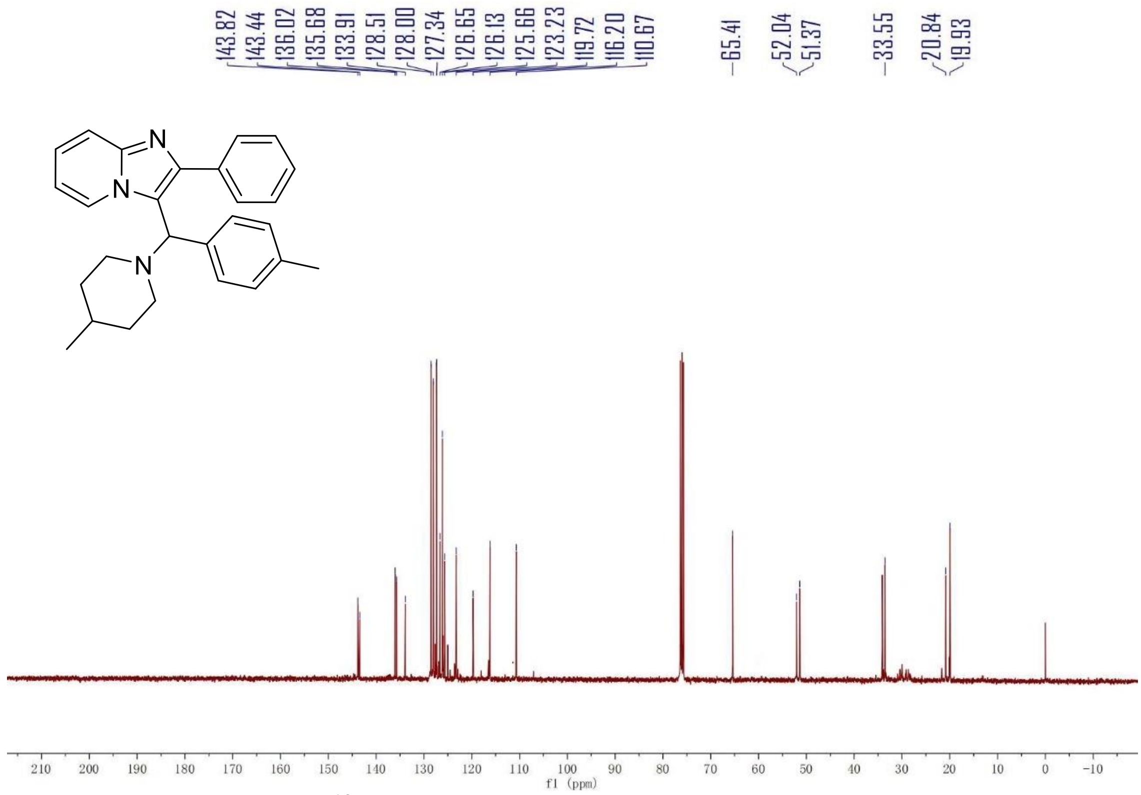

^{13}C NMR spectrum of compound **4aa**

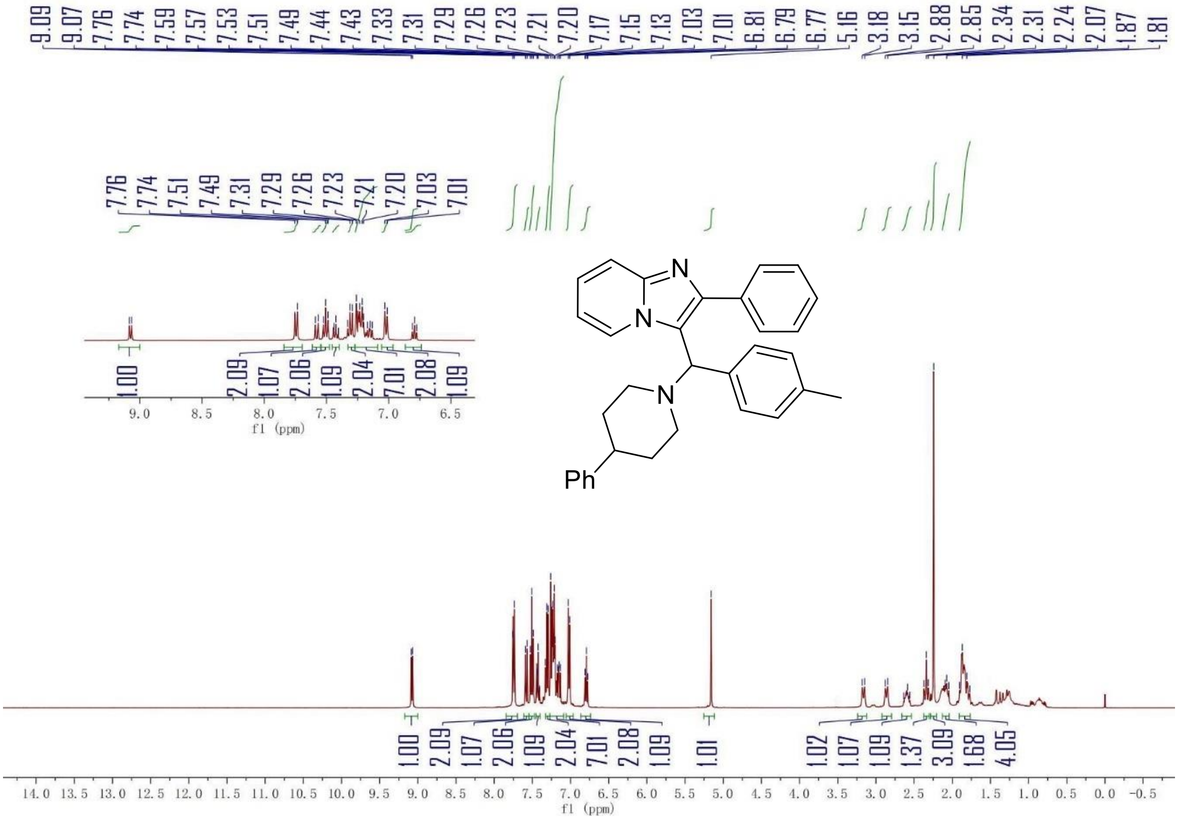

^{1}H NMR spectrum of compound **4ab**

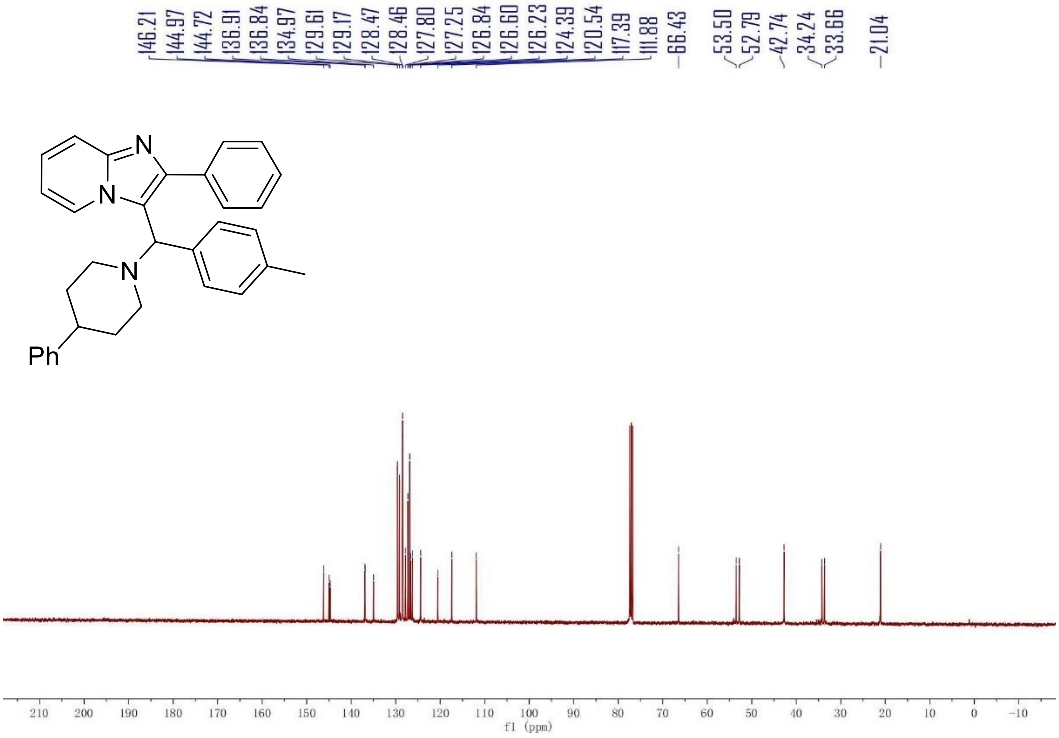

^{13}C NMR spectrum of compound **4ab**

References

[1] Obermayer, D.; Znidar, D.; Glotz, G.; Stadler, A.; Dallinger, D.; Kappe, C. O. Design and performance validation of a conductively heated sealed-vessel reactor for organic Synthesis. *J. Org. Chem*. **2016**, *81*, 11788-11801.

[2] Ghosh, P.; Ganguly, B.; Kar, B.; Dwivedi, S.; Das, S. Green procedure for highly efficient, rapid synthesis of imidazo[1,2-a]pyridine and its late stage functionalization. *Synth. Commun*. **2018**, *48*, 1076.
